# Nuclear receptor E75/NR1D2 promotes tumor malignant transformation by integrating Hippo and Notch pathways

**DOI:** 10.1038/s44318-024-00290-3

**Published:** 2024-11-08

**Authors:** Xianping Wang, Yifan Guo, Peng Lin, Min Yu, Sha Song, Wenyan Xu, Du Kong, Yin Wang, Yanxiao Zhang, Fei Lu, Qi Xie, Xianjue Ma

**Affiliations:** 1 Westlake Laboratory of Life Sciences and Biomedicine, Hangzhou, 310024 Zhejiang China; 2 School of Life Sciences, Westlake University, Hangzhou, 310024 Zhejiang China; 3 Institute of Biomedicine and Biotechnology, Shenzhen Institute of Advanced Technology, Chinese Academy of Sciences, Shenzhen, China; 4 State Key Laboratory of Chemical Oncogenomics, Key Laboratory of Chemical Genomics, Peking University Shenzhen Graduate School, Shenzhen, 518055 China; 5 Present Address: Department of Diabetes & Cancer Metabolism, Beckman Research Institute of City of Hope National Medical Center, Duarte, CA 91010 USA

**Keywords:** Steroid Hormone, Hippo Signaling, Notch Signaling, NR1D2, Glioblastoma, Cancer, Chromatin, Transcription & Genomics, Signal Transduction

## Abstract

Hormone therapy resistance and the ensuing aggressive tumor progression present a significant clinical challenge. However, the mechanisms underlying the induction of tumor malignancy upon inhibition of steroid hormone signaling remain poorly understood. Here, we demonstrate that *Drosophila* malignant epithelial tumors show a similar reduction in ecdysone signaling, the main steroid hormone pathway. Our analysis of ecdysone-induced downstream targets reveals that overexpression of the nuclear receptor E75, particularly facilitates the malignant transformation of benign tumors. Genome-wide DNA binding profiles and biochemistry data reveal that E75 not only binds to the transcription factors of both Hippo and Notch pathways, but also exhibits widespread co-binding to their target genes, thus contributing to tumor malignancy. We further validated these findings by demonstrating that depletion of *NR1D2*, the mammalian homolog of E75, inhibits the activation of Hippo and Notch target genes, impeding glioblastoma progression. Together, our study unveils a novel mechanism by which hormone inhibition promotes tumor malignancy, and describes an evolutionarily conserved role of the oncogene E75/NR1D2 in integration of Hippo and Notch pathway activity during tumor progression.

## Synopsis

The interplay between hormone signaling and malignant transformation remains incompletely understood. This study identifies E75/NR1D2 as an evolutionarily conserved oncogene that promotes malignant transformation of benign tumors by co-regulating the Hippo and Notch signaling pathways.

- The ecdysone steroid hormone signaling is inhibited in malignant *Drosophila* tumors.
- Overexpression of nuclear receptor *E75* facilitates malignant tumor transformation.
- E75 promotes tumor malignancy by integrating Hippo pathway and Notch pathway signaling at the transcription factor level.
- The mammalian E75 ortholog also regulates the Hippo and Notch pathways and promotes glioblastoma progression.

## Abstract (web summary)

The conserved steroid-hormone-induced transcription factor E75/NR1D2 interacts with Hippo and Notch pathway transcription factors to co-regulate their activity.

## Graphical abstract

**Figure:**
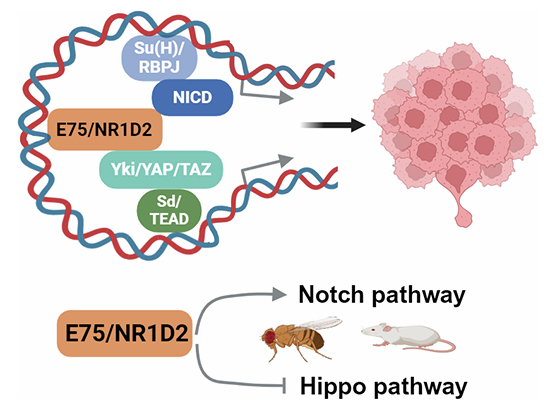

**Figure:**
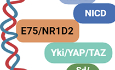

## Introduction

Hormones play a pivotal role in controlling cell division and growth, and their dysregulation can lead to uncontrolled cell proliferation and tumor formation (Conway-Campbell et al, 2007; Metcalfe et al, 2018; Xu et al, 2021). Many hormones can function as growth factors, thereby stimulating cell proliferation. For instance, estrogen has been shown to promote the growth of hormone receptor-positive breast cancer cells, and women who had menopausal hormone therapy shortly after menopause have been found to have a significantly increased risk of developing invasive breast cancer (Kotsopoulos, 2019). Similarly, the male sex hormone androgens can stimulate prostate cancer cells to grow through the activation of androgen receptors. Consequently, hormone therapy is widely employed as a treatment strategy for both breast and prostate cancers, yielding highly effective initial responses in terms of impeding tumor growth (Asemota et al, 2022; Caffa et al, 2020; Metcalfe et al, 2018). However, it is important to note that the development of resistance to hormone therapy poses a significant clinical problem (Hanker et al, 2020; Metcalfe et al, 2018). In the case of breast cancer, around 30–40% of patients experience this resistance (Anurag et al, 2018). More strikingly, most male patients undergoing androgen deprivation therapy eventually progress to advanced castration-resistant prostate cancer (CRPC) (Buttigliero et al, 2015). The resistant tumor cells have increased proliferation ability and become more aggressive, and patients have a worse survival outcome (Brankovic-Magic et al, 2002; Rani et al, 2019). Nonetheless, the underlying mechanisms that trigger the malignant transformation in hormone-signaling-reduced tumor cells remain largely unexplored.

*Drosophila melanogaster* is a widely used model organism in cancer research due to its genetic tractability and the high degree of conservation of key cancer-related pathways (Bilder et al, 2021; Liu et al, 2022b; Verheyen, 2022; Villegas, 2019). The evolutionarily conserved Hippo and Notch signaling pathways, both initially discovered and characterized in *Drosophila*, play crucial roles in cell proliferation, differentiation, and apoptosis (Zheng and Pan, 2019; Zhou et al, 2022). The activation of the transcription complexes of Hippo and Notch pathways, namely the YAP/TAZ-TEAD and NICD-RBPJ-MAML complexes, respectively, promotes tumor progression and malignancy, including glioblastoma (GBM), a highly aggressive type of brain cancer (Orr et al, 2011; Purow et al, 2005). Previous studies have uncovered a complex interplay between these two pathways. For instance, activation of YAP/TAZ can increase the expression of Notch receptors and ligands, thereby activating Notch signaling (Slemmons et al, 2017). Conversely, Notch signaling can inhibit the Hippo pathway to increase the activity of YAP/TAZ, thus promoting cell proliferation and inhibiting apoptosis (Kim et al, 2017). However, the precise molecular mechanisms underlying the integration of the Hippo and Notch pathways during cancer pathogenesis remain incompletely understood.

The hormonal system of *Drosophila*, while less complex than that of mammals, exhibits several similarities to its mammalian counterparts. As a result, *Drosophila* serves as a valuable tool in the field of hormonal research (Verheyen, 2022). The investigation of ecdysone, a primary steroid hormone in *Drosophila*, has shed light on its diverse roles in both physiological and pathological circumstances, ranging from the regulation of intestinal stem cell fate and morphogenesis to collective cell migration and tumor progression (Ahmed et al, 2020; Jiang et al, 2018; Santabárbara-Ruiz and Léopold, 2021; Wang et al, 2020; Zipper et al, 2020). Ecdysone binds to and activates ecdysone receptor (EcR), which in turn binds to specific regions of the DNA known as ecdysone response elements (EcREs), thus initiating a cascade of ecdysone-responsive transcription factor expression, including *Eip74EF*, *Eip75B*, *Eip93*, *Br*, *Hr3*, and *ftz-f1*, among others (Huet et al, 1995; Sullivan and Thummel, 2003; White et al, 1997). The early ecdysone-responsive gene *Eip75B* (*E75*) encodes a heme-binding nuclear receptor and has been implicated in various biological processes, including cell growth and differentiation (Ahmed et al, 2020; Reinking et al, 2005; Zipper et al, 2020), whereas the roles and mechanisms of *Eip75B* in tumorigenesis remain unknown.

In this study, we compared the transcriptome difference between benign and malignant *Drosophila* epithelium tumors and discovered that ecdysone steroid hormone signaling is specifically inhibited in malignant tumors. Furthermore, we demonstrated that ectopic expression of *E75* is sufficient to transform benign tumors into malignant ones. E75 drives cell proliferation and tumor malignancy by integrating Hippo and Notch signaling pathways at the transcription factor level. Additionally, we showed that *NR1D2*, the mammalian ortholog of *E75*, has a functionally and mechanically conserved role in regulating GBM progression in vivo.

## Results

### Ecdysone steroid hormone signaling is inhibited in *Drosophila* epithelial malignant tumor

**Figure 1:**
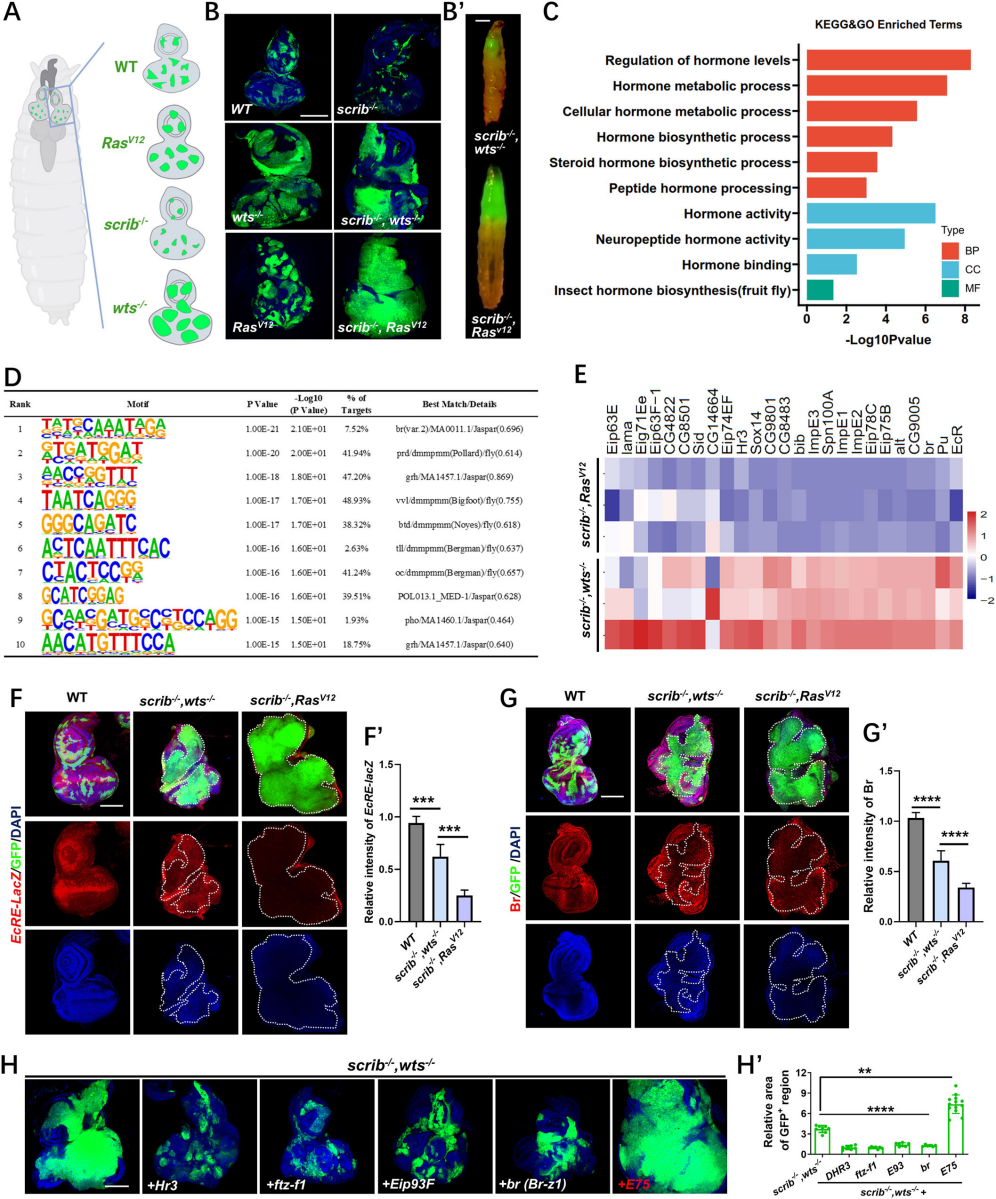
Ecdysone steroid hormone signaling is inhibited in *Drosophila* epithelial malignant tumor. (**A**) Schematic representation of eye-antennal discs from a *Drosophila* larva of *ey-Flp-*MARCM-induced mosaics. (**B**) Eye-antennal discs bearing *ey-Flp-*MARCM-induced mosaics of indicated genotypes. (**B**’) Representative images of tumor-bearing larvae of *scrib*^*−/*−^*,wts*^−*/*−^ and *scrib*^−*/−*^*,Ras*^*V12*^ on the 6th day and 9th day, respectively. (**C**) Enrichment of hormone-related terms between *scrib*^*−/*−^*,wts*^*−/−*^ and *scrib*^−*/*−^*,Ras*^*V12*^ tumors from KEGG and GO analyses. The hypergeometric distribution examination *p* < 0.1 served as the filtration threshold. Here, we displayed the terms associated with ‘hormone’ with their −log_10_ transformed *p* value. (**D**) HOMER motif analysis of ecdysone-related genes in *scrib*^*−/−*^*,wts*^−*/−*^ and *scrib*^*−/−*^*,Ras*^*V12*^ tumors. ZOOPS scoring and hypergeometric distribution test were considered collectively, and motifs with *p* < 0.0000001 were considered significantly enriched. (**E**) Heatmap profiles of ecdysone signaling-related genes in *scrib*^*−/*−^*,wts*^−*/*−^ and *scrib*^*−/*−^*,Ras*^*V12*^ tumors. (**F**, **G**) Confocal images of eye-antennal discs bearing *ey-Flp-*MARCM-induced mosaics of indicated genotype stained with anti-β-galactosidase antibody for the *EcRE-LacZ* staining (**F**) or anti-Br antibody (**G**). Quantification of the relative intensity of *EcRE-LacZ* (**F**’, from left to right, *n* = 9, 8, 8) and Br (**G**’, *n* = 5, 8, 11) in GFP positive mosaics clones. *n* represents the number of biological replicates. Statistical analysis by Ordinary one-way ANOVA test; mean ± SD. *p* values (**F**’), from left to right, ***p = 0.0005, ***p = *0*.0002*. p* values (**G**’), ****p = 0.000000916, ****p = 0.000000045. (**H**) Confocal images of eye-antennal discs bearing *ey-Flp*-MARCM-induced tumors and mosaics of indicated genotypes. (**H**’) Quantification of the relative size of GFP positive regions (*n* = 8, 7, 7, 7, 6, 13). *n* represents the number of biological replicates. Statistical analysis by ordinary one-way ANOVA test; mean ± SD. **p = 0.0019, ****p = 0.0000013, ****p = 0.00000037, ****p = 0.0000076, ****p = 0.00000079. Scale bars: 100 μm (**B**, **F**, **G**, **H**), 500 μm (**B**’). Source data are available online for this figure.

*Drosophila* tumors can be classified into two main subtypes: neoplastic and hyperplastic (Bilder et al, 2021). Neoplastic tumors exhibit signs of cell polarity loss and differentiation defects, resulting in the formation of multi-layered invasive tumors. The typical genes of this category include *scribble* (*scrib*), *discs large* (*dlg*), and *lethal giant larvae* (Greenman et al, 2007) (Bilder et al, 2000; Bilder et al, 2021). Conversely, hyperplastic tumors maintain the cell architecture of the single-layered epithelium and preserve the cell fate. Examples of these tumor-regulating genes include *warts/lats* (*wts*) and oncogenic *Ras* (*Ras*^*V12*^). We generated GFP-labeled clones harboring mutations of different tumor suppressor genes and oncogenes using the mosaic analysis with a repressible cell marker (MARCM) system (Fig. 1A) (Brumby and Richardson, 2003; Lee and Luo, 2001). In the presence of surrounding wild-type (WT) cells, the *scrib* mutant clones undergo elimination through a conserved process known as tumor-suppressive cell competition (Figs. 1A,B and EV1A) (Brumby and Richardson, 2003; Katsukawa et al, 2018; Kong et al, 2022; Sanaki et al, 2020; Zheng et al, 2023). However, when *Ras*^*V12*^ is co-expressed, the *scrib* clones are transformed into malignant tumors, and tumor-bearing animals become giant larvae on the 9th day (Fig. 1B,B’) (Brumby and Richardson, 2003; Liu et al, 2022a; Pagliarini and Xu, 2003). Interestingly, while mutation of *wts* alone resulted in larger clones, co-deletion of *wts* failed to transform *scrib* clones into large tumor masses (Figs. 1B,B’ and EV1A).

**Figure EV1:**

Ecdysone signaling inhibition promotes tumor malignancy. (**A**) Quantification of the relative size of GFP positive regions in Fig. 1B (*n* = 6, 10, 9, 5, 8, 6). *n* represents the number of biological replicates. Statistical analysis by ordinary one-way ANOVA test; mean ± SD. ***p = 0.0004, *p = 0.0378. (**B**) Volcano plot of differentially expressed genes (DEGs) in *scrib*^−*/*−^*,wts*^*−/*−^ and *scrib*^*−/−*^*,Ras*^*v12*^ tumors (top panel). Log2 fold-change of *E75* expression in *scrib*^*−/*−^*,Ras*^*v12*^ vs *scrib*^−*/−*^*,wts*^−*/−*^ tumors (bottom panel). (**C**) GSEA enrichment of ecdysone-related genes in *scrib*^−*/−*^*,wts*^−*/−*^ and *scrib*^−*/−*^*,Ras*^*v12*^ tumors. The FWER *p* < 0.05 served as the significance threshold. (**D**) Confocal images of eye-antennal discs bearing *ey-Flp-*MARCM-induced mosaics of each genotype stained with Broad (Br) antibody. (**D**’) Quantification of relative Br intensity of GFP positive mosaics clones (*n* = 6, 7, 8). *n* represents the number of biological replicates. Statistical analysis by ordinary one-way ANOVA test; mean + SD. ****p = *0*.0000000006, ***p = 0.00045. (**E**) Dorsal views of *ey-Flp-*MARCM-induced GFP-positive tumor-bearing larvae and the corresponding dissected tumor (right). Quantification of pupation rate of tumor-bearing larvae (**E**’) and relative tumor size of GFP positive mosaics clones (**E**”, *n* = 9, 4, 8, 6). *n* represents the number of biological replicates. (**E**”) Statistical analysis by ordinary one-way ANOVA test; mean ± SD. *p = 0.0222, ****p = 0.000095, **p = 0.0014. (**F**, **G**) Confocal images of eye-antennal discs bearing *ey-Flp-*MARCM-induced mosaics of each genotype. (**F**’, **G**’) Quantification of the relative size of GFP positive regions (**F**’, *n* = 8, 5, 6, 5; **G**’, *n* = 9, 9). *n* represents the number of biological replicates. (**F**’) Statistical analysis by ordinary one-way ANOVA test; mean ± SD. ns non-significant, *p* = 0.1748. (**G**’) Statistical analysis by students’ *t*-test; mean ± SD. ***p = 0.0001. (**H**) Confocal images of *ey-Flp-*MARCM-induced tumor of each genotype stained with Broad (Br) antibody. (**H**’) Quantification of relative Br intensity of GFP positive mosaics clones (*n* = 7, 5, 6, 6). *n* represents the number of biological replicates. Statistical analysis by ordinary one-way ANOVA test; mean ± SD. ****p = 0.000007, ****p = 0.0000009, ****p = 0.00000069. (**I**, **J**) Confocal images of eye-antennal discs bearing *ey-Flp-*MARCM-induced mosaics of wild-type and E75 overexpression stained with anti-β-galactosidase antibody for the *EcRE-LacZ* staining (**I**), anti-Br antibody (**J**). Clones are circled by the white dashed line. (**K**) Schematic diagram of ecdysone signaling, ecdysone response genes, and tumorigenesis according to Figs. 1H and EV1I,J. (**L**) Dorsal views of *ey-Flp*-MARCM-induced GFP-positive tumor-bearing larvae and the corresponding eye disc or tumor (right). (**L**’) Quantification of relative tumor size in (**L**) (*n* = 6, 5, 6, 6, 6, 6). *n* represents the number of biological replicates. Statistical analysis by ordinary one-way ANOVA test; mean ± SD. ****p = 0.000092, ****p = 0.000000000012. (**L**”) Quantification of larvae pupation rate in (**L**). Scale bars: 100 μm (**D**, **F**–**J**), 200 μm (**E**, **L**).

To investigate the mechanisms underlying the phenotypic differences between the *scrib*^−*/*^^*−*^*,wts*^−*/*−^ and *scrib*^*−/*^^*−*^*,Ras*^*V12*^ tumors (Fig. 1B’), we conducted bulk RNA-seq analysis. Interestingly, by analyzing the differentially expressed genes (DEGs), we noticed significant enrichment of multiple hormone-related terms (Figs. 1C and EV1B). We further performed motif enrichment analysis using HOMER (Hypergeometric Optimization of Motif EnRichment) (Heinz et al, 2010) to identify the crucial transcription factors (TFs) involved in the regulation of the DEGs. Of note, the highest-ranked TF identified was *broad* (*br*) (Fig. 1D), a known “early” ecdysone-induced gene that subsequently triggers the activation of “late” genes in the ecdysone signaling. Moreover, the gene set enrichment analysis (GSEA) and RNA-seq analysis both demonstrated a significant down-regulation of the ecdysone signaling in the *scrib*^*−/−*^*,Ras*^*V12*^ malignant tumor compared to the *scrib*^−*/−*^*,wts*^−*/−*^ benign tumors (Figs. 1E and EV1C). Consistent with these findings, the transcriptional activation of ecdysone signaling in vivo was severely impeded in malignant tumors, but only mildly inhibited in benign tumors, as demonstrated by the ecdysone response element (*EcRE*)-driven LacZ (*EcRE-LacZ*) reporter and Br staining (Fig. 1F–G’). Another malignant tumor induced by *lgl*^*−/−*^, *Ras*^*V12*^ also exhibited a significant reduction in Br (Fig. EV1D,D’). In line with the potential tumor-suppressive role of increased ecdysone signaling, we observed a robust suppression of *lgl*^*−/*−^, *Ras*^*V12*^-induced tumorigenesis and restoration of the pupation defect upon hyperactivation of ecdysone signaling through ectopic expression of three different isoforms of *EcR* (*EcRA*, *EcRB1*, and *EcRC*) (Fig. EV1E–E”), accompanied with restoration of Br (Fig. EV1H,H’). It’s worth to noting that ectopic expression of three different isoforms of *EcR* alone did not affect clonal growth (Fig. EV1F,F’). Collectively, these findings indicate that the inhibition of ecdysone signaling might play a critical role in the malignant transformation of tumors.

### E75 overexpression induces tumor malignant transformation

To further investigate the potential role of EcR signaling in regulating tumor progression, we overexpressed several well-established downstream target genes of EcR in the *scrib*^*−/−*^*, wts*^*−/−*^ tumors, including *Hr3*, *ftz-f1*, *Eip93F*, *br*, and *E75*. Surprisingly, we observed that only the overexpression of *E75* significantly enhanced the overgrowth of *scrib*^*−/*−^*,wts*^*−/−*^ tumors, while the expression of other genes impeded tumorigenesis (Fig. 1H,H’). These findings demonstrate the complexity of EcR target genes in the regulation of tumorigenesis and highlight the distinctive role of E75 in driving tumor overgrowth (Fig. EV1K). Previous studies have shown that E75 genetically represses the ecdysone-triggered cascade (Hiruma and Riddiford, 2004; Johnston et al, 2011). In line with this, *ey-Flp*-MARCM-induced clonal overexpression of the A isoform of *Eip75B* (abbreviated as *E75*) significantly inhibited the endogenous ecdysone signaling activation (Fig. EV1I,J). Moreover, the co-expression of EcR partially repressed the growth phenotype caused by E75 overexpression (Fig. EV1G,G’). Together, these findings imply that E75-induced overgrowth is partially reliant on the inhibition of EcR.

**Figure 2:**
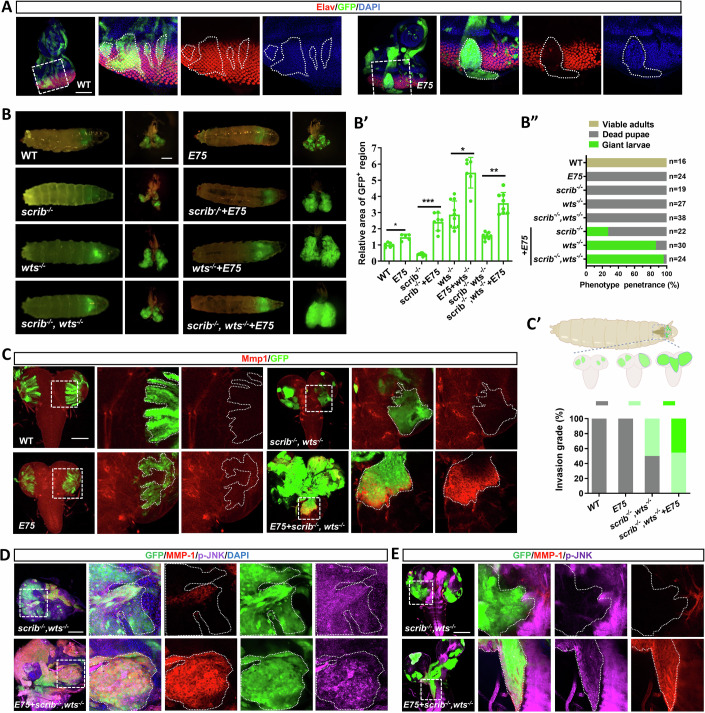
*E75* overexpression induces tumor malignant transformation. (**A**) Confocal images of eye-antennal discs bearing *ey-Flp-*MARCM-induced mosaics of wild-type and *E75* overexpression stained with anti-β-galactosidase antibody for anti-Elav antibody. Clones are circled by the white dashed line. (**B**) Dorsal views of *ey-Flp*-MARCM-induced GFP-positive tumor-bearing larvae and the corresponding eye disc or tumor (right). Quantification of relative tumor size (**B**’) (*n* = 6, 5, 9, 7, 10, 6, 8, 8) and larvae pupation rate (**B**”). Statistical analysis by ordinary one-way ANOVA test; mean ± SD. *n* represents the number of biological replicates. *p* values from left to right, *p = 0.0358, ***p = 0.0005, *p = 0.042, **p = 0.0015. (**C**) Representative confocal images of the ventral nerve cord (VNC) from *ey-Flp*-MARCM-induced tumors with indicated genotype stained with anti-Mmp1 antibody. Quantification of the invasion grade of each genotype (**E**’, bottom panel). Carton illustration of the invasion grade of VNC in tumor-bearing larvae (**C**’, top panel) (*n* = 7, 11, 8, 11). (**D**, **E**) Confocal images of eye-antennal discs bearing *ey-Flp-*MARCM-induced tumors (**D**) and ventral nerve cord (VNC) (**E**) with indicated genotype stained with anti-Mmp1 and p-JNK antibody. Scale bars: 100 μm (**A**, **C**, **D**, **E**), 200 μm (**B**). Source data are available online for this figure.

Differentiation failure is one of the key characteristics that distinguish malignant from benign tumors. Clones overexpressing *E75* exhibited a dramatic defect in differentiation, as evidenced by the staining of the neuronal differentiation marker Elav (Fig. 2A). Moreover, overexpression of *E75* exhibited a synergistic effect when combined with either *scrib*^−*/−*^ or *wts*^*−/*−^, resulting in tumor overgrowth and pupation defects (Fig. 2B–B”). Intriguingly, the overexpression of *E75* not only significantly increased the size of *scrib*^*−/−*^*,wts*^−*/−*^-induced benign tumors (Fig. 2B,B’), but also astonishingly transformed them into malignant tumors, resulting in 96.3% of the animals being unable to pupate and instead developing into giant larvae (Fig. 2B–B”). This transformation was accompanied by a significant enhancement in tumor invasive ability, as evidenced by the abundant cell autonomous Mmp1 upregulation observed at the leading edge of the invasive tumor cells (Fig. 2C,C’). Additionally, we also observed increased Mmp1 expression and JNK phosphorylation both in tumor clones and invasive tumor cells in a cell-autonomous manner (Fig. 2D,E). Furthermore, *E75* overexpression also synergistically transformed other benign tumors into malignant ones, including *Ras*^*V12*^ and *Raf*^*GOF*^*, scrib*^−*/−*^ (Fig. EV1L–L”). Taken together, these results suggest that ectopic expression of *E75* facilitates the tumor malignant transformation.

### E75 inactivates the Hippo pathway to induce tumor malignancy

**Figure 3:**
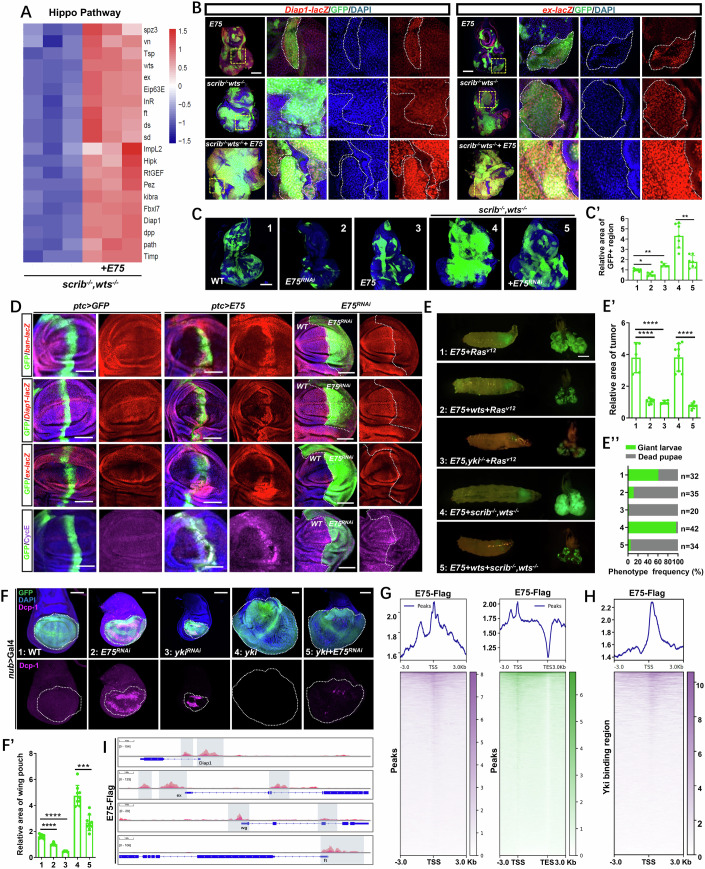
E75 inactivates the Hippo signaling pathway to induce tumor malignancy. (**A**) Heatmap profiles of the expression of Hippo signaling-related genes in *scrib*^*−/*−^*,wts*^*−/*−^ and *E75, scrib*^*−/*−^*,wts*^−*/−*^ tumors. (**B**) Confocal images of eye-antennal discs bearing *ey-Flp*-MARCM-induced mosaics of indicated genotypes stained with anti-β-galactosidase antibody for *Diap1-LacZ* and *ex-LacZ* staining. (**C**) Representative images of eye-antennal discs bearing *ey-Flp-*MARCM-induced mosaics of indicated genotypes. (**C**’) Quantification of the relative size of GFP positive regions in (**C**) (*n* = 7, 6, 5, 7, 7). *n* represents the number of biological replicates. Statistical analysis by Brown–Forsythe and Welch ANOVA tests (**C**’); mean ± SD. *p* values (**C**’), from left to right, *p = 0.014, **p = 0.0045, **p = 0.0056. (**D**) Confocal images of the wing disc with *E75* overexpression under the control of *ptc* promoter and knockdown under the control of *en or hh* promoter stained with anti-CycE or anti-β-galactosidase antibodies for *ban-LacZ*, *Diap1-LacZ*, and *ex-LacZ* staining. (**E**) Dorsal views of *ey-Flp*-MARCM-induced GFP-positive tumor-bearing larvae and the corresponding tumor. Quantification of relative tumor size (**E**’) (*n* = 5, 6, 4, 8, 6) and larvae pupation rate (**E**”). *n* represents the number of biological replicates. Statistical analysis by ordinary one-way ANOVA test (**E**’); mean ± SD. ****p = 0.0000013, ****p = 0.000005, ****p = 0.00000003. (**F**) Confocal images of wing disc with indicated genotypes stained with anti-Dcp-1 antibody. (**F**’) Quantification of the relative size of the wing pouch region of **F** (*n* = 8, 6, 5, 9, 9). *n* represents the number of biological replicates. Statistical analysis by Brown–Forsythe and Welch ANOVA tests (**F**’); mean ± SD. *p* values (**F**’), from left to right, ****p = 0.0000045, ****p = 0.00000003, ***p = 0.0001. (**G**) Binding profiles and heatmaps of E75 CUT&Tag signals are displayed within a region spanning ±3 kb around all canonical transcription start sites (TSS) (left), −3 kb around all TSS, and +3 kb around all canonical transcription end sites (TES) (right). (**H**) Line plots of the average CUT&Tag signal of E75 peaks (top panel) and heatmaps of the CUT&Tag signals of Yki in *Drosophila* (bottom panel). CUT&Tag signals are displayed within a region spanning ±3 kb around the TSS of all canonical Yki target genes genome-wide. (**I**) Browser shots of E75 CUT&Tag signal at canonical Hippo pathway target genes. The shaded areas correspond to E75 peaks. Scale bars: 100 μm (**B**, **C**, **D**, **F**) and 200 μm (**E**). Source data are available online for this figure.

To further explore the underlying mechanisms by which *E75* overexpression promotes tumor malignancy, we performed bulk RNA-seq analysis on tumors derived from *scrib*^*−/−*^*,wts*^*−/−*^ and *scrib*^−*/−*^*,wts*^−*/*−^ *+* *E75*. A total of 3457 DEGs were identified, including 1985 upregulated genes and 1472 downregulated genes (Fig. EV2A). We subjected these DEGs to further analysis using the Kyoto Encyclopedia of Genes and Genomes (KEGG) and observed a significant enrichment and inactivation of the Hippo signaling pathway (Figs. 3A and EV2B,C), a conserved pathway crucial for size control and tumorigenesis (Zheng and Pan, 2019). *Drosophila* core components of the Hippo pathway consist of Hippo (Hpo), Warts (Wts), and Yorkie (Yki). Hpo phosphorylates Wts, which subsequently phosphorylates and inactivates the transcriptional coactivator Yki (Zheng and Pan, 2019). Consistent with its inhibitory role on the Hippo signaling pathway, the ectopic expression of *E75* not only synergistically enhanced *scrib*^*−/−*^*,wts*^−*/−*^-induced upregulation of Hippo target genes, including *death-associated inhibitor of apoptosis 1* (*Diap1)-LacZ* and *expanded* (*ex*)*-LacZ* (Fig. 3B), but also led to an increase in clone size (Fig. 3C,C’). Conversely, the depletion of endogenous *E75* resulted in apoptosis (Fig. 3F) and caused a reduction in clone size, as well as adult wing and eye size (Figs. 3C,C’ and EV2D), to the same degree as observed during the activation of the Hippo signaling pathway or knockdown of *yki*. Additionally, inhibition of *E75* also suppressed tumor overgrowth induced by *scrib*^*−/−*^*,wts*^*−/−*^ (Figs. 3C,C’ and EV2D). Importantly, both overexpression and depletion of *E75* were found to modulate the expression of multiple target genes of the Hippo pathway, such as *Diap1*, *ex*, *Cyclin E* (*CycE*), and *bantam* (*ban*) (Figs. 3D and EV2G). Together, these data indicate that E75 negatively regulates Hippo signaling in *Drosophila*.

**Figure EV2:**

*E75* overexpression induces Hippo pathway-dependent growth and tumorigenesis. (**A**) MA (Minus-versus-Add) plot of *scrib*^*−/−*^*,wts*^−*/−*^ and *E75,scrib*^−*/*−^*,wts*^*−/*−^ tumors. (**B**) Enrichment analysis of signaling pathway in *scrib*^*−/−*^*,wts*^−*/*−^ and *E75,scrib*^*−/*−^*,wts*^−*/*−^ tumors. (**C**) GSEA enrichment of Hippo signaling-related genes in *scrib*^−*/−*^*,wts*^−*/*−^ and *E75, scrib*^*−/*−^*,wts*^*−/−*^ tumors. The FWER *p* < 0.05 served as the significance threshold. (**D**) Light micrographs of the adult wings (top) and eyes bearing the indicated genotypes. (**E**) KEGG enrichment of signaling pathways in *scrib*^*−/−*^*,wts*^*−/−*^ and *E75, scrib*^*−/−*^*,wts*^−*/*−^ tumors. Terms that satisfied the hypergeometric distribution were considered significant (*p* < 0.05). (**F**) Heatmap profiles of Hippo signaling-related genes in wild-type (WT) and *E75* overexpression wing discs. (**G**) Quantification of relative staining intensity of GFP positive (E75 knockdown) and negative (wild type) regions for corresponding antibodies in Fig. 3D (*n* = 6, 6 for *ban-lacZ*, *n* = 6, 6 for *ex-lacZ*, *n* = 7, 7 for *Diap1-lacZ*, *n* = 7,7 for CycE). *n* represents the number of biological replicates. Statistical analysis by paired students’ *t*-test; mean ± SD. ***p = 0.0009, ***p = 0.0009, ***p = 0.0002, ****p = 0.000007. (**H**) Confocal image of Cyclin E (CycE) antibody staining of wing discs bearing the indicated genotypes. (**H**’) Quantification of the relative size of *ptc* region of **H** (*n* = 6, 7, 4, 7, 4, 7). *n* represents the number of biological replicates. Statistical analysis by ordinary one-way ANOVA test; mean ± SD. ****p = 0.00000019, ****p = 0.0000024, ****p = 0.00000086. (**I**) Confocal images of *ey-Flp*-MARCM-induced GFP-positive mosaic clones stained with anti-β-galactosidase antibody for the *Diap1-LacZ* staining. (**J**, **K**) Dorsal views of *ey-Flp-*MARCM-induced GFP-positive tumor-bearing larvae and the corresponding dissected eye disc or tumor. Quantification of relative tumor size of GFP positive mosaics clones (**K**’, *n* = 5, 6). *n* represents the number of biological replicates. Statistical analysis by students’ *t*-test; mean ± SD. ***p = 0.004. (**L**) HOMER motif analysis of *E75* novel binding motifs in wing pouch region. ZOOPS scoring and hypergeometric distribution test were considered collectively, and motifs with *p* < 0.0000001 were considered significantly enriched. Scale bars: 100 μm (**H**, **I**), 200 μm (**A**, **D**, **J**, **K**).

In line with the observed enrichment of Hippo signaling in E75-induced malignant tumors, the Hippo pathway was also found to be enriched through a bulk RNA-seq analysis performed on *E75*-overexpressed wing discs (Fig. EV2E,F). To investigate the relationship between E75 and the Hippo pathway components, we conducted a genetic epistasis analysis. We found that overexpression of *E75* induced overgrowth, as well as the upregulation of CycE and *Diap1-lacZ* were all significantly suppressed upon the knockdown of either *yki* or *scalloped* (*sd*), or co-expression of *wts* (Fig. EV2H–I). Similarly, reducing *yki* or *sd* activity significantly blocked the tumor malignancy and pupation defect induced by E75 overexpression in both *Ras*^*V12*^ and *scrib*^*−/−*^*,wts*^*−/−*^ under pathological conditions (Figs. 3E–E” and EV2J–K’). On the other hand, the depletion of *E75* also impeded the overgrowth phenotype caused by ectopic *yki* expression (Fig. 3F,F’). The mutual genetic dependence of Yki and E75 for their respective functions implies that neither protein functions upstream nor downstream over the other. Hence, these findings collectively suggest that E75 and Yki act genetically interdependent with each other.

Given that E75 possesses a conserved DNA binding domain (DBD) and the ability to initiate gene transcription, we subsequently performed Cleavage Under Targets and Tagmentation (CUT&Tag) analysis (Kaya-Okur et al, 2019) to investigate the genome-wide DNA binding profiles of E75 in dissected wing pouch regions from *E75* overexpressed discs (Fig. 3G). In addition to the well-established binding motif, we also identified novel binding motifs of *E75* using the HOMER software (Fig. EV2L). By integrating the publicly available chromatin immunoprecipitation sequencing (ChIP-seq) data of Yki-bound chromatin (Nakahashi et al, 2013), we found that the CUT&Tag signals of E75 are significantly enriched in the region where Yki bound (Fig. 3H). Supporting this, our analysis revealed numerous E75 binding sites located within the promoter regions of canonical Hippo target genes, such as *Diap1*, *ex*, *wg*, and *fat* (*ft*) (Fig. 3I). These findings suggest that E75 may serve as a potential partner of Yki in the transcriptional regulation of its downstream genes.

### E75 activates the Notch pathway to promote tumor malignancy

**Figure 4:**
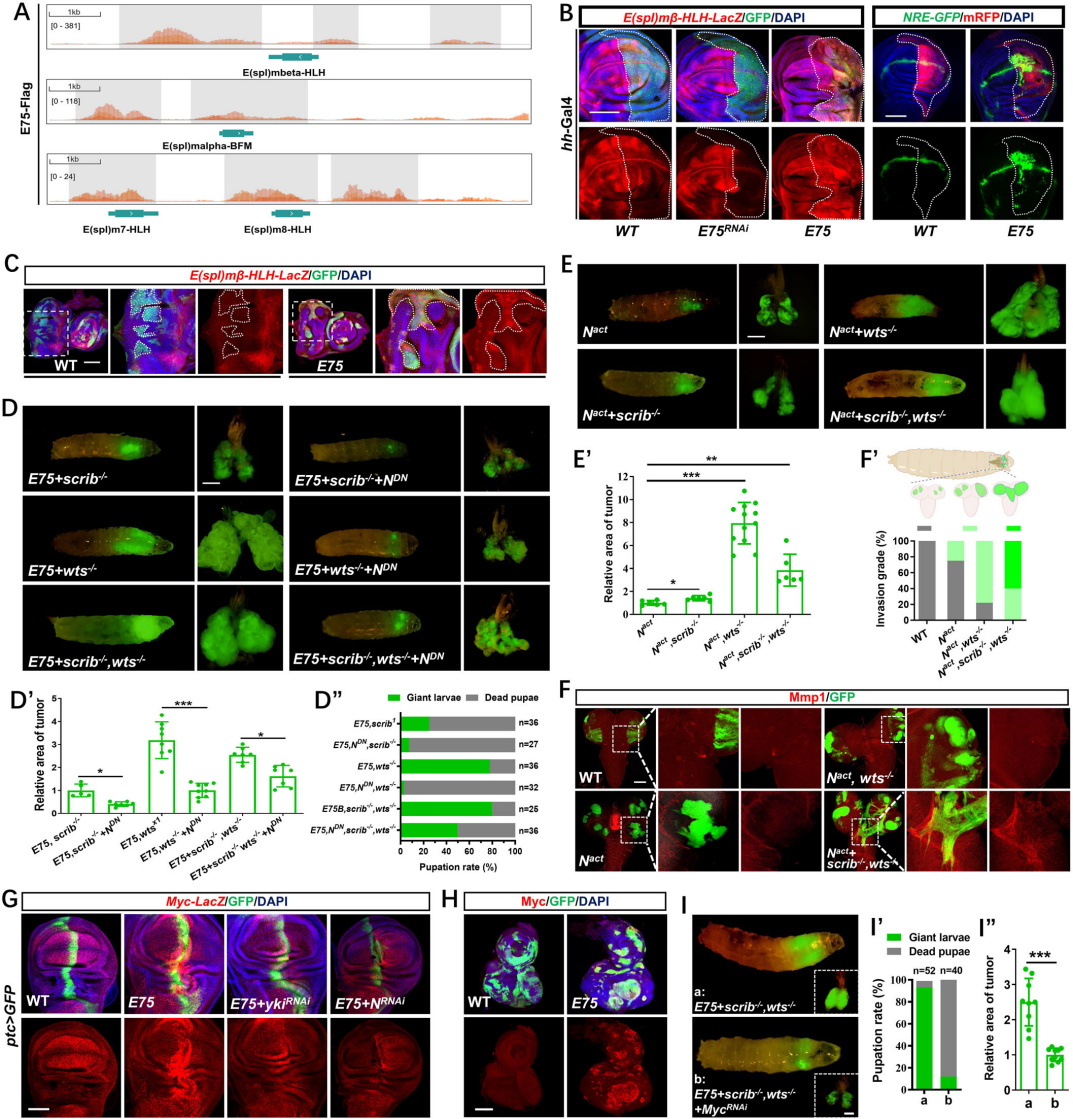
E75 activates the Notch pathway to promote tumor malignancy. (**A**) Browser shots of E75 CUT&Tag signal at canonical Notch pathway target genes. The shaded areas correspond to E75 peaks. (**B**) Confocal images of wing disc with indicated genotypes stained with anti-β-galactosidase antibody for *E(spl)mβ-HLH-LacZ* staining (left) or *NRE-GFP* expression (right). (**C**) Eye-antennal discs bearing *ey-Flp-*MARCM-induced mosaics of wild-type and *E75* overexpression stained with anti-β-galactosidase antibody for the *E(spl)mβ-HLH-LacZ* staining. (**D**, **E**) Dorsal views of *ey-Flp*-MARCM-induced GFP-positive tumor-bearing larvae and the corresponding eye disc or tumor (right). Quantification of relative tumor size (**D**’, *n* = 5, 7, 8, 9, 7, 7; **E**’, *n* = 6, 6, 12, 6), and larvae pupation rate (**D**”). *n* represents the number of biological replicates. Statistical analysis by Brown–Forsythe and Welch ANOVA tests (**D**’, **E**’); mean ± SD. *p* values (**D**’ from left to right), *p = 0.0414, ***p = 0.0005, *p = 0.0123. *p* values (**E**’ from left to right), *p = 0.041, ***p = 0.0007, **p = 0.0014. (**F**) Representative confocal images of VNC from *ey-Flp*-MARCM-induced tumors with indicated genotypes stained with anti-Mmp1 antibody. Carton illustration of invasion grade of VNC in tumor-bearing larvae (**F**’, top panel). Quantification of invasion percentage of each genotype (**F**’, bottom panel) (*n* = 12, 8, 10, 9). (**G**) Representative confocal images of wing disc bearing indicated genotypes stained with anti-β-galactosidase antibody for *Myc-LacZ* staining. (**H**) Eye-antennal discs bearing *ey-Flp-*MARCM-induced mosaics of wild-type and *E75* overexpression stained with anti-Myc antibody. (**I**) Dorsal views of *ey-Flp*-MARCM-induced GFP-positive tumor-bearing larvae and the corresponding eye disc or tumor (right corner). Quantification of larvae pupation rate (**I**’) and relative tumor size (**I**”) (*n* = 9, 10). *n* represents the number of biological replicates. Statistical analysis by Student’s *t*-test (**I**”); mean ± SD. ***p = 0.0003. Scale bars: 100 μm (**B**, **C**, **F**, **G**, **H**) and 200 μm (**D**, **E**, **I**). Source data are available online for this figure.

It is important to note that while the activation of Yki or inhibition of Hippo signaling alone can lead to overgrowth, it is insufficient to induce malignancy or the malignant transformation of *scrib*^*−/−*^ clones. This suggests that the involvement of additional signaling pathways is essential for E75-induced malignancy. Of note, in addition to *Ras*^*V12*^, *scrib*-mutated cells could also synergize with the oncogenic form of *Notch* to induce a massive overgrowth (Brumby and Richardson, 2003). Interestingly, upon re-analysis of RNA-seq data obtained from *scrib*^*−*^^*/−*^*,wts*^*−/−*^ *+* *E75* tumors, a significant enrichment of the Notch signaling was observed (Fig. EV3A,B). Furthermore, the examination of the binding regions of *Suppressor of Hairless* [*Su(H)*], the transcription factor of the Notch pathway, revealed a strong CUT&Tag signal of E75 (Fig. EV3C). Additionally, several genes known to be targeted by Notch, such as *E(spl)mβ-HLH*, *E(spl)mα-BFM*, *E(spl)m7-HLH*, and *E(spl)m8-HLH*, displayed binding signals of E75 (Fig. 4A). Consistent with these findings, the ectopic expression of *E75* upregulated the expression of several Notch target genes, including *E(spl)mβ-HLH-lacZ*, NRE-GFP, and Cut, in both physiological and E75-induced tumorigenic conditions (Figs. 4B,C and EV3D,E). Conversely, although clonal depletion of *E75* in the eye-antennal disc only partially decreased endogenous Cut expression (Fig. EV3D), inhibition of *E75* in the wing discs notably reduces endogenous Notch signaling activation, as demonstrated by *E(spl)mβ-HLH-lacZ* and Cut staining (Figs. 4B and EV3F). These results collectively imply that endogenous *E75* plays a more substantial role in regulating Notch activity in the wing discs.

**Figure EV3:**

E75 positively regulates the Notch pathway. (**A**, **B**) GSEA enrichment (**A**) and heatmap profiles (**B**) of Notch signaling-related genes in *scrib*^−*/*^^*−*^*, wts*^*−/−*^ and *E75, scrib*^−*/−*^*, wts*^*−/−*^ tumors. The FWER *p* < 0.05 served as the significance threshold. (**C**) Line plots of the average CUT&Tag signal of E75 (top panel) and the heatmaps of the CUT&Tag signals of Su(H) in *Drosophila* (bottom panel). CUT&Tag signals are displayed within a region spanning ±3 kb around all canonical transcription start sites (TSS) genome-wide. (**D**, **E**) Representative confocal images of Cut (**D**) and *E(spl)mβ-HLH*-*lacZ* (**E**) staining in *ey-Flp-*MARCM-induced tumors and clones with indicated genotype. (**F**) Confocal images of the wing disc of control or with *E75* knockdown under the control of *hh* promoter stained with anti-Cut antibody. (**G**) Dorsal views of *ey-Flp-*MARCM-induced GFP-positive clonal-bearing larvae and the corresponding dissected eye disc. Quantification of the relative size of GFP positive mosaic clones (**K**’, *n* = 6, 8). *n* represents the number of biological replicates. Statistical analysis by Student’s *t*-test; mean ± SD. ns not significant, *p* = 0.196. (**H**) Confocal images of *ey-Flp*-MARCM-induced GFP-positive mosaic clones and corresponding quantification data (**H**’, *n* = 5, 9). *n* represents the number of biological replicates. Statistical analysis by Student’s *t*-test; mean ± SD. **p = 0.0044. (**I**) Representative confocal images of *Myc-lacZ* staining in wing discs of wild-type and *E75* knockdown. (**J**) Browser shots of *E75* CUT&Tag signal at the regulatory region of *Myc*. (**K**, **L**) Representative confocal images of the wing (**K**) and eye discs (**L**) with indicated genotypes. (**K**’, **L**’) Quantification of relative GFP region of **K** and **L** (**K**’, *n* = 8, 8, 9, 9; **L**’, *n* = 6, 6, 7, 5). *n* represents the number of biological replicates. Statistical analysis by ordinary one-way ANOVA test; mean ± SD. (**K**’) ***p = 0.0004, **p = 0.0013, ***p = 0.00076. (**L**’) ****p = 0.0048, ***p = 0.0003. Scale bars: 100 μm (**D**–**F**, **H**, **I**, **K**, **L**), 200 μm (**G**).

To further investigate the in vivo role of Notch in E75-induced tumor malignancy, we inhibited Notch activation by expressing a dominant negative form of *Notch* (*N*^*DN*^). Remarkably, although inhibiting *Notch* alone in eye-antennal discs did not significantly affect clone size (Fig. EV3G,G’), inhibiting *Notch* dramatically suppressed the synergistic tumor-promoting effect caused by E75 overexpression and restored the pupation defects (Fig. 4D–D”). Conversely, genetically activating the Notch pathway by co-expressing the activated form of the Notch intracellular domain (*N*^*act*^) could transform *scrib*^−*/−*^*,wts*^*−/−*^ benign tumors into malignant ones and lead to the development of aggressive tumors (Fig. 4E–F’). Furthermore, we noticed that inhibition of *E75* also impeded N^act^-induced clonal overgrowth (Fig. EV3H,H’). It is important to note that the size of the *N*^*act*^ *+* *wts*^*−/−*^ tumor generally appeared larger than that of *N*^*act*^ *+* *scrib*^−*/*−^*,wts*^*−/−*^ tumor, possibly due to the two-dimensional expansion of tumor cells without disrupting the single-layer polarity architecture of the eye epithelium. These findings collectively suggest that the activation of the Notch pathway is both necessary and sufficient for the malignant transformation of *E75*-induced tumor progression.

### *Myc* is required for E75-induced malignant tumor transformation

The *Myc* oncogene plays a critical role in the progression of various human cancers. Intriguingly, *Myc* also serves as an evolutionally conserved transcriptional target for both the Hippo and Notch signaling pathways (Neto-Silva et al, 2010; Weng et al, 2006). Given this, we further explored whether Myc is a possible downstream target in tumor transformation induced by E75 overexpression. Depletion of *E75* decreased endogenous *Myc* transcription (Fig. EV3I), whereas ectopic expression of *E75* caused a robust increase in both transcription and protein levels of *Myc*, in a Yki- and Notch-dependent manner (Fig. 4G,H). In line with this, our CUT&Tag analysis identified multiple E75 binding sites on the promoter and gene body region of *Myc* (Fig. EV3J). Moreover, the depletion of *Myc* not only suppressed the tissue overgrowth phenotype caused by *E75* overexpression in the developing wing (Fig. EV3K–L’), but also significantly blocked E75-induced malignant transformation of *scrib*^−*/−*^*,wts*^*−/*−^ tumors (Fig. 4I–I”). These data collectively demonstrate that *Myc* functions as a downstream target gene of E75, influencing both tumor growth and malignancy.

### E75 integrates the Hippo and Notch pathways at the transcriptional factor level

**Figure 5:**
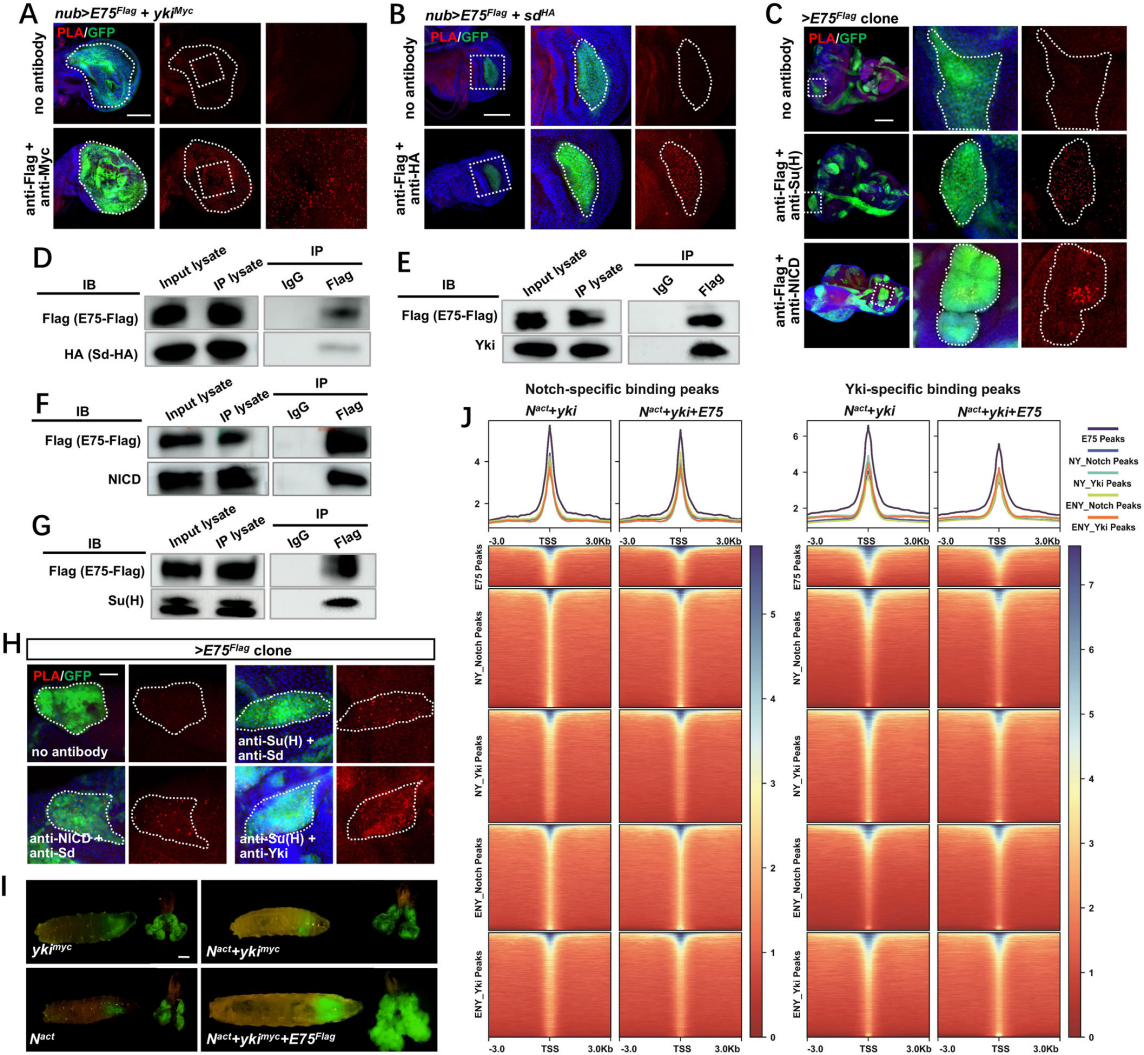
E75 integrates the Hippo and Notch pathways at the transcription factor level. (**A**, **B**) Proximity ligation assay (PLA) was performed on wing discs with indicated genotypes to test close-proximity interactions between Flag-tagged E75 and Myc-tagged Yki (**A**) or Flag-tagged E75 and HA-tagged Sd (**B**). (**C**) PLA was performed on *ey-Flp*-MARCM-induced GFP-positive clones with indicated genotypes to evaluate close-proximity interactions between E75 and Su(H) or NICD. (**D**–**G**) Co-IP assays to detect in vivo physical interactions between *E75*^*Flag*^ and *Sd*^*HA*^, NICD, Yki, and Su(H). Lysates from *ey-Flp-*MARCM-induced epithelial tumors with indicated genotypes (**D** and **E**: ey-Flp1/+; act>y^+^>GAL4, UAS-GFP/UAS-Sd^HA^, UAS-E75^Flag^; Tub-Gal80, FRT82B/FRT82B, UAS-Yki; **F** and **G**: ey-Flp1/+; act>y^+^>GAL4, UAS-GFP/UAS-Notch^act^, UAS-E75^Flag^; Tub-Gal80, FRT82B/FRT82B, UAS-Yki) were immunoprecipitated and probed with the indicated antibodies. (**H**) PLA was performed on *ey-Flp-*MARCM-induced *E75*^*Flag*^ overexpression clones to test close-proximity interactions between endogenous NICD and Sd, Su(H) and Sd, Su(H), and Yki. (**I**) Representative dorsal views of ey-Flp-MARCM-induced GFP-positive tumor-bearing larvae and the corresponding dissected tumor (right). (**J**) Line plots (top panels) show the average CUT&Tag signal of Notch-specific peaks of *N*^*act*^ *+* *Yki* and *E75* *+* *N*^*act*^ *+* *Yki*, and Yki-specific peaks of *N*^*act*^ *+* *Yki* and *E75* *+* *N*^*act*^*+Yki*. Clustered heatmaps (bottom panels) show the Notch-specific peaks of *N*^*act*^ *+* *Yki* and *E75* *+* *N*^*act*^ *+* *Yki* (left) and Yki-specific peaks of *N*^*act*^ *+* *Yki* and *E75* *+* *N*^*act*^ *+* *Yki* (right). CUT&Tag signals are displayed within a region spanning ±3 kb around all canonical TSS. E for E75, N for Notch, and Y for Yki. Scale bars: 20 um (**G**), 100 μm (**A**–**C**), and 200 um (**I**). Source data are available online for this figure.

**Figure EV4:**
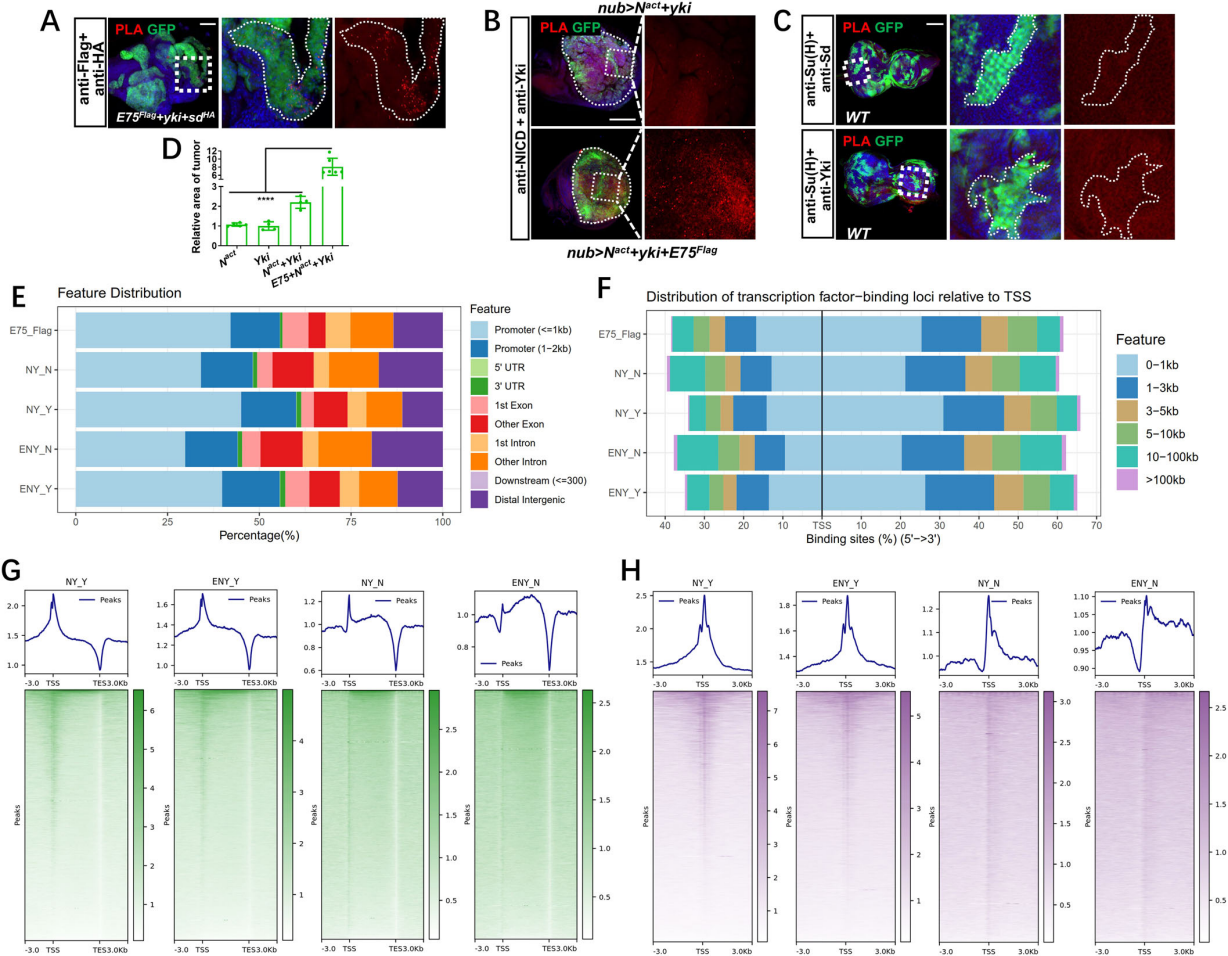
E75 regulates the transcription of the Hippo and Notch pathway target genes. (**A**) PLA was performed on eye discs bearing *ey-Flp-*MARCM-induced *E75*^*Flag*^, *Yki*, and *Sd*^*HA*^ co-expressed clones to test close-proximity interactions between E75 and Sd. (**B**) PLA was performed on wing discs with *Yki* and *Sd*^*HA*^ co-expression under the control of *nub* promoter, with or without *E75*^*Flag*^ expression, to test close-proximity interactions between NICD and Yki. (**C**) PLA was performed on eye discs bearing *ey-Flp-*MARCM-induced WT clones to test close-proximity interactions between Su(H) and Sd, as well as Su(H) and Yki. (**D**) Quantification of relative tumor size of GFP-positive tumor clones in Fig. 5I (*n* = 5, 4, 4, 6). *n* represents the number of biological replicates. Statistical analysis by ordinary one-way ANOVA test; mean ± SD. ****p = 0.0000006, ****p = 0.0000011, ****p = 0.0000112. Error bars from left to right, 0.075, 0.18, 0.26, and 2.54. (**E**) Feature distribution of genomic annotations of CUT&Tag in E75 peaks (E75_Flag), NICD peaks of *N*^*act*^ and *Yki* overexpression (NY_N), Yki peaks of *N*^*act*^ and *Yki* overexpression (NY_Y). (**F**) Distribution of binding loci relative to TSS of peaks mentioned in (**D**). (**G**) Binding profiles and heatmaps of NICD and Yki in *N*^*act*^*,Yki* and *E75, N*^*act*^*,Yki* tumors. CUT&Tag signals are displayed within a region spanning −3 kb around all canonical TSS and +3 kb around all canonical transcription end sites (TES). (**H**) Binding profiles and heatmaps of NICD and Yki in *N*^*act*^*,Yki* and *E75, N*^*act*^*,Yki* tumors. CUT&Tag signals are displayed within a region spanning −3 kb and +3 kb around all canonical TSS. Scale bars: 100 μm (**A–C**).

The observed similarity in binding sites between E75 and Yki or Su(H), as demonstrated by our E75 CUT&Tag analysis, indicates a potential physical interaction between E75 and the transcriptional complexes associated with the Hippo and Notch pathways. To test this, we performed a proximity ligation assay (PLA) in the *Drosophila* wing and eye epithelium to detect the protein-protein interactions at the subcellular level in situ (Fredriksson et al, 2002). Compared to the negative controls, we detected robust positive PLA signals between Flag-E75 and both Myc-tagged Yki and HA-tagged Sd (Fig. 5A,B). The interactions between E75 and Sd were also seen in eye-disc clones expressing *E75*^*Flag*^, *yki*, and *Sd*^*HA*^ (Fig. EV4A). Additionally, PLA revealed strong interactions between overexpressed E75 and the endogenous NICD and Su(H) (Fig. 5C). To confirm these physical interactions, we performed co-immunoprecipitation (co-IP) assays with E75 and the mentioned transcription factors. Consistently, when *E75* was ectopically expressed with *Yki* and *Sd* in the developing eye epithelium, robust physical interactions were detected between Flag-tagged E75 and Yki or HA-tagged Sd (Fig. 5D,E). Similarly, both endogenous NICD and Su(H) could form a complex with Flag-tagged E75 in tumor clones that co-expressed *E75*, *NICD*, and *Yki* (Fig. 5F,G). Together, these data suggest that E75 can physically bind with the downstream transcriptional complexes of both Hippo and Notch pathways.

Under tumorigenic stress, NICD can form a complex with a transcriptional coactivator with PDZ-binding motif (TAZ), the human ortholog of Yki (Kim et al, 2017). Indeed, we noticed that discs expressing both *yki* and *N*^*act*^ showed a detectable but moderate PLA signal (Fig. EV4B). Interestingly, it is noteworthy that no PLA signals were detected between Su(H) and Yki or Sd under physiological conditions (Fig. EV4C). However, upon the overexpression of *E75*, a dramatic increase in PLA signals was observed between Su(H) and Yki or Sd, as well as NICD and Sd (Figs. 5H and EV4B). This observation suggests that ectopic expression of *E75* can enhance the physical association between downstream TFs of the Hippo and Notch pathways. Consistent with this notion, ectopic expression of *E75* synergistically enhanced the tumorigenic potential of the benign tumor induced by the co-expression of *N*^*act*^ and *yki*, leading to their transformation into malignant tumors (Figs. 5I and EV4D).

To further investigate the effect of E75 on the TF interacting network and chromatin landscape, we conducted NICD and Yki CUT&Tag analyses on tumor samples from *N*^*act*^ + *yki* expressing (Weghorn and Sunyaev, 2017) and *E75* *+* *N*^*act*^ *+* *yki* expressing (ENY) eye epithelium. The peaks were annotated based on the genomic locations (Fig. EV4E) and relative distance to transcriptional start sites (TSSs) (Fig. EV4F). For the NY tumors, a total of 6825 NICD-annotated and 7895 Yki-annotated peaks were identified, while for the ENY tumors, 5758 NICD-annotated and 6756 Yki-annotated peaks were uncovered. To visualize the levels and distribution of NICD and Yki, we generated spike-normalized coverage heatmaps at a genome-wide scale (Fig. EV4G). Interestingly, we observed a general decrease in signal intensities of both NICD and Yki around the TSSs when *E75* was co-expressed (Fig. EV4H). Next, we plotted the NICD and Yki CUT&Tag signals around the TSS of protein-coding genes where E75 bound. We found that while both NICD and Yki bind to the regions accessible to E75, their signal intensities were also reduced upon *E75* expression (Fig. 5J).

Taken together, these results suggest that E75 effectively integrates the Hippo and Notch pathways at the transcriptional level. Ectopically expressed E75 not only physically associates with the TFs of both pathways, but also facilitates its binding to novel chromatin regions. Both actions collectively contribute to tumor malignancy.

### Silencing *NR1D2* suppressed glioblastoma stem cell-driven tumor growth

**Figure EV5:**
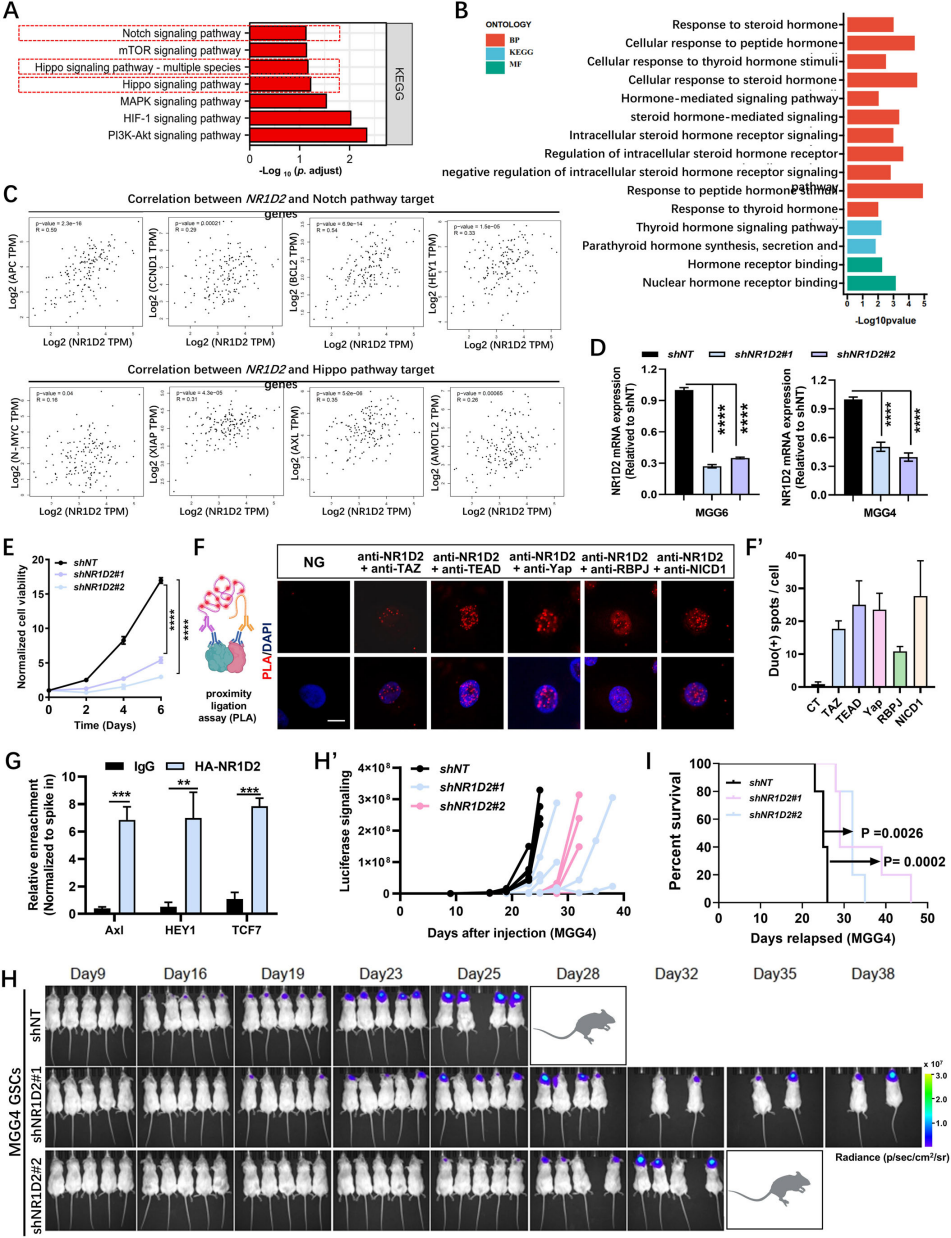
Silencing *NR1D2* suppressed glioblastoma stem cell-driven tumor growth. (**A**) KEGG analysis indicated enrichment of Hippo and Notch signaling in *NR1D2*-depleted U87 MG cells. Terms satisfying the hypergeometric distribution were considered significant (*p* < 0.05). (**B**) KEGG and GO analyses indicated the enrichment of multiple terms associated with hormone regulation in *NR1D2*-depleted U87 cells. Terms satisfying the hypergeometric distribution were considered significant (*p* < 0.05). (**C**) Pair-wise gene correlation analysis of *NR1D2* and target genes of Notch pathway (top panels) and Hippo pathway (bottom panels) in GBM by GEPIA2. (**D**) Relative mRNA expression of *NR1D2* in GSCs and *NR1D2*-depleted cells. *n* = 3 for each sample. *n* represents the number of technical replicates. Statistical analysis by two-tailed Student’s *t*-tests; mean + SD. ****p = 0.0000000022, ****p = 0.00000001, ****p = 0.000011, ****p = 0.0000033. (**E**) Relative cell viability of MGG4 of shNT and *shNR1D2*. *n* = 3 for each sample. *n* represents the number of technical replicates. Statistical analysis by two-way repeated measures ANOVA with Dunnett’s multiple hypothesis test correction; mean ± SD. ****p = 0.0000039, ****p = 0. 0.000006. (**F**) PLA working model (left). PLA analysis in U87 MG cells to test close-proximity interactions between NR1D2 and TAZ, TEAD, Yap, RBPJ, and NICD1 (right). (**F**’) Quantification of PLA signal intensity in (**E**) (*n* = 7, 6, 9, 6, 6, 6); mean + SD. *n* represents the number of biological replicates. (**G**) CUT&Tag qPCR analysis of *Axl*, *HEY1*, and *TCF7* in U87 MG. U87 cells transfected with *HA-NR1D2* were used for HA enrichment quantification on promoter region (−500 to 0). *n* = 3 for each sample. *n* represents the number of technical replicates. Statistical analysis by two-tailed Student’s *t-* tests; mean + SD. ***p = 0.0003, **p = 0.0042, ***p = 0.0001. (**H**) In vivo bioluminescence imaging of NSG mice bearing tumors on the 9th, 16th, 19th, 23rd, 25th, 28th, 32nd, 35th, and 38th day after MGG4 GSCs were injected into the mice brains. The injected MGG4 GSCs were transfected with *shNT*, *shNR1D2#1*, or *shNR1D2#2*, respectively. (**H**’) Quantification of tumor size by in vivo luciferase assays. (**I**) Survival curve of NSG mice bearing intracranial tumors from MGG4 GSCs transfected with *shNT*, *shNR1D2#1*, or *shNR1D2#2*, respectively. Statistical analysis by log-rank (Mantel-Cox) test. **p = 0.0026, **p = 0.0002. Scale bar: 10 μm (**F**).

Next, we investigated whether E75 plays a conserved role in regulating Notch and Hippo pathway-mediated tumorigenesis in mammals. *NR1D2* (*nuclear receptor subfamily 1 group D member 2*) encodes the mammalian ortholog of *E75* and belongs to the nuclear hormone receptor family. We have previously demonstrated that depleting *NR1D2* impedes the cell mobility and viability of glioblastoma (GBM) - a highly aggressive and fatal brain tumor (Yu et al, 2018). Interestingly, out of the 3760 DEGs identified in *NR1D2*-depleted GBM cells, both the Notch and Hippo signaling pathways were enriched through KEGG and GO analyses (Fig. EV5A). Furthermore, the functional annotation analysis of the DEGs revealed an enrichment of 15 hormone-related terms (Fig. EV5B). This set of 201 DEGs consisted of 69 upregulated genes and 132 downregulated genes. Notably, the knock-down of *NR1D2* significantly affected two major hormone categories: steroid hormones and thyroid hormones (Fig. EV5B). Moreover, analysis utilizing GEPIA (Gene Expression Profiling Interactive Analysis) indicated that several target genes of both pathways positively correlated with elevated *NR1D2* expression in GBM (Fig. EV5C).

**Figure 6:**
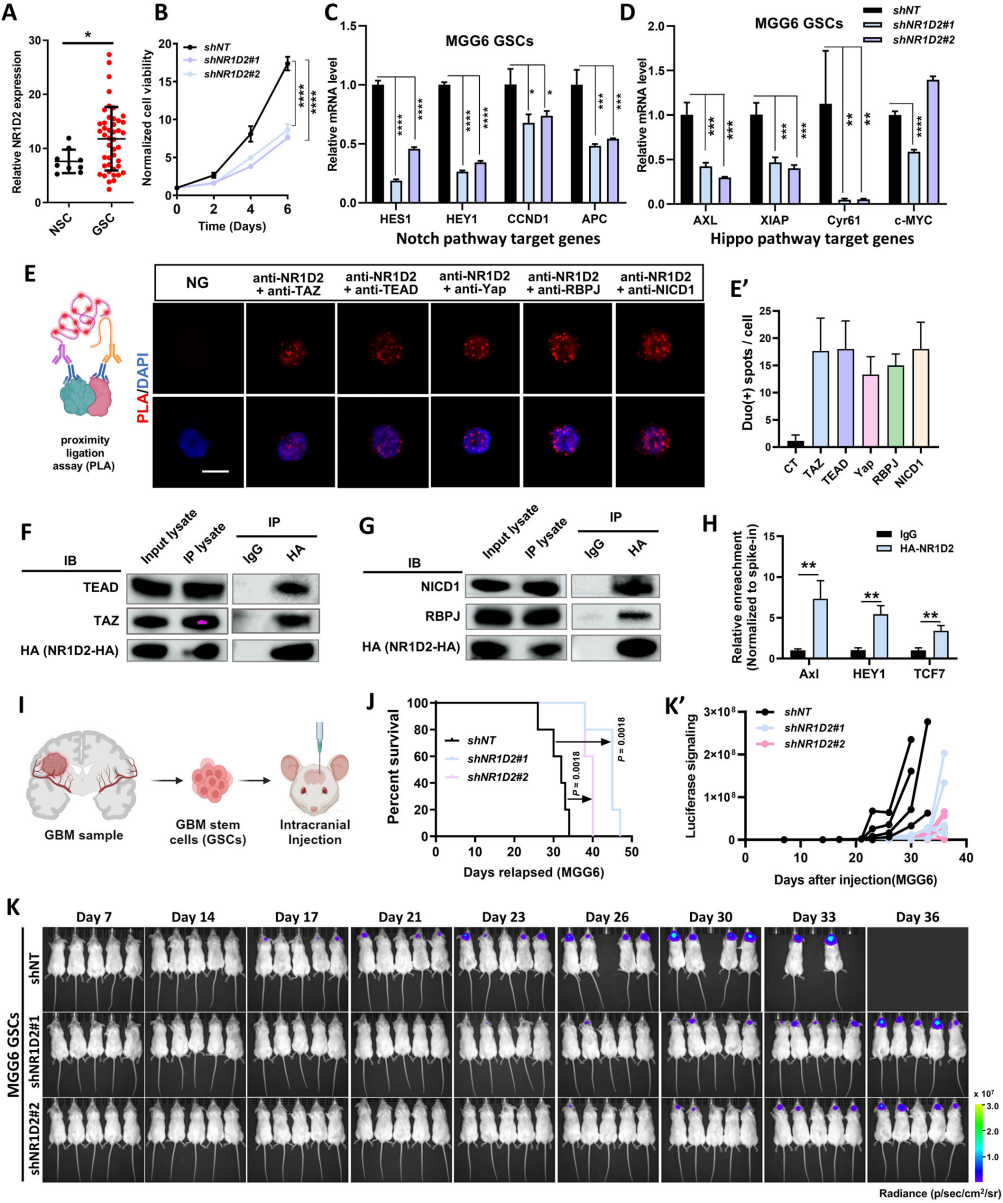
Silencing *NR1D2* suppressed glioblastoma stem cell-driven tumor growth. (**A**) Relative expression of *NR1D2* in neural stem cell (NSC) and glioblastoma stem cell (GSC). Statistical analysis by unpaired Student’s *t*-tests (**A**); mean ± SD. *p = 0.0418. (**B**) Relative cell viability of MGG6 of shNT and *shNR1D2*. Statistical analysis by Two-way repeated measures ANOVA with Dunnett’s multiple hypothesis test correction; mean ± SD. ****p = 0.0000068, ****p = 0.00000206. (**C**, **D**) Relative mRNA level of Notch target genes (*HES1*, *HEY1*, *CCND1*, and *APC*) (**C**) and Hippo target genes (*Axl*, *XIAP*, *Cyr61*, and *Myc*) (**D**). Statistical analysis by Ordinary one-way ANOVA tests (**C**, **D**); mean + SD. *p* values in (**C**) from left to right, ****p = 0.00000002, ****p = 0.00000047, ****p = 0.00000001, ****p = 0.000000003, *p = 0.0101, *p = 0.0249, ***p = 0.0004, ***p = 0.0008. *p* values in (**D**) from left to right, ***p = 0.0003, ***p = 0.0001, ***p = 0.0007, ***p = 0.0004, **p = 0.0061, **p = 0.006, ****p = 0.00000048. (**E**) PLA was performed in MGG6 GSCs to test close-proximity interactions between NR1D2 and TAZ, TEAD, Yap, RBPJ, and NICD1. (**E**’) Quantification of PLA signal intensity in (**E**) (*n* = 7, 6, 6, 6, 6, 7); mean + SD. (**F**, **G**) Co-IP assays to detect physical interactions between NR1D2-HA and endogenous TEAD, TAZ, NICD1, and RBPJ in U87 MG cells. Lysates from U87 MG cells with stably transfected NR1D2-HA were immunoprecipitated (IP) and probed with the indicated antibodies. (**H**) CUT&Tag qPCR analysis of *Axl*, *HEY1*, and *TCF7* in MGG6. MGG6 cells transfected with *HA-NR1D2* were used for HA enrichment quantification on promoter region (−500 to 0). *n* = 3 for each sample. *n* represents the number of technical replicates. Statistical analysis by two-tailed Student’s *t*-tests; mean + SD. *p* values, from left to right, **p = 0.0078, **p = 0.0027, **p = 0.0043. (**I**) Schematic diagram of GSCs brain injection. (**J**) Survival curve of NSG mice bearing intracranial tumors from MGG6 GSCs transfected with *shNT, shNR1D2#1*, or *shNR1D2#2*, respectively. Statistical analysis by Log-rank (Mantel-Cox) test, **p = 0.0018 (*shNT* vs. *shNR1D2#1*), **p = 0.0018 (*shNT* vs. *shNR1D2#2*). (**K**) In vivo bioluminescence imaging of NSG mice bearing tumors on the 7th, 14th, 17th, 21st, 23rd, 26th, 30th, 33rd, and 36th day after MGG6 GSCs were injected into the mice brains. The injected MGG6 GSCs were transfected with *shNT*, *shNR1D2#1*, or *shNR1D2#2* respectively. (**K**’) Quantification of tumor size by in vivo luciferase assays. Scale bar: 10 μm (**E**). Source data are available online for this figure.

GBM exhibits intra-tumoral heterogeneity with self-renewing glioblastoma stem cells (GSCs) at the hierarchical apex, and GSCs are functionally characterized by their capacities for self-renewal and tumor initiation, along with their additional roles in tumor angiogenesis, radio-resistance, and chemoresistance (Bao et al, 2006; Gimple et al, 2022). Notably, we found the expression level of *NR1D2* was significantly upregulated in GSCs compared to neural stem cells (NSCs) (Fig. 6A) (Mack et al, 2019). Furthermore, the depletion of *NR1D2* using two non-overlapping short hairpin RNAs (shRNA) (Fig. EV5D) strongly decreased cell proliferation of two patient-derived GSCs (MGG6 and MGG4) (Figs. 6B and EV5E) and led to a significant decrease in the expression levels of Notch and Hippo pathway target genes in GSCs (Fig. 6C,D). Similar to the interacting network of TFs identified in *Drosophila*, we found that NR1D2 also physically interacts with TFs of both the Hippo and Notch pathways in GSCs and GBM cells, as demonstrated by PLA and co-IP assays (Figs. 6E–G and EV5F,F’). To verify whether NR1D2 could directly bind to the promoter regions of Hippo and Notch target genes, we performed chromatin immunoprecipitation (ChIP) assays in GSCs and GBM cells that stably expressed HA-tagged NR1D2 (HA-NR1D2). Our data showed that NR1D2 binds to the promoter regions of multiple Hippo and Notch target genes, including *Axl*, *HEY1*, and *TCF7* (Figs. 6H and EV5G). Finally, we examined the impact of disrupting *NR1D2* on GSC-driven tumor growth in vivo. Luciferase-expressing GSCs, transfected with lentivirus expressing *NR1D2* shRNA or non-targeting control shRNA (shNT), were injected into the right cerebral cortex of NSG mouse brains (Fig. 6I). Bioluminescent imaging showed that *NR1D2* knockdown markedly suppressed GSC-driven tumor growth and extended the survival of mice compared with controls (Figs. 6J–K’ and EV5H–I). Collectively, these results indicate that NR1D2 plays a conserved role in regulating the Hippo and Notch pathways and is crucial for GSC-induced tumor growth in vivo.

## Discussion

**Figure 7:**
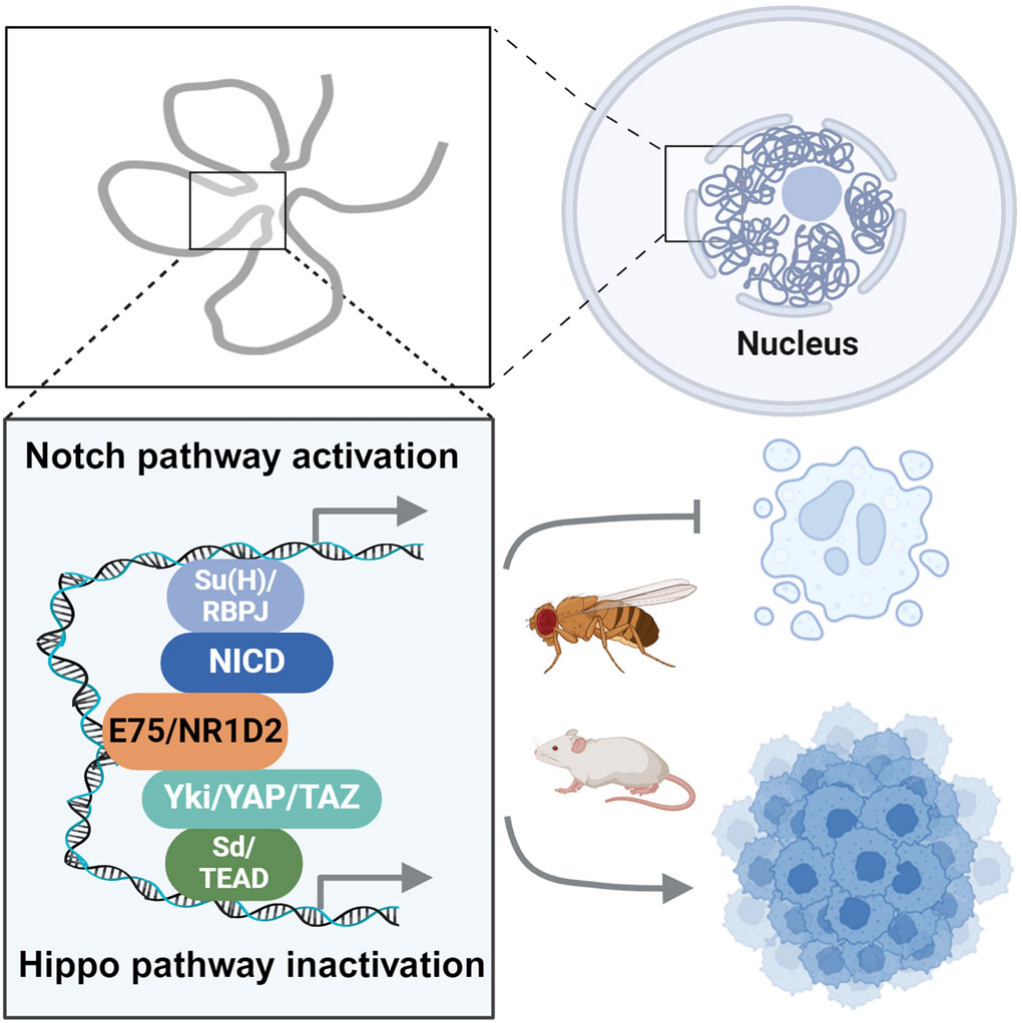
Schematic of E75/NR1D2 induced tumor malignancy in *Drosophila* and mammals. This model illustrates the molecular mechanisms by which E75 and NR1D2 contribute to tumorigenesis. E75 overexpression supports tumor growth, and its suppression triggers cell death. E75 modulates both Hippo and Notch pathways by binding with their transcriptional effectors—Sd, Yki, NICD, and Su(H) in *Drosophila*; TEAD, YAP/TAZ, RBPJ, and NICD in mammals—at a transcriptional and chromatin level. This interaction activates oncogenic genes within these pathways, promoting the malignant transformation of tumors.

Hormone therapies are commonly employed to inhibit the proliferation of hormone-dependent cancers, such as breast and prostate cancers. Nonetheless, as time passes, cancer cells may develop mechanisms to survive, even in the absence of hormones or in the face of hormone blockers, resulting in resistance to hormone therapy (Hanker et al, 2020; Metcalfe et al, 2018). However, the specific mechanisms responsible for tumor progression and malignancy in hormone signaling-reduced tumors remain predominantly uninvestigated. Here we identified the primary hormone-responsive gene *E75* as a crucial oncogene in promoting tumor malignant transformation in *Drosophila*. We found that the inhibition of ecdysone signaling is a key characteristic of *Drosophila* malignant tumors. By gain-of-function analysis of ecdysone-induced genes, we found ectopic expression of *E75* could feedback inhibit ecdysone signaling and facilitate the tumor malignancy of benign tumors by integrating two essential tumor-regulating pathways, namely the Hippo and Notch pathways (Fig. 7).

The nuclear receptor E75 has been implicated in the regulation of various biological processes, including axon degeneration and regrowth (Rabinovich et al, 2016), circadian clock regulation (Jaumouillé et al, 2015), and cell migration (Wang et al, 2020). Recent studies have shown that ecdysone, released from the adult ovary following mating, exerts a remote effect on intestinal cell differentiation and proliferation through the downstream response gene *E75* (Ahmed et al, 2020; Zipper et al, 2020). In this study, we reveal a novel growth and tumor-regulating role of E75 in *Drosophila* epithelium cells. Under physiological conditions, depletion of *E75* induces apoptosis and leads to size reduction, while *E75* overexpression promotes overgrowth. This is in line with a recent study showing that low titer ecdysone signaling activation promotes cell proliferation (Gavish et al, 2023). Under pathological conditions, we found that *E75* overexpression induces tumor malignant transformation in diverse tumor models. Moreover, consistently with the growth suppression role of ecdysone activation, we also show that hyperactivation of different EcR isoforms effectively impedes *lgl*^*−/*−^*,Ras*^*V12*^-induced tumor malignancy.

The physical associations observed between E75, and the downstream transcription complexes of the Hippo and Notch pathways indicate that E75 potentially functions as an integrator, bridging these two signaling networks. We believe that this E75-mediated integration enables cells to generate coordinated responses that are essential for various physiological and pathological processes. Through the integration of CUT&Tag with RNA-seq analyses, we found that both NICD and Yki bind to the regions accessible to E75. Notably, E75 also specifically binds and induces the expression of numerous additional genes, indicating its potential role as an integrator in coordinating the activity of multiple signaling pathways. Further investigations are required to gain a comprehensive understanding of the underlying mechanisms involved.

Our data indicates that both *E75* and *NR1D2* should be considered oncogenes conceptually. The expression of *E75* promotes tumor growth, and so does *NR1D2*. *E75*, being an ecdysone-induced gene, is downregulated in *Drosophila* malignant tumors due to the reduction of ecdysone signaling. Currently, our understanding of why ecdysone signaling is downregulated in fly malignant tumors is limited. However, it would be an oversimplification to conclude that the expression of every oncogene must be upregulated in tumors. Similarly, multiple target genes of Yki/YAP act as upstream regulators of the Hippo pathway and should be classified as tumor suppressors. Nevertheless, their levels are still upregulated when Yki/YAP is activated, which is a common phenomenon observed in various tumors.

Previous studies have investigated the function of NR1D2, also known as REV-ERBβ, the mammalian homolog of E75, in GBM and melanocytic navy. However, these studies have reported inconsistencies of NR1D2 in the regulation of tumor progression (Sulli et al, 2018; Yu et al, 2018). Additionally, it is noteworthy that many studies employed NR1D2 agonists to examine its function, but this approach may have induced NR1D2-independent effects on cell proliferation (Dierickx et al, 2019; Xu et al, 2022). Consequently, the validation of *NR1D2* in vivo loss-of-function becomes essential for a better understanding of its potential as a therapeutic target and its association with tumor progression. Consistent with the elevation of *NR1D2* expression in the GSCs, we found that the depletion of *NR1D2* inhibits GSC proliferation and improves the survival of tumor-bearing mice. This indicates that *NR1D2* functions as an oncogene in promoting GBM progression in vivo. Moreover, we show that NR1D2, similar to E75, physically interacts with transcription factors of both Hippo and Notch pathways, regulating the expression of their target genes. This suggests that E75/NR1D2 has an evolutionarily conserved function in tumorigenesis. While hormone therapy is not conventionally considered as a standard treatment for GBM, recent studies have presented its potential application in targeting nuclear hormone receptors for GBM treatment (Gonzalez-Mora and Garcia-Lopez, 2021; Rodríguez-Lozano et al, 2019). Further investigation of the in vivo functions of *NR1D2* using knock-out mice across various tumor types could be particularly insightful and advance the discovery of novel therapeutic strategies for both hormone-dependent and hormone-independent tumors.

## Methods

**Table:** Reagents and tools table

Reagent or Resource	Source	Identifier
**Experimental models:** ***Drosophila melanogaster***		
w^1118^	Bloomington Drosophila Stock Center	Cat# 5905
UAS-E75.RNAi (II)	Vienna Drosophila Resource Center	Cat# v44851
UAS-E75.RNAi (III)	Bloomington Drosophila Stock Center	Cat# 26717
ptc-Gal4	Gift from Lei Xue (Tongji University)	FBal0287777
hh-Gal4	Gift from Lei Xue (Tongji University)	FBal0121962
en-Gal4	Bloomington Drosophila Stock Center	Cat# 99568
nub-GAL4	Bloomington Drosophila Stock Center	Cat# 86108
UAS-E75^Flag^	Gift from Jiong Chen (Nanjing University)	Original source: (Rabinovich et al, 2016)
mir-ban^L1170a^ (ban-LacZ)	Bloomington Drosophila Stock Center	Cat# 10154
ex^e1^ (ex-lacZ)	Bloomington Drosophila Stock Center	Cat# 44249
myc-LacZ^G0354^/FM7c	Bloomington Drosophila Stock Center	Cat# 11981
E(spl)-HLH-mβ-lacZ	Gift from Hai Huang (Zhejiang University)	N/A
NRE-GFP	Gift from Hai Huang (Zhejiang University)	N/A
yki^B5^	Gift from Duojia Pan (University of Texas Southwestern Medical Center)	(Huang et al, 2005) FBal0194179
UAS-yki^Myc^ (II)	Gift from Lei Xue (Tongji University)	N/A
UAS-yki (III)	Gift from Duojia Pan (University of Texas Southwestern Medical Center)	(Huang et al, 2005)
UAS-sd.RNAi	Gift from Lei Zhang (Chinese Academy of Sciences)	(Zhang et al, 2008a)
UAS-Hr3	Fly ORF	Cat# F000034
UAS-ftz-f1	Bloomington Drosophila Stock Center	Cat# 64290
UAS-Eip93F	Fly ORF (Gift from Sheng Li lab)	Cat# F000587
UAS-Br-Z1	Bloomington Drosophila Stock Center	Cat# 51190
UAS-Sd^HA^	Gift from Lei Zhang (Chinese Academy of Sciences)	N/A
UAS-wts	Gift from Shian Wu (Nankai University)	N/A
UAS-Notch.RNAi	Vienna Drosophila Resource Center	Cat# v1112
UAS-myc.RNAi	Vienna Drosophila Resource Center	Cat# v2947
UAS-Notch^act^	Gift from Hai Huang (Zhejiang University)	N/A
UAS-Notch^DN^	Gift from Hai Huang (Zhejiang University)	N/A
UAS-Yki.RNAi	Vienna Drosophila Resource Center	Cat# v40497
EcRE-lacZ	Bloomington Drosophila Stock Center	Cat# 4516
EcRE-lacZ	Bloomington Drosophila Stock Center	Cat# 4517
UAS-Ras^V12^, FRT82B, scrib^1^/TM6B Tb	Gift from Tian Xu (Westlake University)	N/A
UAS-Ras^V12^	Previously described	(Ma et al, 2017)
UAS-GFP	Gift from Lei Xue (Tongji University)	N/A
GMR-GAL4 (II)	Gift from Lei Xue (Tongji University)	N/A
UAS-EcRA	Bloomington Drosophila Stock Center	Cat# 6470
UAS-EcRB1	Bloomington Drosophila Stock Center	Cat# 6469
UAS-EcRC	Bloomington Drosophila Stock Center	Cat# 6868
lgl^4^, FRT40A, UAS-Ras^V12^/Cyo	Gift from Tian Xu (Westlake University)	N/A
UAS-Raf^GOF^, FRT82B, srcib^1^/ TM6B Tb	Gift from Tian Xu (Westlake University)	N/A
FRT40A	Gift from Tian Xu (Westlake University)	N/A
FRT82B	Gift from Tian Xu (Westlake University)	N/A
scrib^1^	Gift from Tian Xu (Westlake University)	Original source: (Bilder et al, 2000)
wts^x1^	Gift from Tian Xu (Westlake University)	(Xu et al, 1995)
yw, ey-Flp1; act>y^+^>GAL4, UAS-GFP; FRT82B, Tub-Gal80, (82B Tester)	Gift from Tian Xu (Westlake University)	N/A
yw, ey-Flp;Tub-Gal80, FRT40A; act>y^+^>Gal4, UAS-GFP	Gift from Tian Xu (Westlake University)	N/A
**Experimental models: Mouse**		
NOD.Cg-*Prkdc*^*scid*^*Il2rg*^*em1Smoc*^	Shanghai Model Organisms Center, Inc.	Cat# NM-NSG-001
**Experimental models: Antibodies**		
Mouse monoclonal anti-Broad	Developmental Studies Hybridoma Bank	Cat# 25E9 AB_3086638
Mouse monoclonal anti-β-gal	Developmental Studies Hybridoma Bank	Cat# 40-1a; RRID: AB_2314509
Rat anti-Elav	Developmental Studies Hybridoma Bank	Cat# 7E8A10 AB_3086637
Mouse monoclonal anti-Mmp1	Developmental Studies Hybridoma Bank	Cat# 3A6B4; RRID: AB_579780, Cat# 3B8D12; RRID: AB_579781, Cat# 5H7B11; RRID: AB_579779
Rabbit polyclonal anti-Cleaved *Drosophila* Dcp-1	Cell Signaling Technology	Cat# 9578; RRID: AB_2721060
Rabbit polyclonal anti-active JNK	Promega	Cat# v2973; RRID: AB_3391716
Rabbit polyclonal anti-cyclin E	Santa Cruz	Cat#sc-33748 AB_638863
Mouse monoclonal anti-NICD	Developmental Studies Hybridoma Bank	Cat# C17.9C6 RRID: AB_528410
Mouse anti-Cut homeobox (Cut)	Developmental Studies Hybridoma Bank	Cat# 2B10 RRID: AB_528186
Mouse monoclonal anti-Wg	Developmental Studies Hybridoma Bank	Cat# 4D4 RRID: AB_528512
Rabbit anti-Sd	Gift from Lei Zhang (Chinese Academy of Sciences)	(Jin et al, 2013)
Rabbit monoclonal anti-DYKDDDDK Tag	Cell Signaling Technology	Cat# 14793; RRID: AB_2572291
Mouse monoclonal anti-Flag	Sigma-Aldrich	Cat# F1804 RRID: AB_262044
Rabbit monoclonal anti-HA-Tag	Cell Signaling Technology	Cat# 3724; RRID: AB_1549585
Mouse monoclonal anti-HA-Tag	Proteintech	Cat#: 66006-2-Ig RRID: AB_ 2881490
Mouse monoclonal anti-Myc-tag	Cell Signaling Technology	Cat# 2276; RRID: AB_331783
Mouse monoclonal anti-NR1D2	Santa Cruz	Cat# sc-100911 RRID: AB_2154757
Mouse anti-dMyc	Developmental Studies Hybridoma Bank	Cat# P4C4-B10 RRID: AB_2753231
Mouse monoclonal anti-Su(H)	Santa Cruz	Cat# sc-398453 RRID: AB_3086636
Rabbit polyclonal anti-Yki	Gift from Duojia Pan (UT Southwestern Medical Center)	(Dong et al, 2007)
Rabbit polyclonal anti-RBPSUH (RBPJ)	Cell Signaling Technology	Cat# 5442, RRID: AB_10695407
Rabbit monoclonal anti-cleaved Notch1	Cell Signaling Technology	Cat# 4147 RRID: AB_2153348
Rabbit monoclonal anti-Pan-TEAD	Cell Signaling Technology	Cat#13295 RRID: AB_2687902
Rabbit monoclonal anti-Yap	Cell Signaling Technology	Cat# 14074 RRID: AB_2650491
Rabbit monoclonal anti-TAZ	Cell Signaling Technology	Cat#70148 RRID: AB_3086635
Rabbit IgG	Beyotime	Cat#A7016 RRID: AB_2905533
Goat anti-Mouse IgG (H + L) Alexa Fluor™ Plus 555	Invitrogen	Cat# A32727; RRID: AB_2633276
Goat anti- IgG (H + L) Alexa Fluor™ Plus 555	Invitrogen	Cat# A32732; RRID: AB_2633281
Goat anti- IgG (H + L) Alexa Fluor™ Plus 647	Invitrogen	Cat# A32733; RRID: AB_2633282
Goat anti-Mouse IgG H&L	Abcam	Cat #ab6702 RRID: AB_956012
Goat anti-Rabbit IgG H&L	Abcam	Cat #ab6708 RRID: AB_956005
Anti-rabbit IgG, HRP-linked Antibody	Cell Signaling Technology	Cat #7074 RRID: AB_2099233
Anti-mouse IgG, HRP-linked Antibody	Cell Signaling Technology	Cat #7076 RRID: AB_330924
**Critical commercial assays**		
Protease inhibitor cocktail	Sigma	Cat# P8340
Hyperactive Universal CUT&Tag Assay Kit	Vazyme	Cat# TD904
Duolink™ In Situ	Sigma-Aldrich	Cat# DUO92008-100RXN; DUO92002-100RXN; DUO92004- 100RXN
Effectene Transfection Reagent	QIAGEN	Cat# 301427
BCA Protein Quantification Kit	Vazyme	Cat# E112-01
Pierce™ Protein A/G Magnetic Beads	Thermo Fisher Scientific	Cat# 88802
FastPure Plasmid Mini Kit	Vazyme	Cat# R211-01
Fetal bovine Serum	Gibco	Cat#C11875500CP
Penicillin and streptomycin	Thermo Fisher Scientific	Cat#15140163
Taq Pro Universal SYBR qPCR Master Mix	Vazyme	Cat# Q712-02
HiScript II 1st Strand cDNA Synthesis Kit	Vazyme	Cat# R211-01
Taq Pro Universal SYBR qPCR Master Mix	Vazyme	Cat# Q712-02
Phanta Max Master Mix	Vazyme	Cat# P515
Super Signal West Pico PLUS	Thermo	Cat# 34577
Schneider’s *Drosophila* Medium	Thermo Fisher	Cat# 21720024
DMEM	Thermo Fisher	Cat# C11330500BT
Normal Goat Serum	Solarbio	Cat# SL038
Anti-HA Magnetic Beads	MedChemExpress	Cat# HY-K0201
Anti-Flag Magnetic Beads	MedChemExpress	Cat# HY-K0207
DAPI	Beyotime	Cat# C1002
Trizol	Thermo Fisher	Cat# 15596026
CellTiter-Glo Luminescent Cell Viability Assay Kit	Promega	Cat #G7572
Lentivirus concentration kit	Genome DiTech	Cat #GM-040801-100
pLKO.TRC.1 (plasmid)	Addgene	RRID: ddgene_10878
pLV-IRES-ZsGreen1 (plasmid)	Gift from Fei Lu	NA
pUAST (plasmid)	Gift from Xiaowei Guo	NA
pMD2.G (plasmid)	Addgene	RRID: ddgene_12259
psPAX2 (plasmid)	Addgene	RRID: ddgene_12260
TrypLE expressed enzyme	Thermo Fisher Scientific	Cat #12604021
Polyethylenimine	Polyscience	Cat #23911-1
neurobasal medium	Gibco	Cat# 12349-015
B27 supplement	Gibco	Cat# 11360-070
penicillin/streptomycin	Invitrogen	Cat# SV30010
basic human fibroblast growth factor	R&D systems	Cat# 4114-TC
human epidermal growth factor	R&D systems	Cat# 236-EG
GlutaMax Supplement	Gibco	Cat# 35050-061
Sodium pyruvate	Gibco	Cat# 11360-070
fetal bovine serum	CellMax	Cat# SA211.02
d-Luciferin potassium salt	Beyotime	Cat# ST196
**Oligonucleotides**		
qPCR for-XIAP-F	ACCGTGCGGTGCTTTAGTT	N/A
qPCR for-XIAP-R	TGCGTGGCACTATTTTCAAGATA	N/A
qPCR for-CYR61-F	CTCGCCTTAGTCGTCACCC	N/A
qPCR for-CYR61-R	CGCCGAAGTTGCATTCCAG	N/A
qPCR for-AXL-F	GTGGGCAACCCAGGGAATATC	N/A
qPCR for-AXL-R	GTACTGTCCCGTGTCGGAAAG	N/A
qPCR for-AREG-F	GTGGTGCTGTCGCTCTTGATA	N/A
qPCR for-AREG-R	CCCCAGAAAATGGTTCACGCT	N/A
qPCR for-CCDN1-F	GCTGCGAAGTGGAAACCATC	N/A
qPCR for-CCDN1-R	CCTCCTTCTGCACACATTTGAA	N/A
qPCR for-HERPUD1-F	ATGGAGTCCGAGACCGAAC	N/A
qPCR for-HERPUD1-R	TTGGTGATCCAACAACAGCTT	N/A
qPCR for-APC-F	AAAATGTCCCTCCGTTCTTATGG	N/A
qPCR for-APC-R	CTGAAGTTGAGCGTAATACCAGT	N/A
qPCR for-HES1 -F	TCAACACGACACCGGATAAAC	N/A
qPCR for-HES1 -R	GCCGCGAGCTATCTTTCTTCA	N/A
qPCR for-TCF7-F	CTGGCTTCTACTCCCTGACCT	N/A
qPCR for-TCF7-R	ACCAGAACCTAGCATCAAGGA	N/A
qPCR for-MYC-F	GGCTCCTGGCAAAAGGTCA	N/A
qPCR for-MYC-R	CTGCGTAGTTGTGCTGATGT	N/A
qPCR for 18S-human F	GGCCCTGTAATTGGAATGAGTC	N/A
qPCR for 18S-human R	CCAAGATCCAACTACGAGCTT	N/A
qPCR for GAPDH-human F	GGAGCGAGATCCCTCCAAAAT	N/A
qPCR for GAPDH-human R	GGCTGTTGTCATACTTCTCATGG	N/A
shNR1D2#1	CCAATGAGTAAGTCTCCATAT	N/A
shNR1D2#2	CCAGTACAAGAAGTGCCTGAA	N/A
shNT	CAACAAGATGAAGAGCACCAA	N/A
qPCR for CUT&Tag-Axl-F	CGCCATGATACCTGGTTAAG	N/A
qPCR for CUT&Tag-Axl-R	CGCTTGAGCCCAGGAGTTC	N/A
qPCR for CUT&Tag-Hey1-F	GCCTCGGTCCCAGAAATCA	N/A
qPCR for CUT&Tag-Hey1-R	CGTCCACAGTGGCCACCA	N/A
qPCR for CUT&Tag-TCF-F	GAACTTACTACAGTCAAAGCAGCT	N/A
qPCR for CUT&Tag-TCF-R	CCGCTTTGGTCTTGATCTTG	N/A
DNA Spike in F	GCCTTCTTCCCATTTCTGATCC	N/A
DNA Spike in R	CACGAATCAGCGGTAAAGGT	N/A
PCR for E75-F	ATGGTTTGTGCAATGCAAGA	N/A
PCR for E75-R	TTACGCCTCCAGCATTACCTT	N/A
PCR for NR1D2-F	ATGGAGGTGAATGCAGGAGG	N/A
PCR for NR1D2-R	TTAAGGGTGAACTTTAAAGGCCA	N/A
**Software and algorithms**		
Prism 8.0	GraphPad Software	RRID:SCR_002798
ImageJ	https://imagej.net/	RRID:SCR_003070
GEPIA2	http://gepia2.cancer-pku.cn/#index	NA
NIS-Element Viewer	https://www.microscope.healthcare.nikon.com/	NA
BioRender	https://www.biorender.com	NA
**Deposited data**		
RNA-seq and CUT&Tag-seq raw data NCBI accession number	https://www.ncbi.nlm.nih.gov/bioproject/1067005	RNA-seq and CUT&Tag-seq raw data NCBI accession number
**Experimental models: Cell lines**		
*Drosophila* S2 cells	Gift from Jose´ C. Pastor-Pareja (Tsinghua University)	FBtc9000001 RRID: CVCL_TZ72
U87 MG	American Type Cell Collection (ATCC)	HTB-14 RRID: CVCL_0022
MGG6	Gift from Jeremy Rich (University of Pittsburgh)	RRID: CVCL_D1H2
MGG4	Gift from Jeremy Rich (University of Pittsburgh)	RRID: CVCL_D1H1
HEK293T	American Type Cell Collection (ATCC)	CRL-3216 RRID: CVCL_0063

### Methods and protocols

#### Fly husbandry and genetics

*Drosophila* stocks and crosses were maintained on standard food at a temperature of 25 °C unless otherwise specified. A comprehensive list of all *Drosophila* lines utilized can be found in the reagents and tools table, and detailed genotypes for each figure panel are provided in Dataset EV1. The standard food composition consisted of a cornmeal-yeast mixture, comprising 50 g of corn flour, 30 g of brown sugar, 24.5 g of dry yeast, 7.25 g of white sugar, 9 g of agar, 4.4 mL of propionic acid, 12.5 mL of ethanol, and 1.25 g of nipagin per liter.

#### Co-IP and Western blot

Tumor samples or cells expressing indicated constructs with corresponding genotypes were collected and lysed in NP40 buffer with PMSF. Subsequently, the resulting cell lysate was combined with pre-washed magnetic beads, and the mixture was subjected to gentle rotation at room temperature for 30 min. This allowed for efficient binding of the target proteins to the magnetic beads. A magnetic separation rack was utilized, facilitating quick and easy isolation of the beads. The pre-cleared lysate, devoid of magnetic beads, was then carefully transferred to a clean tube, ensuring the retention of the protein content for subsequent analysis. Then, add primary antibody or HA-conjugated beads to 200 μl cell lysate, and incubate overnight at 4 °C. After that, the pre-washed magnetic beads were added to the lysate and antibody solution (omit this step for antibody-conjugated beads), the bound protein can be finally separated through the magnetic beads. Finally, the coprecipitated protein was eluted for western blot analysis. The proteins were separated using SDS-PAGE and then transferred onto PVDF membranes. To prevent non-specific binding, the membranes were blotted with 5% skim milk powder in TBST for 60 min. Following this, the membranes were incubated overnight at 4 °C with primary antibodies. The following antibodies were used: HA (CST, 1:1000), Flag (Proteintech, 1:1000), Myc (CST, 1:1000), NICD (DSHB, 1:1000), Yki (1:1000), TEAD (CST, 1:500), RBPJ (CST, 1:500), TAZ (CST, 1:500), and NICD1(CST, 1:500). The membranes were washed three times with TBST to remove any unbound antibodies. Afterward, the membranes were incubated with HRP-conjugated secondary antibodies to enable visualization of the target proteins. The following antibodies for western blotting were used: primary antibodies, rabbit anti-Flag (1:6,000), and secondary antibody anti-rabbit-HRP (1:8,000).

#### Duolink in situ proximity ligation assay (PLA)

Wing discs and cultured cells were harvested and subsequently fixed. Following fixation, the samples were blocked to prevent non-specific binding and then subjected to a series of washes. The indicated primary antibodies from different origins (mouse or rabbit) were applied and allowed to incubate with the samples. Primary antibodies used *Drosophila* tissues include mouse anti-Flag (Sigma, 1:200) and rabbit anti-HA (CST, 1:200), rabbit anti-Flag (CST, 1:200) and mouse anti-Su(H) (Santa Cruz, 1:100), mouse anti-NICD (DSHB, 1:100), rabbit anti-Sd (from Lei Zhang, 1:100), rabbit anti-Yki (from Duojia Pan, 1:100). Primary antibodies used in mammalian cells include mouse anti-NR1D2 (Santa Cruz, 1:100), rabbit anti-TAZ, rabbit anti-pan TEAD (CST, 1:200), rabbit anti-Yap1 (CST, 1:200), rabbit anti-RBPJ (CST, 1:200) and rabbit anti-NICD1 (CST, 1:200). To enable detection, secondary antibodies conjugated to complementary PLA (Proximity Ligation Assay) probes were added. The Duolink method was employed, involving hybridization, ligation, amplification, and detection steps, following the guidelines provided by the manufacturer. This ensured accurate and reliable detection of protein-protein interactions or protein localization. For control samples, primary antibodies were omitted, and only secondary antibodies were added, which were subsequently detected using standard methods.

#### Cell proliferation assay

A total of 2500 cells were plated in each well of the 96-well plates. Subsequently, the Cell Titer-Glo Luminescent Cell Viability Assay Kit (Promega, Cat #G7572) was employed to assess cell viability at the indicated time points following the manufacturer’s instructions.

#### Mouse experiments

All mouse experiments were conducted in accordance with the relevant guidelines and under an animal protocol (#XQ-19-028) approved by the Institutional Animal Care and Use Committee of Westlake University. The intracranial transplantation of GSCs followed a previously described method (Liu et al, 2023). Briefly, GSC spheres were dissociated into single cells with TrypLE expressed enzyme (Thermo Fisher Scientific, Cat#12604021), and 10,000 cells were injected into the right cerebral cortex of NSG (NOD.Cg-Prkdc scid Il2rg tm1Wjl /SzJ) immunocompromised mice (Shanghai Model Organisms Center, Inc.) individually. As to in vivo tumor growth comparison, mice were narcotized and then imaged by bioluminescence imaging at the indicated time. In parallel survival experiments, mice were monitored until the development of neurological signs or morbidity symptoms.

#### shRNA Plasmids

The shRNA sequences were inserted into the pLKO.TRC.1 plasmid (Addgene, Cat#10878, RRID: Addgene_10878).

#### Lentivirus production

HEK293T cells were co-transfected with a lentiviral expression vector, the envelope plasmid pMD2.G (Addgene, Cat#12259, RRID: Addgene_12259), and the packaging plasmid psPAX2 (Addgene, Cat#12260, RRID: Addgene_12260) using polyethylenimine (PEI) (Polyscience, Cat # 23966-1) according to the manufacturer’s instructions. After 48 h of transfection, lentiviral particles were harvested and concentrated using the lentivirus concentration kit (Genomeditech, Cat# GM-040801-100).

#### Immunofluorescence and imaging

The imaginal discs were dissected from the third-instar larvae on the 6th day after egg laying unless otherwise indicated. Discs were dissected in cold PBS and fixed with 4% paraformaldehyde for 15 min at room temperature, then washed for 3 × 5 min with PBS containing 0.1% Triton X-100 solution (PBST). Samples were blocked in 10% goat serum in PBST for 30 min after fixed and washed, and then incubated with primary antibody at 4 °C overnight. Primary antibodies used include mouse Anti-beta Galactosidase (1:100, DSHB), mouse anti-Br (1:100, DSHB), rat anti-Elav (1:100, DSHB), mouse anti-Mmp-1 (1:100, DSHB), mouse anti-wg (1:100, DSHB), Cyclin E (1:100, Santa Cruz), rabbit anti-Dcp1 (CST, 1:100), mouse anti-dMyc (1:50, DSHB), mouse anti-Cut (1:100, DSHB), mouse anti-NICD (1:100, DSHB). After that, samples were washed 3 × 10 min with PBST and incubated with secondary antibody and DAPI at 1:200 in PBST for 2 h. The images were performed with the Nikon A1R confocal Microscope. Images were processed with NIS-Element Viewer and ImageJ software.

#### Total RNA extraction and quantitative RT-PCR

In this study, a total of 1 million GSCs or a total of 100 eye-antenna-disc tumors or a total of 150 wing imaginal discs were harvested at the late stage of third-instar larvae for total RNA extraction of each biological replicate across different genotypes by using Trizol (Invitrogen, Carlsbad, CA) according to the manufacturer’s instructions. The RNA quality examination was conducted as follows: the purity of the sample was determined by NanoPhotometer^®^ (IMPLEN, CA, USA), while the concentration and integrity of RNA samples were detected by using an Agilent 2100 RNA nano 6000 assay kit (Agilent Technologies, CA, USA). Subsequently, the extracted RNA was reverse-transcribed into complementary DNA (cDNA) using a cDNA reverse transcription kit (Vazyme). Taq Pro Universal SYBR qPCR Master Mix (Vazyme) and the corresponding primers were utilized for quantitative polymerase chain reaction (qPCR), and the qPCR reactions were performed on a Jena Qtower384G Real-Time PCR System. 18S and GAPDH were used as the internal control in mammalian cells, and Spike-in served as an internal control for CUT&Tag qPCR.

#### Library preparation for RNA sequencing

For each sample, a total amount of 1–3 μg RNA was used as the input for library preparation by strictly following the standard protocol of VAHTS Universal V6 RNA-seq Library Prep Kit for Illumina^®^ (NR604-01/02). Briefly, mRNA was purified by using poly-T oligo-attached magnetic beads from total RNA. The short fragments of mRNA were obtained by adding the fragmentation buffer. After the first strand of cDNA was synthesized by using random hexamer primer and RNase H, the second strand synthesis was performed subsequently by using buffer, dNTPs, DNA polymerase I, and RNase H. And then, the double-stranded cDNA was purified by using QiaQuick PCR kit or AMPure P beads. The purified products of each sample were repaired at the end, added tail, and connected to the sequencing connector, then the appropriate fragment size was selected, and the final cDNA library was obtained by PCR amplification for further sequencing performed on Illumina NovaSeq 6000 platform with NovaSeq 6000 S4 Reagent kit V1.5.

#### RNA-seq data processing and analysis

Both wing disc and eye-antennal disc transcriptomic data were processed and analyzed as follows: Data quality control and reads statistics were performed by using FastQC (v0.11.8) software (https://github.com/s-andrews/FastQC/releases/tag/v0.11.8). Low-quality reads were removed, and the maintained high-quality reads (Q30 > 90%) were mapped to the Ensemble (Martin et al, 2023) *Drosophila melanogaster* reference genome (Drosophila_melanogaster.BDGP6.32.108) by using Hisat2 (Kim et al, 2019). HTSeq (Anders et al, 2015) was applied for gene feature counting with default settings. Differential gene expression identification and functional annotation were conducted in R (v4.2.0). Genes meeting |fold-change| >1.5 and false discovery rate (FDR) <0.05 were identified as differentially expressed genes (DEGs) by using edgeR (Robinson et al, 2010) with the “RLE” method. The gene ID transformation was performed by using biomaRt (Durinck et al, 2009). DEGs were annotated against terms in Kyoto Encyclopedia of Genes and Genomes (KEGG) database (Kanehisa et al, 2017; Kanehisa and Goto, 2000) and Gene Ontology (GO) consortium (Ashburner et al, 2000) and by using clusterProfiler package (Yu et al, 2012). Data visualization was relied on pheatmap (v1.0.12), RColorBrewer (v1.1-3), and ggplot2 (v3.4.4) packages. Gene set enrichment analysis (GSEA) (Mootha et al, 2003; Subramanian et al, 2005) was performed locally with a gene list of each pathway from FlyBase (https://flybase.org) (Gramates et al, 2022).

#### Cleavage under targets and tagmentation (CUT&Tag)

All CUT&Tag experiments were conducted by strictly following the manual instructions of the Hyperactive Universal CUT&Tag Assay Kit for Illumina Pro (Vazyme, TD904). Briefly, ~1 × 10^5^ cells were collected from wing imaginal disc or eye-antennal disc tumors for each sample group (at least three biological replicates). These cells were incubated and mixed (2–3 times) with Concanavalin A beads Pro at room temperature for 10 min. Then, the liquid was removed, and the specific primary antibodies were added with ice-cold 50 μl Antibody Buffer. The primary antibodies used include Myc-tag (CST, 1:100), NICD (DSHB, 1:50), Flag-tag (Sigma, 1:50) in *Drosophila* tissues; Rabbit IgG (Beyotime, 1:100) or HA-tag (CST, 1:100) for CUT&Tag qPCR in mammalian cells. After incubating overnight at 4 °C, the liquid was removed, and the secondary antibody diluted by Dig-Wash Buffer (1:100) was added for incubation at room temperature for 1 h. For each sample, 2 μl Hyperactive pA/G-Transposon Pro mixed with 98 μl Dig-300 Buffer were added and incubated at room temperature for 1 h. After gently washing with Dig-300 Buffer, the fragmentation of each sample was conducted by incubating the mixture of 40 μl Dig-300 Buffer, 10 μl 5 × TTBL, and the products obtained in the previous step at 37 °C for 1 h. About 1 pg spike-in was added for internal control before DNA extraction. Then, DNA extraction was performed by incubating the products with DNA Extract Beads Pro diluting in 50 μl 2 × B&W Buffer at room temperature for 20 min. The i7 and i5 Indexed Primer were combined for PCR amplification with the recommended cycle number (9–11). Finally, only the CUT&Tag libraries passing fragment analyzer quality control (Fragment Analyzer-12/96) were then sequenced on Illumina NovaSeq 6000.

#### CUT&Tag-Seq data processing and analysis

The analytic procedures of CUT&Tag-seq data were referred to recommended pipelines (Henikoff et al, 2020). The raw sequencing data were processed by using FastQC (v0.11.8) software (https://github.com/s-andrews/FastQC/releases/tag/v0.11.8) for quality control. Adapters were removed and reads were trimmed by using Cutadapt (v1.18) (Martin, 2011). The clean reads were first mapped to the Ensemble (Martin et al, 2023) *Escherichia coli* (*E. coli*) reference genome (GCF_000005845.2_ASM584v2_genomic) by using Bowtie2 (v2.4.2) (Langmead and Salzberg, 2012), the unrecognized reads were subsequently mapped to *Drosophila melanogaster* reference genome (Drosophila_melanogaster.BDGP6.32.108) with the parameters “--end-to-end --very-sensitive --no-mixed --no-discordant --phred33 -I 0 -X 1000 --no-unal”. After using SAMtools (v1.11) (Danecek et al, 2021) to transform SAM files into BAM files, Picard (v2.25.1) (https://broadinstitute.github.io/picard/) was applied to remove duplicates, and the reads were selectively maintained when meeting “mapping_quality ≥20”. The final BAM files of the same genotype among the replicates were merged into one for peak calling, which is performed by using model-based analysis of ChIP-seq (MACS2, v2.2.6) (Zhang et al, 2008b) with settings “-g dm -f BAMPE -q 0.05 --keep-dup all”. Peaks were annotated by using the ChIPseeker (Yu et al, 2015) package in R (v4.2.0), and the peak distribution was visualized by using *plotAnnoBar* and *plotDistToTSS* functions. The final BAM files were transformed into bigwig files for signal enrichment visualization by using the *bamCoverage* function of BEDTools (v2.30.0) (Quinlan and Hall, 2010) with settings “--binSize 20 --normalizeUsing BPM”. The average normalized signal values of peaks in the *Drosophila* genome region were calculated and visualized by using DeepTools (v3.5.1) (Ramírez et al, 2016), in which regions within 3 kb distance relative to the transcriptional start sites (TSS) were included, and gene body region was scaled into 5 kb length. Integrative genomics viewer (IGV) (Robinson et al, 2011) was applied to visualize the peaks on specific genome regions of interested genes. The binding motifs were identified by using the *findMotifsGenome* or *findMotifs* functions of Homer (v4.11) (Heinz et al, 2010) regarding to conditions. The motif scanning algorithm, Find Individual Motif Occurrences (FIMO, v5.5.5) (Grant et al, 2011) was applied in a set of sequences to determine all the positions where transcription factor motifs match (*p* < 0.0001) via The MEME Suite (Bailey et al, 2015).

#### Public data analysis

The binding regions of Yki and Su(H) on *Drosophila melanogaster* genome were annotated by using public datasets GSE38594 (Oh et al, 2013) and GSE41429 (Djiane et al, 2013), respectively. The DEG list between siNR1D2 and siControl LN-18 cells was retrieved from the Supplementary Table S1 and S2 of our previous publication(Yu et al, 2018), and the over-representation analyses (ORA) were conducted using clusterProfiler package (Yu et al, 2012). The analysis of *NR1D2* relative expression level between GSC and NSC was conducted by using the processed data (CPM) from GSE54791 (Mack et al, 2019) for the Mann–Whitney test. The correlation analysis of *NR1D2* expression with other genes of interest were performed by using the GEPIA2 web server (Tang et al, 2019).

#### Quantification and statistical analysis

In Figs. 1F–H, 3B–D,F and EV1D–H,L, EV3G–H,K,L the region of interest (ROI) in each clone and the wild-type cell was subjected to cycling and measurements using ImageJ. The size of the tumors or ROI was quantified by measuring the area of the GFP-positive region in each sample. All statistical analyses were performed with GraphPad Prism 8.0. software. Data represents mean values ± SD. Statistical significance was assessed using appropriate methods depending on the experimental design. For comparisons between the two groups, an unpaired two-tailed Student’s *t*-test was performed. For experiments involving three or more groups, ordinary one-way ANOVA or two-way ANOVA analysis was conducted. Additionally, in survival analysis, the log-rank (Mantel-Cox) test was employed. The investigators analyzing the data were not blind to the identity of the samples. The specific statistical tests used for each analysis are indicated in the corresponding figures. *p* value <0.05 was considered significant, *p < 0.05, **p < 0.01, ***p < 0.001, ****p < 0.0001.

## Supplementary information

Peer Review File

Dataset EV1

Source data Fig. 1

Source data Fig. 2

Source data Fig. 3

Source data Fig. 4

Source data Fig. 5

Source data Fig. 6

Expanded View Figures

## Data Availability

All sequencing data of this study is deposited in the National Center for Biotechnology Information Sequence Read Archive (Rubin et al, 2000) with the accession number BioProject: PRJNA1067005. The source data of this paper are collected in the following database record: biostudies:S-SCDT-10_1038-S44318-024-00290-3.

## References

[CR1] Ahmed SMH, Maldera JA, Krunic D, Paiva-Silva GO, Pénalva C, Teleman AA, Edgar BA (2020) Fitness trade-offs incurred by ovary-to-gut steroid signalling in *Drosophila*. Nature 584:415. 32641829 10.1038/s41586-020-2462-y PMC7442704

[CR2] Anders S, Pyl PT, Huber W (2015) HTSeq-a Python framework to work with high-throughput sequencing data. Bioinformatics 31:166–169 25260700 10.1093/bioinformatics/btu638 PMC4287950

[CR3] Anurag M, Ellis MJ, Haricharan S (2018) DNA damage repair defects as a new class of endocrine treatment resistance driver. Oncotarget 9:36252–36253 30555626 10.18632/oncotarget.26363 PMC6284739

[CR4] Asemota S, Ponnusamy S, Thiyagarajan T, Narayanan R (2022) Androgen receptor (AR) agonists inhibit AR- and estrogen-receptor-positive breast cancer. Cancer Res 82:1777

[CR5] Ashburner M, Ball CA, Blake JA, Botstein D, Butler H, Cherry JM, Davis AP, Dolinski K, Dwight SS, Eppig JT et al (2000) Gene ontology: tool for the unification of biology. The Gene Ontology Consortium. Nat Genet 25:25–29 10802651 10.1038/75556 PMC3037419

[CR6] Bailey TL, Johnson J, Grant CE, Noble WS (2015) The MEME suite. Nucleic Acids Res 43:W39–49 25953851 10.1093/nar/gkv416 PMC4489269

[CR7] Bao SD, Wu QL, McLendon RE, Hao YL, Shi Q, Hjelmeland AB, Dewhirst MW, Bigner DD, Rich JN (2006) Glioma stem cells promote radioresistance by preferential activation of the DNA damage response. Nature 444:756–760 17051156 10.1038/nature05236

[CR8] Bilder D, Li M, Perrimon N (2000) Cooperative regulation of cell polarity and growth by tumor suppressors. Science 289:113–116 10884224 10.1126/science.289.5476.113

[CR9] Bilder D, Ong K, Hsi TC, Adiga K, Kim J (2021) Tumour-host interactions through the lens of *Drosophila*. Nat Rev Cancer 21:687–700 34389815 10.1038/s41568-021-00387-5 PMC8669834

[CR10] Brankovic-Magic M, Jankovic R, Neskovic-Konstantinovic Z, Nikolic-Vukosavljevic D (2002) Progesterone receptor status of breast cancer metastases. J Cancer Res Clin Oncol 128:55–60 11862473 10.1007/s00432-001-0299-9 PMC12164405

[CR11] Brumby AM, Richardson HE (2003) scribble mutants cooperate with oncogenic Ras or Notch to cause neoplastic overgrowth in *Drosophila*. EMBO J 22:5769–5779 14592975 10.1093/emboj/cdg548 PMC275405

[CR12] Buttigliero C, Tucci M, Bertaglia V, Vignani F, Bironzo P, Di Maio M, Scagliotti GV (2015) Understanding and overcoming the mechanisms of primary and acquired resistance to abiraterone and enzalutamide in castration resistant prostate cancer. Cancer Treat Rev 41:884–892 26342718 10.1016/j.ctrv.2015.08.002

[CR13] Caffa I, Spagnolo V, Vernieri C, Valdemarin F, Becherini P, Wei M, Brandhorst S, Zucal C, Driehuis E, Ferrando L et al (2020) Fasting-mimicking diet and hormone therapy induce breast cancer regression. Nature 583:620–624 32669709 10.1038/s41586-020-2502-7 PMC7881940

[CR14] Conway-Campbell BL, Wooh JW, Brooks AJ, Gordon D, Brown RJ, Lichanska AM, Chin HS, Barton CL, Boyle GM, Parsons PG et al (2007) Nuclear targeting of the growth hormone receptor results in dysregulation of cell proliferation and tumorigenesis. Proc Natl Acad Sci USA 104:13331–13336 17690250 10.1073/pnas.0600181104 PMC1948913

[CR15] Danecek P, Bonfield JK, Liddle J, Marshall J, Ohan V, Pollard MO, Whitwham A, Keane T, McCarthy SA, Davies RM et al (2021) Twelve years of SAMtools and BCFtools. Gigascience 10:giab008 33590861 10.1093/gigascience/giab008 PMC7931819

[CR16] Dierickx P, Emmett MJ, Jiang CJ, Uehara K, Liu ML, Adlanmerini M, Lazar MA (2019) SR9009 has REV-ERB-independent effects on cell proliferation and metabolism. Proc Natl Acad Sci USA 116:12147–12152 31127047 10.1073/pnas.1904226116 PMC6589768

[CR17] Djiane A, Krejci A, Bernard F, Fexova S, Millen K, Bray SJ (2013) Dissecting the mechanisms of Notch induced hyperplasia. EMBO J 32:60–71 23232763 10.1038/emboj.2012.326 PMC3545308

[CR18] Dong J, Feldmann G, Huang J, Wu S, Zhang N, Comerford SA, Gayyed MF, Anders RA, Maitra A, Pan D (2007) Elucidation of a universal size-control mechanism in *Drosophila* and mammals. Cell 130:1120–1133 17889654 10.1016/j.cell.2007.07.019 PMC2666353

[CR19] Durinck S, Spellman PT, Birney E, Huber W (2009) Mapping identifiers for the integration of genomic datasets with the R/Bioconductor package biomaRt. Nat Protoc 4:1184–1191 19617889 10.1038/nprot.2009.97 PMC3159387

[CR20] Fredriksson S, Gullberg M, Jarvius J, Olsson C, Pietras K, Gústafsdóttir SM, Östman A, Landegren U (2002) Protein detection using proximity-dependent DNA ligation assays. Nat Biotechnol 20:473–477 11981560 10.1038/nbt0502-473

[CR21] Gavish A, Mints M, Greenwald AC, Hoefflin R, Simkin D, Tschernichovsky R, Darnell NG, Somech E, Barbolin C, Antman T et al (2023) Hallmarks of transcriptional intratumour heterogeneity across a thousand tumours. Nature 618:598–606 37258682 10.1038/s41586-023-06130-4

[CR22] Gimple RC, Yang KL, Halbert ME, Agnihotri S, Rich JN (2022) Brain cancer stem cells: resilience through adaptive plasticity and hierarchical heterogeneity. Nat Rev Cancer 22:497–514 35710946 10.1038/s41568-022-00486-x

[CR23] Gonzalez-Mora AM, Garcia-Lopez P (2021) Estrogen receptors as molecular targets of endocrine therapy for glioblastoma. Int J Mol Sci 22:12404 10.3390/ijms222212404 PMC8626012 34830286

[CR24] Gramates LS, Agapite J, Attrill H, Calvi BR, Crosby MA, Dos Santos G, Goodman JL, Goutte-Gattat D, Jenkins VK, Kaufman T et al (2022) FlyBase: a guided tour of highlighted features. Genetics 220:iyac035 35266522 10.1093/genetics/iyac035 PMC8982030

[CR25] Grant CE, Bailey TL, Noble WS (2011) FIMO: scanning for occurrences of a given motif. Bioinformatics 27:1017–1018 21330290 10.1093/bioinformatics/btr064 PMC3065696

[CR26] Greenman C, Stephens P, Smith R, Dalgliesh GL, Hunter C, Bignell G, Davies H, Teague J, Butler A, Stevens C et al (2007) Patterns of somatic mutation in human cancer genomes. Nature 446:153–158 17344846 10.1038/nature05610 PMC2712719

[CR27] Hanker AB, Sudhan DR, Arteaga CL (2020) Overcoming endocrine resistance in breast cancer. Cancer Cell 37:496–513 32289273 10.1016/j.ccell.2020.03.009 PMC7169993

[CR28] Heinz S, Benner C, Spann N, Bertolino E, Lin YC, Laslo P, Cheng JX, Murre C, Singh H, Glass CK (2010) Simple combinations of lineage-determining transcription factors prime cis-regulatory elements required for macrophage and B cell identities. Mol Cell 38:576–589 20513432 10.1016/j.molcel.2010.05.004 PMC2898526

[CR29] Henikoff S, Henikoff JG, Kaya-Okur HS, Ahmad K (2020) Efficient chromatin accessibility mapping in situ by nucleosome-tethered tagmentation. Elife 9:e63274 10.7554/eLife.63274 PMC7721439 33191916

[CR30] Hiruma K, Riddiford LM (2004) Differential control of MHR3 promoter activity by isoforms of the ecdysone receptor and inhibitory effects of E75A and MHR3. Dev Biol 272:510–521 15282165 10.1016/j.ydbio.2004.04.028

[CR31] Huang JB, Wu S, Barrera J, Matthews K, Pan DJ (2005) The Hippo signaling pathway coordinately regulates cell proliferation and apoptosis by inactivating Yorkie, the homolog of YAP. Cell 122:421–434 16096061 10.1016/j.cell.2005.06.007

[CR32] Huet F, Ruiz C, Richards G (1995) Sequential gene activation by ecdysone in *Drosophila melanogaster*: the hierarchical equivalence of early and early late genes. Development 121:1195–1204 7743931 10.1242/dev.121.4.1195

[CR33] Jaumouillé E, Almeida PM, Stähli P, Koch R, Nagoshi E (2015) Transcriptional regulation via nuclear receptor crosstalk required for the circadian clock. Curr Biol 25:1502–1508 26004759 10.1016/j.cub.2015.04.017 PMC4454776

[CR34] Jiang YR, Seimiya M, Schlumpf TB, Paro R (2018) An intrinsic tumour eviction mechanism in mediated by steroid hormone signalling. Nat Commun 9:3293 30120247 10.1038/s41467-018-05794-1 PMC6098038

[CR35] Jin YY, Xu JJ, Yin MX, Lu Y, Hu LX, Li PX, Zhang P, Yuan ZQ, Ho MS, Ji HB, et al (2013) Brahma is essential for intestinal stem cell proliferation and regulated by Hippo signaling. Elife 2:e00999 10.7554/eLife.00999 PMC3796317 24137538

[CR36] Johnston DM, Sedkov Y, Petruk S, Riley KM, Fujioka M, Jaynes JB, Mazo A (2011) Ecdysone- and NO-mediated gene regulation by competing EcR/Usp and E75A nuclear receptors during development. Mol Cell 44:51–61 21981918 10.1016/j.molcel.2011.07.033 PMC3190167

[CR37] Kanehisa M, Furumichi M, Tanabe M, Sato Y, Morishima K (2017) KEGG: new perspectives on genomes, pathways, diseases and drugs. Nucleic Acids Res 45:D353–D361 27899662 10.1093/nar/gkw1092 PMC5210567

[CR38] Kanehisa M, Goto S (2000) KEGG: kyoto encyclopedia of genes and genomes. Nucleic Acids Res 28:27–30 10592173 10.1093/nar/28.1.27 PMC102409

[CR39] Katsukawa M, Ohsawa S, Zhang L, Yan Y, Igaki T (2018) Serpin facilitates tumor-suppressive cell competition by blocking Toll-mediated Yki activation in *Drosophila*. Curr Biol 28:1756–1767.e1756 29804808 10.1016/j.cub.2018.04.022

[CR40] Kaya-Okur HS, Wu SJ, Codomo CA, Pledgers ES, Bryson TD, Henikoff JG, Ahmad K, Henikoff S (2019) CUT&Tag for efficient epigenomic profiling of small samples and single cells. Nat Commun 10:1930 10.1038/s41467-019-09982-5 PMC6488672 31036827

[CR41] Kim D, Paggi JM, Park C, Bennett C, Salzberg SL (2019) Graph-based genome alignment and genotyping with HISAT2 and HISAT-genotype. Nat Biotechnol 37:907–915 31375807 10.1038/s41587-019-0201-4 PMC7605509

[CR42] Kim W, Khan SK, Gvozdenovic-Jeremic J, Kim Y, Dahlman J, Kim H, Park O, Ishitani T, Jho EH, Gao B et al (2017) Hippo signaling interactions with Wnt/β-catenin and Notch signaling repress liver tumorigenesis. J Clin Invest 127:137–152 27869648 10.1172/JCI88486 PMC5199712

[CR43] Kong D, Zhao S, Xu W, Dong J, Ma X (2022) Fat body-derived Spz5 remotely facilitates tumor-suppressive cell competition through Toll-6-alpha-Spectrin axis-mediated Hippo activation. Cell Rep 39:110980 35732124 10.1016/j.celrep.2022.110980

[CR44] Kotsopoulos J (2019) Menopausal hormones: definitive evidence for breast cancer. Lancet 394:1116–1118 31474333 10.1016/S0140-6736(19)31901-4

[CR45] Langmead B, Salzberg SL (2012) Fast gapped-read alignment with Bowtie 2. Nat Methods 9:357–359 22388286 10.1038/nmeth.1923 PMC3322381

[CR46] Lee T, Luo L (2001) Mosaic analysis with a repressible cell marker (MARCM) for *Drosophila* neural development. Trends Neurosci 24:251–254 11311363 10.1016/s0166-2236(00)01791-4

[CR47] Liu L, Liu Z, Liu Q, Wu W, Lin P, Liu X, Zhang Y, Wang D, Prager BC, Gimple RC et al (2023) LncRNA INHEG promotes glioma stem cell maintenance and tumorigenicity through regulating rRNA 2’-O-methylation. Nat Commun 14:7526 37980347 10.1038/s41467-023-43113-5 PMC10657414

[CR48] Liu P, Guo YF, Xu WY, Song S, Li XQ, Wang X, Lu JY, Guo XW, Richardson HE, Ma XJ (2022a) Ptp61F integrates Hippo, TOR, and actomyosin pathways to control three-dimensional organ size. Cell Rep 41:111640 36384105 10.1016/j.celrep.2022.111640

[CR49] Liu Y, Saavedra P, Perrimon N (2022b) Cancer cachexia: lessons from *Drosophila*. Dis Model Mech 15:dmm049298 35319749 10.1242/dmm.049298 PMC8961677

[CR50] Ma X, Lu JY, Dong Y, Li D, Malagon JN, Xu T (2017) PP6 disruption synergizes with oncogenic Ras to promote JNK-dependent tumor growth and invasion. Cell Rep 19:2657–2664 28658615 10.1016/j.celrep.2017.05.092 PMC5580353

[CR51] Mack SC, Singh I, Wang X, Hirsch R, Wu Q, Villagomez R, Bernatchez JA, Zhu Z, Gimple RC, Kim LJY et al (2019) Chromatin landscapes reveal developmentally encoded transcriptional states that define human glioblastoma. J Exp Med 216:1071–1090 30948495 10.1084/jem.20190196 PMC6504206

[CR52] Martin FJ, Amode MR, Aneja A, Austine-Orimoloye O, Azov AG, Barnes I, Becker A, Bennett R, Berry A, Bhai J et al (2023) Ensembl 2023. Nucleic Acids Res 51:D933–D941 36318249 10.1093/nar/gkac958 PMC9825606

[CR53] Martin M (2011) Cutadapt removes adapter sequences from high-throughput sequencing reads. EMBnet J 17:10–12

[CR54] Metcalfe C, Friedman LS, Hager JH (2018) Hormone-targeted therapy and resistance. Annu Rev Cancer Biol 2:291–312

[CR55] Mootha VK, Lindgren CM, Eriksson KF, Subramanian A, Sihag S, Lehar J, Puigserver P, Carlsson E, Ridderstråle M, Laurila E et al (2003) PGC-1alpha-responsive genes involved in oxidative phosphorylation are coordinately downregulated in human diabetes. Nat Genet 34:267–273 12808457 10.1038/ng1180

[CR56] Nakahashi H, Kwon KRK, Resch W, Vian L, Dose M, Stavreva D, Hakim O, Pruett N, Nelson S, Yamane A et al (2013) A genome-wide map of CTCF multivalency redefines the CTCF code. Cell Rep 3:1678–1689 23707059 10.1016/j.celrep.2013.04.024 PMC3770538

[CR57] Neto-Silva RM, de Beco S, Johnston LA (2010) Evidence for a growth-stabilizing regulatory feedback mechanism between Myc and Yorkie, the homolog of Yap. Dev Cell 19:507–520 20951343 10.1016/j.devcel.2010.09.009 PMC2965774

[CR58] Oh H, Slattery M, Ma L, Crofts A, White KP, Mann RS, Irvine KD (2013) Genome-wide association of Yorkie with chromatin and chromatin-remodeling complexes. Cell Rep 3:309–318 23395637 10.1016/j.celrep.2013.01.008 PMC3633442

[CR59] Orr BA, Bai HB, Odia Y, Jain D, Anders RA, Eberhart CG (2011) Yes-associated protein 1 is widely expressed in human brain tumors and promotes glioblastoma growth. J Neuropath Exp Neur 70:568–577 21666501 10.1097/NEN.0b013e31821ff8d8 PMC3130608

[CR60] Pagliarini RA, Xu T (2003) A genetic screen in for metastatic behavior. Science 302:1227–1231 14551319 10.1126/science.1088474

[CR61] Purow BW, Haque RM, Noel MW, Su Q, Burdick MJ, Lee J, Sundaresan T, Pastorino S, Park JK, Mikolaenko I et al (2005) Expression of Notch-1 and its ligands, Delta-like-1 and Jagged-1, is critical for glioma cell survival and proliferation. Cancer Res 65:2353–2363 15781650 10.1158/0008-5472.CAN-04-1890

[CR62] Quinlan AR, Hall IM (2010) BEDTools: a flexible suite of utilities for comparing genomic features. Bioinformatics 26:841–842 20110278 10.1093/bioinformatics/btq033 PMC2832824

[CR63] Rabinovich D, Yaniv SP, Alyagor I, Schuldiner O (2016) Nitric oxide as a switching mechanism between axon degeneration and regrowth during developmental remodeling. Cell 164:170–182 26771490 10.1016/j.cell.2015.11.047 PMC5086089

[CR64] Ramírez F, Ryan DP, Grüning B, Bhardwaj V, Kilpert F, Richter AS, Heyne S, Dündar F, Manke T (2016) deepTools2: a next generation web server for deep-sequencing data analysis. Nucleic Acids Res 44:W160–165 27079975 10.1093/nar/gkw257 PMC4987876

[CR65] Rani A, Stebbing J, Giamas G, Murphy J (2019) Endocrine resistance in hormone receptor positive breast cancer-from mechanism to therapy. Front Endocrinol 10:245 10.3389/fendo.2019.00245 PMC6543000 31178825

[CR66] Reinking J, Lam MM, Pardee K, Sampson HM, Liu S, Yang P, Williams S, White W, Lajoie G, Edwards A et al (2005) The Drosophila nuclear receptor e75 contains heme and is gas responsive. Cell 122:195–207 16051145 10.1016/j.cell.2005.07.005

[CR67] Robinson JT, Thorvaldsdóttir H, Winckler W, Guttman M, Lander ES, Getz G, Mesirov JP (2011) Integrative genomics viewer. Nat Biotechnol 29:24–26 21221095 10.1038/nbt.1754 PMC3346182

[CR68] Robinson MD, McCarthy DJ, Smyth GK (2010) edgeR: a Bioconductor package for differential expression analysis of digital gene expression data. Bioinformatics 26:139–140 19910308 10.1093/bioinformatics/btp616 PMC2796818

[CR69] Rodríguez-Lozano DC, Piña-Medina MG, Hansberg-Pastor V, Bello-Alvarez C, Camacho-Arroyo I (2019) Testosterone promotes glioblastoma cell proliferation, migration, and invasion through androgen receptor activation. Front Endocrinol 10:16 10.3389/fendo.2019.00016 PMC6369181 30778332

[CR70] Rubin GM, Yandell MD, Wortman JR, Gabor Miklos GL, Nelson CR, Hariharan IK, Fortini ME, Li PW, Apweiler R, Fleischmann W et al (2000) Comparative genomics of the eukaryotes. Science 287:2204–2215 10731134 10.1126/science.287.5461.2204 PMC2754258

[CR71] Sanaki Y, Nagata R, Kizawa D, Léopold P, Igaki T (2020) Hyperinsulinemia drives epithelial tumorigenesis by abrogating cell competition. Dev Cell 53:379–389.e375 32386602 10.1016/j.devcel.2020.04.008

[CR72] Santabárbara-Ruiz P, Léopold P (2021) An Oatp transporter-mediated steroid sink promotes tumor-induced cachexia in *Drosophila*. Dev Cell 56:2741–2751 34610327 10.1016/j.devcel.2021.09.009

[CR73] Slemmons KK, Crose LES, Riedel S, Sushnitha M, Belyea B, Linardic CM (2017) A novel Notch-YAP circuit drives stemness and tumorigenesis in embryonal rhabdomyosarcoma. Mol Cancer Res 15:1777–1791 28923841 10.1158/1541-7786.MCR-17-0004 PMC5755394

[CR74] Subramanian A, Tamayo P, Mootha VK, Mukherjee S, Ebert BL, Gillette MA, Paulovich A, Pomeroy SL, Golub TR, Lander ES et al (2005) Gene set enrichment analysis: a knowledge-based approach for interpreting genome-wide expression profiles. Proc Natl Acad Sci USA 102:15545–15550 16199517 10.1073/pnas.0506580102 PMC1239896

[CR75] Sulli G, Rommel A, Wang XJ, Kolar MJ, Puca F, Saghatelian A, Plikus MV, Verma IM, Panda S (2018) Pharmacological activation of REV-ERBs is lethal in cancer and oncogene-induced senescence. Nature 553:351–355 29320480 10.1038/nature25170 PMC5924733

[CR76] Sullivan AA, Thummel CS (2003) Temporal profiles of nuclear receptor gene expression reveal coordinate transcriptional responses during *Drosophila* development. Mol Endocrinol 17:2125–2137 12881508 10.1210/me.2002-0430

[CR77] Tang Z, Kang B, Li C, Chen T, Zhang Z (2019) GEPIA2: an enhanced web server for large-scale expression profiling and interactive analysis. Nucleic Acids Res 47:W556–W560 31114875 10.1093/nar/gkz430 PMC6602440

[CR78] Verheyen EM (2022) The power of *Drosophila* in modeling human disease mechanisms. Dis Model Mech 15:dmm049549 10.1242/dmm.049549 PMC8990083 35348663

[CR79] Villegas SN (2019) One hundred years of Drosophila cancer research: no longer in solitude. Dis Model Mech 12:dmm039032 30952627 10.1242/dmm.039032 PMC6505481

[CR80] Wang XP, Wang H, Liu L, Li S, Emery G, Chen J (2020) Temporal coordination of collective migration and lumen formation by antagonism between two nuclear receptors. Iscience 23:101335 10.1016/j.isci.2020.101335 PMC7366032 32682323

[CR81] Weghorn D, Sunyaev S (2017) Bayesian inference of negative and positive selection in human cancers. Nat Genet 49:1785–1788 29106416 10.1038/ng.3987

[CR82] Weng AP, Millholland JM, Yashiro-Ohtani Y, Arcangeli ML, Lau A, Wai C, Bianco C, Rodriguez CG, Sai H, Tobias J et al (2006) c-Myc is an important direct target of Notch1 in T-cell acute lymphoblastic leukemia/lymphoma. Gene Dev 20:2096–2109 16847353 10.1101/gad.1450406 PMC1536060

[CR83] White KP, Hurban P, Watanabe T, Hogness DS (1997) Coordination of *Drosophila* metamorphosis by two ecdysone-induced nuclear receptors. Science 276:114–117 9082981 10.1126/science.276.5309.114

[CR84] Xu H, Zhang JP, Zheng XN, Tan P, Xiong XY, Yi XYL, Yang Y, Wang Y, Liao DZ, Li H, et al (2022) SR9009 inhibits lethal prostate cancer subtype 1 by regulating the LXRα/FOXM1 pathway independently of REV-ERBs. Cell Death Dis 13:949 10.1038/s41419-022-05392-6 PMC9649669 36357378

[CR85] Xu T, Wang W, Zhang S, Stewart RA, Yu W (1995) Identifying tumor suppressors in genetic mosaics: the Drosophila lats gene encodes a putative protein kinase. Development 121:1053–1063 7743921 10.1242/dev.121.4.1053

[CR86] Xu YC, Huangyang PW, Wang Y, Xue LR, Devericks E, Nguyen HG, Yu XY, Oses-Prieto JA, Burlingame AL, Miglani S et al (2021) ERα is an RNA-binding protein sustaining tumor cell survival and drug resistance. Cell 184:5215–5229 34559986 10.1016/j.cell.2021.08.036 PMC8547373

[CR87] Yu G, Wang LG, Han Y, He QY (2012) clusterProfiler: an R package for comparing biological themes among gene clusters. OMICS 16:284–287 22455463 10.1089/omi.2011.0118 PMC3339379

[CR88] Yu G, Wang LG, He QY (2015) ChIPseeker: an R/Bioconductor package for ChIP peak annotation, comparison and visualization. Bioinformatics 31:2382–2383 25765347 10.1093/bioinformatics/btv145

[CR89] Yu M, Li W, Wang Q, Wang Y, Lu F (2018) Circadian regulator NR1D2 regulates glioblastoma cell proliferation and motility. Oncogene 37:4838–4853 29773903 10.1038/s41388-018-0319-8

[CR90] Zhang L, Ren FF, Zhang Q, Chen YB, Wang B, Jiang J (2008a) The TEAD/TEF family of transcription factor scalloped mediates hippo signaling in organ size control. Dev Cell 14:377–387 18258485 10.1016/j.devcel.2008.01.006 PMC2292673

[CR91] Zhang Y, Liu T, Meyer CA, Eeckhoute J, Johnson DS, Bernstein BE, Nusbaum C, Myers RM, Brown M, Li W et al (2008b) Model-based analysis of ChIP-Seq (MACS). Genome Biol 9:R137 18798982 10.1186/gb-2008-9-9-r137 PMC2592715

[CR92] Zheng JD, Guo YF, Shi CY, Yang S, Xu WY, Ma XJ (2023) Differential Ire1 determines loser cell fate in tumor-suppressive cell competition. Cell Rep 42:113303 10.1016/j.celrep.2023.113303 37924514

[CR93] Zheng YG, Pan DJ (2019) The Hippo signaling pathway in development and disease. Dev Cell 50:264–282 31386861 10.1016/j.devcel.2019.06.003 PMC6748048

[CR94] Zhou BH, Lin WL, Long YL, Yang YK, Zhang H, Wu KM, Chu Q (2022) Notch signaling pathway: architecture, disease, and therapeutics. Signal Transduct Target Ther 7:95 35332121 10.1038/s41392-022-00934-y PMC8948217

[CR95] Zipper L, Jassmann D, Burgmer S, Görlich B, Reiff T (2020) Ecdysone steroid hormone remote controls intestinal stem cell fate decisions via the PPARγ-homolog Eip75B in *Drosophila*. Elife 9:e55795 32773037 10.7554/eLife.55795 PMC7440922

