# Supplementary material for: Nuclear receptor E75/NR1D2 promotes tumor malignant transformation by integrating Hippo and Notch pathways
Source: EMBO J. 2024 Nov 8;43(24):6336–63. doi: 10.1038/s44318-024-00290-3 (PMC11649922; doi:10.1038/s44318-024-00290-3)
Supplement: Supplementary file 2 — Dataset EV1 [file 44318_2024_290_MOESM2_ESM.docx]

**Dataset EV1: Detailed genotypes for each figure.**

A complete list of detailed genotypes used in this study is provided below, corresponding to Figures 1 through 5 and Figures EV1 through EV5. The detailed genotypes for each experiment are as follows:

| **Figure** | **Genotype** |
| --- | --- |
| **Fig 1**. |  |
| **Fig 1B** | ey-Flp1/+; act>y+>GAL4, UAS-GFP/+; Tub-Gal80, FRT 82B/FRT 82B |
|  | ey-Flp1/+; act>y+>GAL4, UAS-GFP/+; Tub-Gal80, FRT 82B/FRT 82B, scrib^1^ |
|  | ey-Flp1/+; act>y+>GAL4, UAS-GFP/+; Tub-Gal80, FRT 82B/FRT 82B, wts^x1^ |
|  | ey-Flp1/+; act>y+>GAL4, UAS-GFP/+; Tub-Gal80, FRT 82B/FRT 82B, scrib^1^, wts^x1^ |
|  | ey-Flp1/+; act>y+>GAL4, UAS-GFP/+; Tub-Gal80, FRT 82B/FRT 82B, UAS-Ras^V12^ |
|  | ey-Flp1/+; act>y+>GAL4, UAS-GFP/+; Tub-Gal80, FRT 82B/FRT 82B, scrib^1^, Ras^V12^ |
|  |  |
| **Fig 1F** | ey-Flp1/+; act>y+>GAL4, UAS-GFP/EcRE-lacZ; Tub-Gal80, FRT 82B/FRT 82B |
|  | ey-Flp1/+; act>y+>GAL4, UAS-GFP/EcRE-lacZ; Tub-Gal80, FRT 82B/FRT 82B/FRT 82B, scrib^1^, wts^x1^ |
|  | ey-Flp1/+; act>y+>GAL4, UAS-GFP//EcRE-lacZ; Tub-Gal80, FRT 82/FRT 82B, scrib^1^, Ras^V12^ |
|  |  |
| **Fig 1G** | ey-Flp1/+; act>y+>GAL4, UAS-GFP/+; Tub-Gal80, FRT 82B/FRT 82B |
|  | ey-Flp1/+; act>y+>GAL4, UAS-GFP/+; Tub-Gal80, FRT 82B/FRT 82B, scrib^1^, wts^x1^ |
|  | ey-Flp1/+; act>y+>GAL4, UAS-GFP/+; Tub-Gal80, FRT 82B/FRT 82B, scrib^1^, Ras^V12^ |
|  |  |
| **Fig 1H** | ey-Flp1/+; act>y+>GAL4, UAS-GFP/+; Tub-Gal80, FRT 82B/FRT 82B, scrib^1^,wts^x1^ |
|  | ey-Flp1/+; act>y+>GAL4, UAS-GFP/+; Tub-Gal80, FRT 82B/FRT 82B, UAS-Hr3, scrib^1^,wts^x1^ |
|  | ey-Flp1/+; act>y+>GAL4, UAS-GFP/+; Tub-Gal80, FRT 82B/FRT 82B, UAS-Ftz-f1, scrib^1^,wts^x1^ |
|  | ey-Flp1/+; act>y+>GAL4, UAS-GFP/+; Tub-Gal80, FRT 82B/FRT 82B, UAS-Eip93F, scrib^1^,wts^x1^ |
|  | ey-Flp1/+; act>y+>GAL4, UAS-GFP/UAS-Br-Z1; Tub-Gal80, FRT 82B/FRT 82B, scrib^1^,wts^x1^ |
|  | ey-Flp1/+; act>y+>GAL4, UAS-GFP/ UAS-E75^Flag^; Tub-Gal80, FRT 82B/FRT 82B, scrib^1^,wts^x1^ |
|  |  |
| **Fig EV1D** | ey-Flp1; Tub-Gal80,FRT 40A/FRT 40A; Act>y+>Gal4, UAS-GFP/+ |
|  | ey-Flp1; Tub-Gal80,FRT 40A/FRT 40A, UAS-Ras^V12^; Act>y+>Gal4, UAS-GFP/+ |
|  | ey-Flp1; Tub-Gal80,FRT 40A/FRT 40A, lgl^4^,Ras^V12^; Act>y+>Gal4, UAS-GFP/+ |
|  |  |
| **Fig EV1E** | ey-Flp1; Tub-Gal80,FRT 40A/FRT 40A, lgl^4^,Ras^V12^; Act>y+>Gal4, UAS-GFP/+ |
|  | ey-Flp1; Tub-Gal80,FRT 40A/FRT 40A, lgl^4^,Ras^V12^; Act>y+>Gal4, UAS-GFP/UAS-EcRA |
|  | ey-Flp1; Tub-Gal80,FRT 40A/FRT 40A, lgl^4^,Ras^V12^; Act>y+>Gal4, UAS-GFP/UAS-EcRB1 |
|  | ey-Flp1; Tub-Gal80,FRT 40A/FRT 40A, lgl^4^,Ras^V12^; Act>y+>Gal4, UAS-GFP/UAS-EcRC |
|  |  |
| **Fig EV1F** | ey-Flp1; Tub-Gal80,FRT 40A/FRT 40; Act>y+>Gal4, UAS-GFP/+ |
|  | ey-Flp1; Tub-Gal80,FRT 40A/FRT 40; Act>y+>Gal4, UAS-GFP/UAS-EcRA |
|  | ey-Flp1; Tub-Gal80,FRT 40A/FRT 40; Act>y+>Gal4, UAS-GFP/UAS-EcRB1 |
|  | ey-Flp1; Tub-Gal80,FRT 40A/FRT 40; Act>y+>Gal4, UAS-GFP/UAS-EcRC |
|  |  |
| **FigEV1G** | ey-Flp1; Tub-Gal80,FRT 40A/FRT 40A, UAS-E75^Flag^; Act>y+>Gal4, UAS-GFP/+ |
|  | ey-Flp1; Tub-Gal80,FRT 40A/FRT 40A, UAS-E75^Flag^; Act>y+>Gal4, UAS-GFP/ UAS-EcRA |
|  |  |
| **FigEV1H** | ey-Flp1; Tub-Gal80,FRT 40A/FRT 40A, lgl^4^,Ras^V12^; Act>y+>Gal4, UAS-GFP/+ |
|  | ey-Flp1; Tub-Gal80,FRT 40A/FRT 40A, lgl^4^,Ras^V12^; Act>y+>Gal4, UAS-GFP/UAS-EcRA |
|  | ey-Flp1; Tub-Gal80,FRT 40A/FRT 40A, lgl^4^,Ras^V12^; Act>y+>Gal4, UAS-GFP/UAS-EcRB1 |
|  | ey-Flp1; Tub-Gal80,FRT 40A/FRT 40A, lgl^4^,Ras^V12^; Act>y+>Gal4, UAS-GFP/UAS-EcRC |
|  |  |
| **FigEV1I** | ey-Flp1/+; act>y+>GAL4, UAS-GFP/+; Tub-Gal80, FRT 82B/ FRT 82B, EcRE-lacZ |
|  | ey-Flp1/+; act>y+>GAL4, UAS-GFP/UAS-E75^Falg^; Tub-Gal80, FRT 82B/ FRT 82B, EcRE-lacZ |
|  |  |
| **FigEV1J** | ey-Flp1/+; act>y+>GAL4, UAS-GFP/+; Tub-Gal80, FRT 82B/ FRT 82B |
|  | ey-Flp1/+; act>y+>GAL4, UAS-GFP/UAS-E75^Falg^; Tub-Gal80, FRT 82B/ FRT 82B |
|  |  |
| **FigEV1L** | ey-Flp1/+; act>y+>GAL4, UAS-GFP/+; Tub-Gal80, FRT 82B/FRT 82B |
|  | ey-Flp1/+; act>y+>GAL4, UAS-GFP/UAS-E75 ^Flag^; Tub-Gal80, FRT 82B/FRT 82B |
|  | ey-Flp1/+; act>y+>GAL4, UAS-GFP/+; Tub-Gal80, FRT 82B/FRT 82B, UAS-Ras^V12^ |
|  | ey-Flp1/+; act>y+>GAL4, UAS-GFP/ UAS-E75^Flag^; Tub-Gal80, FRT 82B/FRT 82B, UAS-Ras^V12^ |
|  | ey-Flp1/+; act>y+>GAL4, UAS-GFP/+; Tub-Gal80, FRT 82B/FRT 82B, Raf^GOF^, scrib^1^ |
|  | ey-Flp1/+; act>y+>GAL4, UAS-GFP/ UAS-E75^Flag^; Tub-Gal80, FRT 82B/FRT 82B, Raf^GOF^, scrib^1^ |
| **Fig 2.** |  |
| **Fig 2A** | ey-Flp1/+; act>y+>GAL4, UAS-GFP/+; Tub-Gal80, FRT 82B/FRT 82B |
|  | ey-Flp1/+; act>y+>GAL4, UAS-GFP/UAS-E75A^Falg^; Tub-Gal80, FRT 82B/FRT 82B |
|  |  |
| **Fig 2B** | ey-Flp1/+; act>y+>GAL4, UAS-GFP/+; Tub-Gal80, FRT 82B/FRT 82B |
|  | ey-Flp1/+; act>y+>GAL4, UAS-GFP/UAS-E75A^Falg^; Tub-Gal80, FRT 82B/FRT 82B |
|  | ey-Flp1/+; act>y+>GAL4, UAS-GFP/+; Tub-Gal80, FRT 82B/FRT 82B, scrib^1^ |
|  | ey-Flp1/+; act>y+>GAL4, UAS-GFP/UAS-E75A^Flag^; Tub-Gal80, FRT 82B/FRT 82B, scrib^1^ |
|  | ey-Flp1/+; act>y+>GAL4, UAS-GFP/+; Tub-Gal80, FRT 82B/FRT 82B, wts^x1^ |
|  | ey-Flp1/+; act>y+>GAL4, UAS-GFP/UAS-E75A^Flag^; Tub-Gal80, FRT 82B/FRT 82B, wts^x1^ |
|  | ey-Flp1/+; act>y+>GAL4, UAS-GFP/+; Tub-Gal80, FRT 82B/FRT 82B, scrib^1^,wts^x1^ |
|  | ey-Flp1/+; act>y+>GAL4, UAS-GFP/UAS-E75A^Flag^; Tub-Gal80, FRT 82B/FRT 82B, scrib^1^,wts^x1^ |
|  |  |
| **Fig 2C** | ey-Flp1/+; act>y+>GAL4, UAS-GFP/+; Tub-Gal80, FRT 82B/FRT 82B |
|  | ey-Flp1/+; act>y+>GAL4, UAS-GFP/+; Tub-Gal80, FRT 82B/FRT 82B, scrib^1^,wts^x1^ |
|  | ey-Flp1/+; act>y+>GAL4, UAS-GFP/UAS-E75A^Falg^; Tub-Gal80, FRT 82B/FRT 82B |
|  | ey-Flp1/+; act>y+>GAL4, UAS-GFP/UAS-E75A^Flag^; Tub-Gal80, FRT 82B/FRT 82B, scrib^1^,wts^x1^ |
|  |  |
| **Fig 2D** | ey-Flp1/+; act>y+>GAL4, UAS-GFP/+; Tub-Gal80, FRT 82B/FRT 82B, scrib^1^,wts^x1^ |
|  | ey-Flp1/+; act>y+>GAL4, UAS-GFP/UAS-E75A^Flag^; Tub-Gal80, FRT 82B/FRT 82B, scrib^1^,wts^x1^ |
|  |  |
| **Fig 2E** | ey-Flp1/+; act>y+>GAL4, UAS-GFP/+; Tub-Gal80, FRT 82B/FRT 82B, scrib^1^,wts^x1^ |
|  | ey-Flp1/+; act>y+>GAL4, UAS-GFP/UAS-E75A^Flag^; Tub-Gal80, FRT 82B/FRT 82B, scrib^1^,wts^x1^ |
|  |  |
| **Fig EV2A** | ey-Flp1/+; act>y+>GAL4, UAS-GFP/+; Tub-Gal80, FRT 82B/FRT 82B, scrib^1^,wts^x1^ |
|  | ey-Flp1/+; act>y+>GAL4, UAS-GFP/UAS-E75A^Flag^; Tub-Gal80, FRT 82B/FRT 82B, scrib^1^,wts^x1^ |
|  |  |
| **Fig EV2D** | nub-GAL4, UAS-GFP/+ |
|  | nub-GAL4, UAS-GFP/UAS-Dcer,UAS-E75-IR |
|  | GMR-GAL4/+ |
|  | GMR-GAL4/UAS-E75-IR |
|  |  |
| **Fig EV2H** | ptc-GAL4, UAS-GFP/+ |
|  | ptc-GAL4, UAS-GFP/UAS-wts |
|  | ptc-GAL4, UAS-GFP/UAS-E75A^Falg^ |
|  | ptc-GAL4, UAS-GFP/UAS-wts, UAS-E75A^Falg^ |
|  | ptc-GAL4, UAS-GFP/UAS-Yki-IR |
|  | ptc-GAL4, UAS-GFP/UAS-Yki-IR,UAS-E75A^Flag^ |
|  |  |
| **Fig EV2I** | ey-Flp5, Act>y+>Gal4, UAS-GFP/UAS-E75A^Flag^; Diap1-LacZ, FRT 82B, Tub-Gal80/ FRT 82B |
|  | ey-Flp5, Act>y+>Gal4, UAS-GFP/ UAS-E75A^Flag^; Diap1-LacZ, FRT 82B, Tub-Gal80/ FRT 82B |
|  | ey-Flp5, Act>y+>Gal4, UAS-GFP/UAS-E75A^Flag^; Diap1-LacZ, FRT 82B, Tub-Gal80/ FRT 82B, UAS-Sd IR |
|  |  |
| **Fig EV2J** | FRT 42D, Tub-Gal80/ FRT 42D; ey-Flp6, act>y+>Gal4, UAS-GFP/+ |
|  | FRT 42D, Tub-Gal80/ FRT 42D, Yki^B5^; ey-Flp6, act>y+>Gal4, UAS-GFP/+ |
|  |  |
| **Fig EV2K** | ey-Flp1/+; act>y+>GAL4, UAS-GFP/UAS-E75A^Flag^; Tub-Gal80, FRT 82B/FRT 82B, scrib^1^ |
|  | ey-Flp1/+; act>y+>GAL4, UAS-GFP/UAS-E75A^Flag^; Tub-Gal80, FRT 82B/FRT 82B, scrib^1^, UAS-Sd IR |
| **Fig 3.** |  |
| **Fig 3A** | ey-Flp1/+; act>y+>GAL4, UAS-GFP/+; Tub-Gal80, FRT 82B/FRT 82B, scrib^1^,wts^x1^ |
|  | ey-Flp1/+; act>y+>GAL4, UAS-GFP/UAS-E75A^Flag^; Tub-Gal80, FRT 82B/FRT 82B, scrib^1^,wts^x1^ |
|  |  |
| **Fig 3B** | ey-Flp5, Act>y+>Gal4, UAS-GFP/UAS-E75A^Flag^; Diap1-LacZ, FRT 82B, Tub-Gal80/ FRT 82B |
|  | ey-Flp5, Act>y+>Gal4, UAS-GFP/+; Diap1-LacZ, FRT 82B, Tub-Gal80/ FRT 82B, scrib^1^,wts^x1^  ey-Flp5, Act>y+>Gal4, UAS-GFP/UAS-E75A^Flag^; Diap1-LacZ, FRT 82B,Tub-Gal80/ FRT 82B, scrib^1^,wts^x1^ |
|  | ey-Flp1/+; act>y+>GAL4, UAS-GFP/UAS-E75A^Flag^, ex^e1^; Tub-Gal80, FRT 82B/FRT 82B |
|  | ey-Flp1/+; act>y+>GAL4, UAS-GFP/ex^e1^; Tub-Gal80, FRT 82B/FRT 82B, scrib^1^,wts^x1^ |
|  | ey-Flp1/+; act>y+>GAL4, UAS-GFP/ UAS-E75A^Flag^, ex^e1^; Tub-Gal80, FRT 82B/FRT 82B, scrib^1^,wts^x1^ |
|  |  |
| **Fig 3C** | ey-Flp1/+; act>y+>GAL4, UAS-GFP/+; Tub-Gal80, FRT 82B/FRT 82B |
|  | ey-Flp1/+; act>y+>GAL4, UAS-GFP/UAS-Dcer; Tub-Gal80, FRT 82B/FRT 82B, UAS-E75-IR |
|  | ey-Flp1/+; act>y+>GAL4, UAS-GFP/UAS-E75A^Falg^; Tub-Gal80, FRT 82B/FRT 82B |
|  | ey-Flp1/+; act>y+>GAL4, UAS-GFP/+; Tub-Gal80, FRT 82B/FRT 82B scrib^1^,wts^x1^ |
|  | ey-Flp1/+; act>y+>GAL4, UAS-GFP/UAS-Dcer, UAS-E75-IR; Tub-Gal80, FRT 82B/FRT 82B, scrib^1^,wts^x1^ |
|  |  |
| **Fig 3D** | ptc-GAL4, UAS-GFP/+; ban-lacZ/+ |
|  | ptc-GAL4, UAS-GFP/UAS-E75A^Falg^; ban-lacZ/+ |
|  | en-GAL4, UAS-GFP/UAS-Dcer; ban-lacZ/UAS-E75-IR |
|  | ptc-GAL4, UAS-GFP/+; Diap1-lacZ/+ |
|  | ptc-GAL4, UAS-GFP/UAS-E75A^Falg^; Diap1-lacZ/+ |
|  | en-GAL4, UAS-GFP/UAS-Dcr2; Diap1-lacZ/UAS-E75-IR |
|  | ptc-GAL4, UAS-GFP/ex^e1^ |
|  | ptc-GAL4, UAS-GFP/UAS- E75A^Falg^, ex^e1^ |
|  | hh-GAL4, UAS-GFP/ex^e1^; UAS-E75-IR/+ |
|  | ptc-GAL4, UAS-GFP/+ |
|  | ptc-GAL4, UAS-GFP/UAS-E75A^Falg^ |
|  | hh-GAL4, UAS-GFP/UAS-Dcer; UAS-E75-IR/+ |
|  |  |
| **Fig 3E** | ey-Flp1/+; act>y+>GAL4, UAS-GFP/, UAS-E75A^Flag^; Tub-Gal80, FRT 82B/FRT 82B, UAS-Ras^V12^ |
|  | ey-Flp1/+; act>y+>GAL4, UAS-GFP/Yki^B5^,UAS-E75A^Flag,^; Tub-Gal80,FRT 82B/FRT 82B, UAS-Ras^V12^ |
|  | ey-Flp1/+; act>y+>GAL4, UAS-GFP/ UAS-wts,UAS-E75A^Flag,^; Tub-Gal80, FRT 82B/FRT 82B, UAS-Ras^V12^ |
|  | ey-Flp1/+; act>y+>GAL4, UAS-GFP/UAS-E75A^Flag^; Tub-Gal80, FRT 82B/FRT 82B, scrib^1^,wts^x1^ |
|  | ey-Flp1/+; act>y+>GAL4, UAS-GFP/UAS-wts,UAS-E75A^Flag^;Tub-Gal80,FRT82B/FRT82B, scrib^1^,wts^x1^ |
|  |  |
| **Fig 3F** | nub-GAL4, UAS-GFP/+ |
|  | nub-GAL4, UAS-GFP/UAS-Dcer,UAS-E75-IR |
|  | nub-GAL4, UAS-GFP/UAS-Yki-IR |
|  | nub-GAL4, UAS-GFP/+; UAS-Yki/+ |
|  | nub-GAL4, UAS-GFP/ UAS-E75-IR; UAS-Yki/+ |
|  |  |
| **Fig EV3D** | ey-Flp1/+; act>y+>GAL4, UAS-GFP/+; Tub-Gal80, FRT 82B/FRT 82B |
|  | ey-Flp1/+; act>y+>GAL4, UAS-GFP/UAS-E75-IR; Tub-Gal80, FRT 82B/FRT 82B |
|  | ey-Flp1/+; act>y+>GAL4, UAS-GFP/UAS-E75A^Flag^; Tub-Gal80, FRT 82B/FRT 82B |
|  | ey-Flp1/+; act>y+>GAL4, UAS-GFP/UAS-E75A^Flag^; Tub-Gal80, FRT 82B/FRT 82B, scrib^1^ |
|  | ey-Flp1/+; act>y+>GAL4, UAS-GFP/UAS-E75A^Flag^; Tub-Gal80, FRT 82B/FRT 82B, wts^x1^ |
|  | ey-Flp1/+; act>y+>GAL4, UAS-GFP/UAS-E75A^Flag^; Tub-Gal80, FRT 82B/FRT 82B, scrib^1^,wts^x1^ |
|  |  |
| **Fig EV3E** | ey-Flp1/+; act>y+>GAL4, UAS-GFP/ E(spl)mβ-HLH-lacZ,UAS-E75A^Flag^; Tub-Gal80, FRT 82B/FRT 82B, scrib^1^ |
|  | ey-Flp1/+; act>y+>GAL4, UAS-GFP/ E(spl)mβ-HLH-lacZ,UAS-E75A^Flag^; Tub-Gal80, FRT 82B/FRT 82B, wts^x1^ |
|  | ey-Flp1/+; act>y+>GAL4, UAS-GFP/ E(spl)mβ-HLH-lacZ,UAS-E75A^Flag^; Tub-Gal80, FRT 82B/FRT 82B, scrib^1^, wts^x1^ |
|  |  |
| **Fig EV3F** | hh-GAL4, UAS-GFP/+ |
|  | hh-GAL4, UAS-GFP/UAS-E75-IR |
|  |  |
| **Fig EV3G** | ey-Flp1/+; act>y+>GAL4, UAS-GFP/+; Tub-Gal80, FRT 82B/FRT 82B |
|  | ey-Flp1/+; act>y+>GAL4, UAS-GFP/UAS-Notch^DN^; Tub-Gal80, FRT 82B/FRT 82B |
|  |  |
| **Fig EV3H** | ey-Flp1/+; act>y+>GAL4, UAS-GFP/UAS-Notch^act^; Tub-Gal80, FRT 82B/FRT 82B |
|  | ey-Flp1/+; act>y+>GAL4, UAS-GFP/UAS- Notch^act^, UAS-E75-IR; Tub-Gal80, FRT 82B/FRT 82B |
|  |  |
| **Fig EV3I** | myc-lacZ/+; ptc-GAL4,UAS-GFP/+ |
|  | myc-lacZ/+; ptc-Gal4,UAS-GFP/UAS-E75-IR |
|  |  |
| **Fig EV3K** | ptc-GAL4, UAS-GFP/+ |
|  | ptc-GAL4, UAS-GFP/UAS-E75A^Flag^ |
|  | ptc-GAL4, UAS-GFP/UAS-Myc-IR |
|  | ptc-GAL4, UAS-GFP/UAS-E75A^Flag^, UAS-Myc-IR |
|  |  |
| **Fig EV3L** | ey-Flp1/+; act>y+>GAL4, UAS-GFP/+; Tub-Gal80, FRT 82B/FRT 82B |
|  | ey-Flp1/+; act>y+>GAL4, UAS-GFP/UAS-E75; Tub-Gal80, FRT 82B/FRT 82B |
|  | ey-Flp1/+; act>y+>GAL4, UAS-GFP/UAS-Myc-IR; Tub-Gal80, FRT 82B/FRT 82B |
|  | ey-Flp1/+; act>y+>GAL4, UAS-GFP/ UAS-E75, UAS-Myc-IR; Tub-Gal80, FRT 82B/FRT 82B |
|  |  |
| **Fig 4.** |  |
| **Fig 4B** | hh-GAL4, UAS-mCherry/UAS-E75A^Flag^; E(spl)mβ-HLH-lacZ/Tub-Gal80^ts^ |
|  | hh-GAL4, UAS-GFP/UAS-Dcer; E(spl)mβ-HLH-lacZ/UAS-E75-IR |
|  | hh-GAL4, UAS-mCherry/+; NRE-GFP/+ |
|  | hh-GAL4, UAS-mCherry/UAS-E75A^Flag^; NRE-GFP/Tub-Gal80^ts^ |
|  |  |
| **Fig 4C** | ey-Flp1/+; act>y+>GAL4, UAS-GFP/+; Tub-Gal80, FRT 82B/FRT 82B, E(spl)mβ-HLH-lacZ |
|  | ey-Flp1/+; act>y+>GAL4, UAS-GFP/UAS-E75A^Falg^; Tub-Gal80, FRT 82B/FRT 82B, E(spl)mβ-HLH-lacZ |
|  |  |
| **Fig 4D** | ey-Flp1/+; act>y+>GAL4, UAS-GFP/+; Tub-Gal80, FRT 82B/FRT 82B, scrib^1^ |
|  | ey-Flp1/+; act>y+>GAL4, UAS-GFP/UAS-Notch^DN^,UAS-E75A^Flag^; Tub-Gal80, FRT 82B/FRT 82B, scrib^1^ |
|  | ey-Flp1/+; act>y+>GAL4, UAS-GFP/+; Tub-Gal80, FRT 82B/FRT 82B, wts^x1^ |
|  | ey-Flp1/+; act>y+>GAL4, UAS-GFP/ UAS-Notch^DN^,UAS-E75A^Flag^; Tub-Gal80, FRT 82B/FRT 82B,wts^x1^ |
|  | ey-Flp1/+; act>y+>GAL4, UAS-GFP/+; Tub-Gal80, FRT 82B/FRT 82B, scrib^1^,wts^x1^ |
|  | ey-Flp1/+; act>y+>GAL4, UAS-GFP/ UAS-Notch^DN^, UAS-E75A^Flag^; Tub-Gal80, FRT 82B/FRT 82B, scrib^1^,wts^x1^ |
|  |  |
| **Fig 4E,F** | ey-Flp1/+; act>y+>GAL4, UAS-GFP/UAS-Notch^act^; Tub-Gal80, FRT 82B/FRT 82B |
|  | ey-Flp1/+; act>y+>GAL4, UAS-GFP/UAS-Notch^act^; Tub-Gal80, FRT 82B/FRT 82B, scrib^1^ |
|  | ey-Flp1/+; act>y+>GAL4, UAS-GFP/UAS-Notch^act^; Tub-Gal80, FRT 82B/FRT 82B, wts^x1^ |
|  | ey-Flp1/+; act>y+>GAL4, UAS-GFP/UAS-Notch^act^; Tub-Gal80, FRT 82B/FRT 82B, scrib^1^,wts^x1^ |
| **Fig 4G** | myc-LacZ/+; ptc-GAL4, UAS-GFP/+ |
|  | myc-lacZ/+; ptc-GAL4, UAS-GFP/UAS-E75A^Falg^ |
|  | myc-lacZ/+; ptc-GAL4, UAS-GFP/UAS-Yki-IR, UAS-E75A^Falg^ |
|  | myc-lacZ/+; ptc-GAL4, UAS-GFP/UAS-Notch-IR, UAS-E75A^Falg^ |
|  |  |
| **Fig 4H** | ey-Flp1/+; act>y+>GAL4, UAS-GFP/+; Tub-Gal80, FRT 82B/FRT 82B |
|  | ey-Flp1/+; act>y+>GAL4, UAS-GFP/UAS-E75A^Flag^; Tub-Gal80, FRT 82B/FRT82B |
|  |  |
| **Fig 4I** | ey-Flp1/+; act>y+>GAL4, UAS-GFP/UAS-E75A^Flag^; Tub-Gal80, FRT 82B/FRT 82B, scrib^1^,wts^x1^ |
|  | ey-Flp1/+; act>y+>GAL4, UAS-GFP/UAS-E75A^Flag^, UAS-Myc-IR; Tub-Gal80, FRT 82B/FRT 82B, scrib^1^,wts^x1^ |
|  |  |
| **Fig EV4A** | ey-Flp1/+; act>y+>GAL4, UAS-GFP/UAS-E75A^Flag^, UAS-Sd^HA^; Tub-Gal80, FRT 82B/FRT 82B, UAS-Yki |
|  |  |
| **Fig EV4B** | nub-GAL4, UAS-GFP/UAS-NICD; UAS-Yki/+ |
|  | nub-GAL4, UAS-GFP/UAS-NICD; UAS-Yki, UAS-E75A^Flag^/+ |
|  |  |
| **Fig EV4C** | ey-Flp1/+; act>y+>GAL4, UAS-GFP/+; Tub-Gal80, FRT 82B/FRT 82B |
|  |  |
| **Fig 5.** |  |
| **Fig 5A** | nub-GAL4, UAS-GFP/UAS-Yki^myc^, UAS-E75A^Flag^ |
|  |  |
| **Fig 5B** | nub-GAL4, UAS-GFP/UAS-Sd^HA^ , UAS-E75A^Flag^ |
|  |  |
| **Fig 5C** | ey-Flp1/+; act>y+>GAL4, UAS-GFP/UAS-E75A^Flag^; Tub-Gal80, FRT 82B/FRT 82B |
|  |  |
| **Fig 5D,E** | ey-Flp1/+; act>y+>GAL4, UAS-GFP/, UAS-E75A^Flag^, UAS-Sd^HA^; Tub-Gal80, FRT 82B/FRT 82B, UAS-Yki |
|  |  |
| **Fig 5F,G** | ey-Flp1/+; act>y+>GAL4, UAS-GFP/UAS-E75A^Flag^, UAS-Notch^act^; Tub-Gal80, FRT 82B/FRT 82B, UAS-Yki |
|  |  |
| **Fig 5H** | ey-Flp1/+; act>y+>GAL4, UAS-GFP/UAS-E75A^Flag^; Tub-Gal80, FRT 82B/FRT 82B |
|  |  |
| **Fig 5I** | ey-Flp1/+; act>y+>GAL4, UAS-GFP/UAS-Notch^act^; Tub-Gal80, FRT 82B/FRT 82B  ey-Flp1/+; act>y+>GAL4, UAS-GFP/UAS-Notch^act^, UAS-Yki^myc^; Tub-Gal80, FRT 82B/FRT 82B |
|  | ey-Flp1/+; act>y+>GAL4, UAS-GFP/UAS-Yki^myc^; Tub-Gal80, FRT 82B/FRT 82B |
|  | ey-Flp1/+; act>y+>GAL4, UAS-GFP/UAS-Notch^act^, UAS-Yki^myc^, UAS-E75A^Flag^; Tub-Gal80, FRT 82B/FRT 82B |
|  |  |
| **Fig 5J** | ey-Flp1/+; act>y+>GAL4, UAS-GFP/UAS-Yki^myc^; Tub-Gal80, FRT 82B/FRT 82B |
|  | ey-Flp1/+; act>y+>GAL4, UAS-GFP/UAS-Notch^act^, UAS-Yki^myc^,UAS-E75A^Flag^; Tub-Gal80, FRT 82B/FRT 82B |
