# Supplementary material for: Nuclear receptor E75/NR1D2 promotes tumor malignant transformation by integrating Hippo and Notch pathways
Source: EMBO J. 2024 Nov 8;43(24):6336–63. doi: 10.1038/s44318-024-00290-3 (PMC11649922; doi:10.1038/s44318-024-00290-3)
Supplement: Supplementary file 9 — Expanded View Figures [file 44318_2024_290_MOESM9_ESM.pdf]

Expanded View Figures

Figure EV1. Ecdysone signaling inhibition promotes tumor malignancy.

(**A**) Quantification of the relative size of GFP positive regions in Fig. 1B ($n = 6, 10, 9, 5, 8, 6$). *n* represents the number of biological replicates. Statistical analysis by ordinary one-way ANOVA test; mean ± SD. ***$p = 0.0004$, *$p = 0.0378$. (**B**) Volcano plot of differentially expressed genes (DEGs) in *scrib*$^{-/-}$,*wts*$^{-/-}$ and *scrib*$^{-/-}$,*Ras*^{v12} tumors (top panel). Log2 fold-change of *E75* expression in *scrib*$^{-/-}$,*Ras*^{v12} vs *scrib*$^{-/-}$,*wts*$^{-/-}$ tumors (bottom panel). (**C**) GSEA enrichment of ecdysone-related genes in *scrib*$^{-/-}$,*wts*$^{-/-}$ and *scrib*$^{-/-}$,*Ras*^{v12} tumors. The FWER $p < 0.05$ served as the significance threshold. (**D**) Confocal images of eye-antennal discs bearing *ey-Flp*-MARCM-induced mosaics of each genotype stained with Broad (Br) antibody. (**D′**) Quantification of relative Br intensity of GFP positive mosaics clones ($n = 6, 7, 8$). *n* represents the number of biological replicates. Statistical analysis by ordinary one-way ANOVA test; mean + SD. ****$p = 0.0000000006$, ***$p = 0.00045$. (**E**) Dorsal views of *ey-Flp*-MARCM-induced GFP-positive tumor-bearing larvae and the corresponding dissected tumor (right). Quantification of pupation rate of tumor-bearing larvae (**E′**) and relative tumor size of GFP positive mosaics clones (**E″**, $n = 9, 4, 8, 6$). *n* represents the number of biological replicates. (**E″**) Statistical analysis by ordinary one-way ANOVA test; mean ± SD. *$p = 0.0222$, ****$p = 0.000095$, **$p = 0.0014$. (**F**, **G**) Confocal images of eye-antennal discs bearing *ey-Flp*-MARCM-induced mosaics of each genotype. (**F′**, **G′**) Quantification of the relative size of GFP positive regions (**F′**, $n = 8, 5, 6, 5$; **G′**, $n = 9, 9$). *n* represents the number of biological replicates. (**F′**) Statistical analysis by ordinary one-way ANOVA test; mean ± SD. ns non-significant, $p = 0.1748$. (**G′**) Statistical analysis by students' *t*-test; mean ± SD. ***$p = 0.0001$. (**H**) Confocal images of *ey-Flp*-MARCM-induced tumor of each genotype stained with Broad (Br) antibody. (**H′**) Quantification of relative Br intensity of GFP positive mosaics clones ($n = 7, 5, 6, 6$). *n* represents the number of biological replicates. Statistical analysis by ordinary one-way ANOVA test; mean ± SD. ****$p = 0.000007$, ****$p = 0.0000009$, ****$p = 0.00000069$. (**I**, **J**) Confocal images of eye-antennal discs bearing *ey-Flp*-MARCM-induced mosaics of wild-type and E75 overexpression stained with anti-β-galactosidase antibody for the *EcRE-LacZ* staining (**I**), anti-Br antibody (**J**). Clones are circled by the white dashed line. (**K**) Schematic diagram of ecdysone signaling, ecdysone response genes, and tumorigenesis according to Figs. 1H and EV1I,J. (**L**) Dorsal views of *ey-Flp*-MARCM-induced GFP-positive tumor-bearing larvae and the corresponding eye disc or tumor (right). (**L′**) Quantification of relative tumor size in (**L**) ($n = 6, 5, 6, 6, 6, 6$). *n* represents the number of biological replicates. Statistical analysis by ordinary one-way ANOVA test; mean ± SD. ****$p = 0.000092$, ****$p = 0.000000000012$. (**L″**) Quantification of larvae pupation rate in (**L**). Scale bars: 100 μm (**D**, **F–J**), 200 μm (**E**, **L**).

© The Author(s)

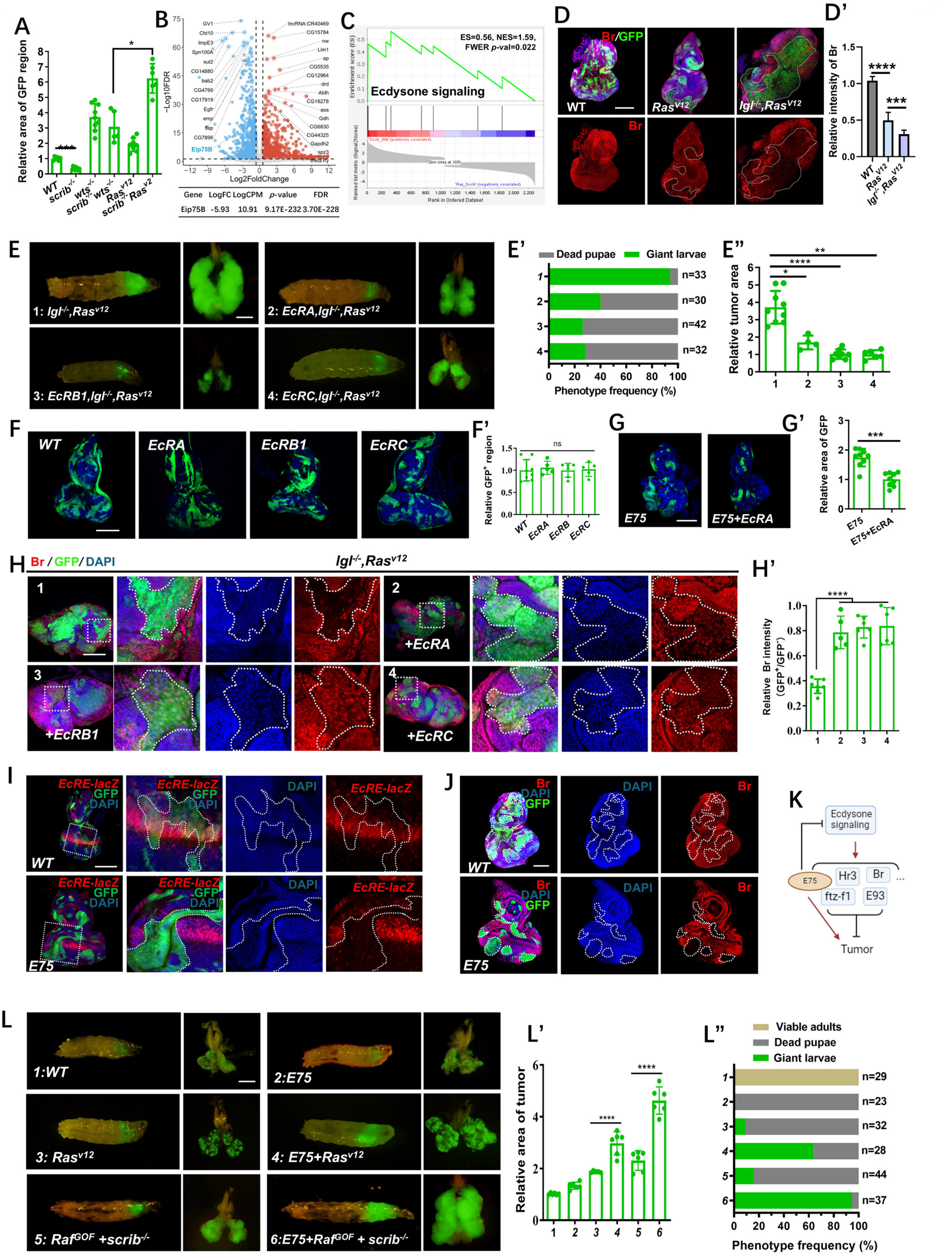
A
Relative area of GFP region
WT
scrib-/-
wts-/-
scrib-/- wts-/-
Rasv12
scrib-/- Rasv12
B
-Log10FDR
Log2FoldChange
Eip75B
Gene LogFC LogCPM p-value FDR
Eip75B -5.93 10.91 9.17E-232 3.70E-228
C
ES=0.56, NES=1.59, FWER p-val=0.022
Ecdysone signaling
Rank in Ordered Dataset
D
Br/GFP
Br
WT
Rasv12
lgl-/-,Rasv12
D'
Relative intensity of Br
E
1: lgl-/-,Rasv12
2: EcRA,lgl-/-,Rasv12
3: EcRB1,lgl-/-,Rasv12
4: EcRC,lgl-/-,Rasv12
E'
Dead pupae
Giant larvae
n=33
n=30
n=42
n=32
Phenotype frequency (%)
E''
Relative tumor area
F
WT
EcRA
EcRB1
EcRC
F'
Relative GFP+ region
ns
G
E75
E75+EcRA
G'
Relative area of GFP
H
Br / GFP / DAPI
lgl-/-,Rasv12
+EcRA
+EcRB1
+EcRC
H'
Relative Br intensity (GFP+/GFP-)
I
EcRE-lacZ
GFP
DAPI
WT
E75
J
Br
DAPI
GFP
WT
E75
K
Ecdysone signaling
E75
Hr3
Br
ftz-f1
E93
Tumor
L
1:WT
2:E75
3: Rasv12
4: E75+Rasv12
5: RafGOF +scrib-/-
6:E75+RafGOF + scrib-/-
L'
Relative area of tumor
L''
Viable adults
Dead pupae
Giant larvae
n=29
n=23
n=32
n=28
n=44
n=37
Phenotype frequency (%)

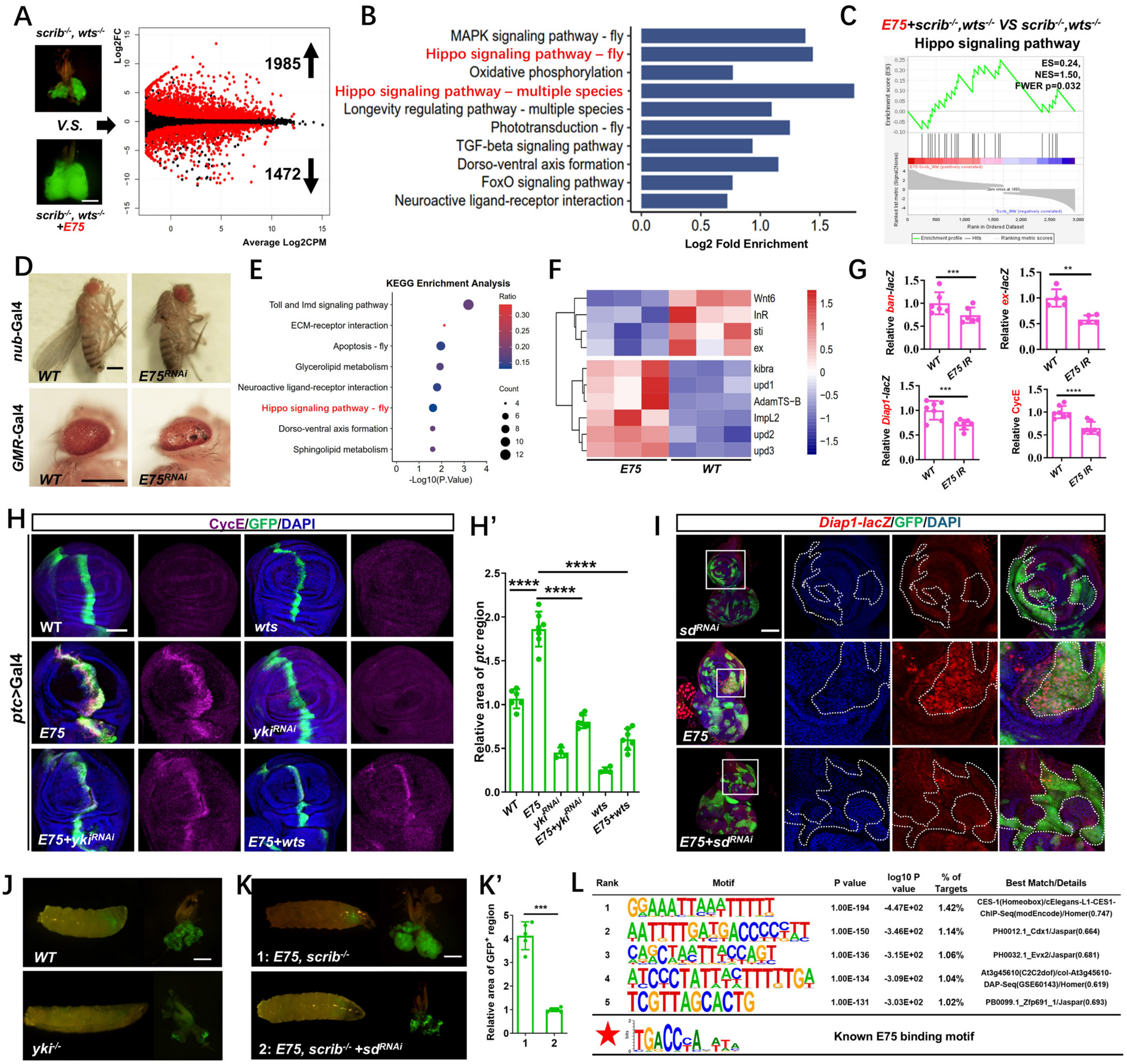

Figure EV2. ***E75* overexpression induces Hippo pathway-dependent growth and tumorigenesis.**

(**A**) MA (Minus-versus-Add) plot of *scrib*$^{-/-}$,*wts*$^{-/-}$ and *E75,scrib*$^{-/-}$,*wts*$^{-/-}$ tumors. (**B**) Enrichment analysis of signaling pathway in *scrib*$^{-/-}$,*wts*$^{-/-}$ and *E75,scrib*$^{-/-}$,*wts*$^{-/-}$ tumors. (**C**) GSEA enrichment of Hippo signaling-related genes in *scrib*$^{-/-}$,*wts*$^{-/-}$ and *E75, scrib*$^{-/-}$,*wts*$^{-/-}$ tumors. The FWER $p < 0.05$ served as the significance threshold. (**D**) Light micrographs of the adult wings (top) and eyes bearing the indicated genotypes. (**E**) KEGG enrichment of signaling pathways in *scrib*$^{-/-}$,*wts*$^{-/-}$ and *E75, scrib*$^{-/-}$,*wts*$^{-/-}$ tumors. Terms that satisfied the hypergeometric distribution were considered significant ($p < 0.05$). (**F**) Heatmap profiles of Hippo signaling-related genes in wild-type (WT) and *E75* overexpression wing discs. (**G**) Quantification of relative staining intensity of GFP positive (E75 knockdown) and negative (wild type) regions for corresponding antibodies in Fig. 3D ($n = 6$, 6 for *ban-lacZ*, $n = 6$, 6 for *ex-lacZ*, $n = 7$, 7 for *Diap1-lacZ*, $n = 7$,7 for CycE). *n* represents the number of biological replicates. Statistical analysis by paired students' *t*-test; mean ± SD. ****p* = 0.0009, ****p* = 0.0009, ****p* = 0.0002, *****p* = 0.000007. (**H**) Confocal image of Cyclin E (CycE) antibody staining of wing discs bearing the indicated genotypes. (**H'**) Quantification of the relative size of *ptc* region of **H** ($n = 6$, 7, 4, 7, 4, 7). *n* represents the number of biological replicates. Statistical analysis by ordinary one-way ANOVA test; mean ± SD. *****p* = 0.00000019, *****p* = 0.0000024, *****p* = 0.00000086. (**I**) Confocal images of *ey-Flp*-MARCM-induced GFP-positive mosaic clones stained with anti-β-galactosidase antibody for the *Diap1-LacZ* staining. (**J, K**) Dorsal views of *ey-Flp*-MARCM-induced GFP-positive tumor-bearing larvae and the corresponding dissected eye disc or tumor. Quantification of relative tumor size of GFP positive mosaics clones (**K'**, $n = 5$, 6). *n* represents the number of biological replicates. Statistical analysis by students' *t*-test; mean ± SD. ****p* = 0.004. (**L**) HOMER motif analysis of *E75* novel binding motifs in wing pouch region. ZOOPS scoring and hypergeometric distribution test were considered collectively, and motifs with $p < 0.0000001$ were considered significantly enriched. Scale bars: 100 μm (**H, I**), 200 μm (**A, D, J, K**).

 © The Author(s)

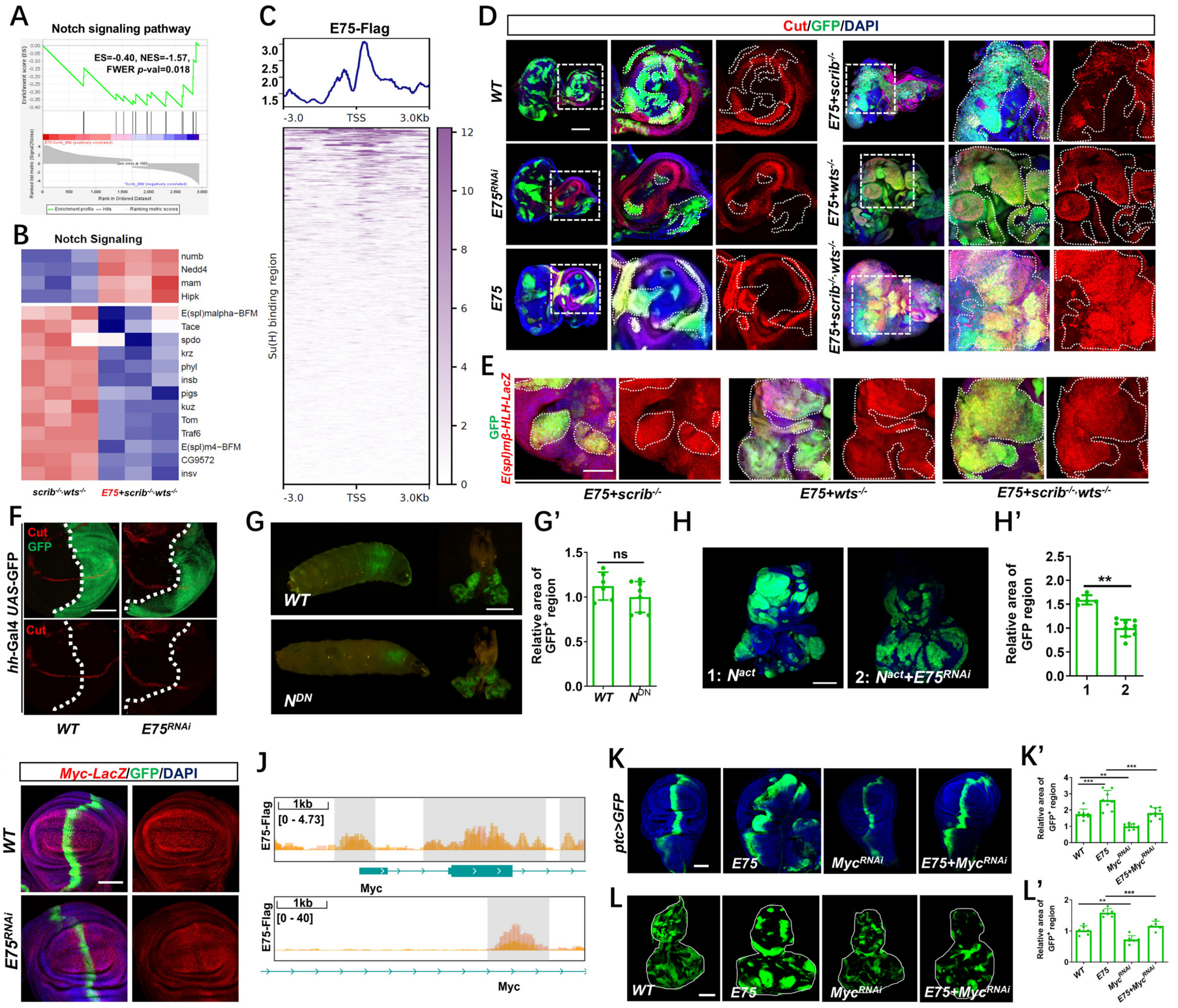

Figure EV3. E75 positively regulates the Notch pathway.

(**A**, **B**) GSEA enrichment (**A**) and heatmap profiles (**B**) of Notch signaling-related genes in *scrib*$^{-/-}$, *wts*$^{-/-}$ and *E75, scrib*$^{-/-}$, *wts*$^{-/-}$ tumors. The FWER $p < 0.05$ served as the significance threshold. (**C**) Line plots of the average CUT&Tag signal of E75 (top panel) and the heatmaps of the CUT&Tag signals of Su(H) in *Drosophila* (bottom panel). CUT&Tag signals are displayed within a region spanning ±3 kb around all canonical transcription start sites (TSS) genome-wide. (**D**, **E**) Representative confocal images of Cut (**D**) and *E(spl)mβ-HLH-lacZ* (**E**) staining in *ey-Flp*-MARCM-induced tumors and clones with indicated genotype. (**F**) Confocal images of the wing disc of control or with *E75* knockdown under the control of *hh* promoter stained with anti-Cut antibody. (**G**) Dorsal views of *ey-Flp*-MARCM-induced GFP-positive clonal-bearing larvae and the corresponding dissected eye disc. Quantification of the relative size of GFP positive mosaic clones (**K'**, $n = 6, 8$). n represents the number of biological replicates. Statistical analysis by Student's t-test; mean ± SD. ns not significant, $p = 0.196$. (**H**) Confocal images of *ey-Flp*-MARCM-induced GFP-positive mosaic clones and corresponding quantification data (**H'**, $n = 5, 9$). n represents the number of biological replicates. Statistical analysis by Student's t-test; mean ± SD. $^{**}p = 0.0044$. (**I**) Representative confocal images of *Myc-lacZ* staining in wing discs of wild-type and *E75* knockdown. (**J**) Browser shots of *E75* CUT&Tag signal at the regulatory region of *Myc*. (**K**, **L**) Representative confocal images of the wing (**K**) and eye discs (**L**) with indicated genotypes. (**K'**, **L'**) Quantification of relative GFP region of **K** and **L** (**K'**, $n = 8, 8, 9, 9$; **L'**, $n = 6, 6, 7, 5$). n represents the number of biological replicates. Statistical analysis by ordinary one-way ANOVA test; mean ± SD. (**K'**) $^{***}p = 0.0004$, $^{**}p = 0.0013$, $^{***}p = 0.00076$. (**L'**) $^{****}p = 0.0048$, $^{***}p = 0.0003$. Scale bars: 100 μm (**D–F**, **H**, **I**, **K**, **L**), 200 μm (**G**).

© The Author(s)

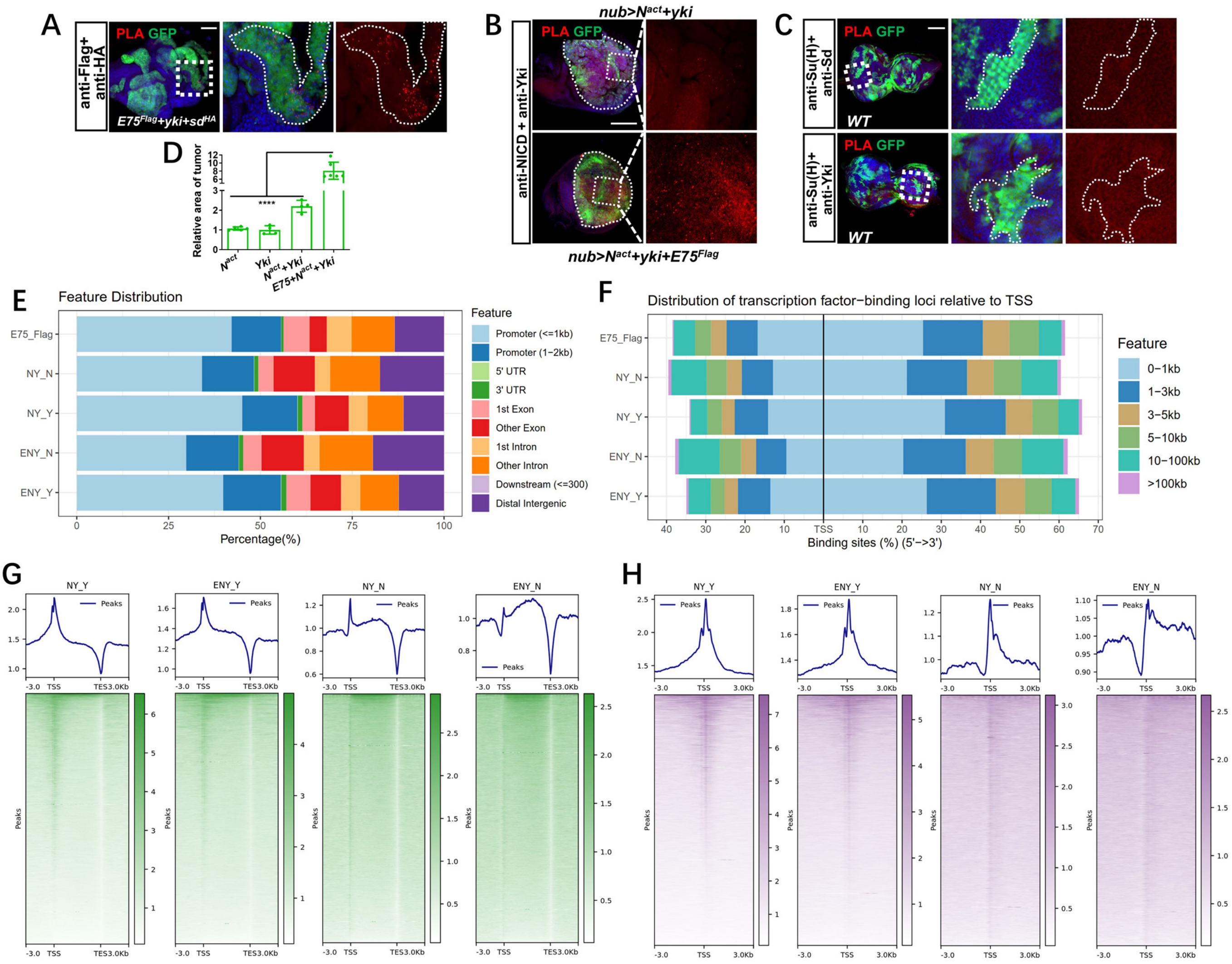

Figure EV4. E75 regulates the transcription of the Hippo and Notch pathway target genes.

(**A**) PLA was performed on eye discs bearing *ey-Flp*-MARCM-induced *E75*^{Flag}, *Yki*, and *Sd*^{HA} co-expressed clones to test close-proximity interactions between E75 and Sd. (**B**) PLA was performed on wing discs with *Yki* and *Sd*^{HA} co-expression under the control of *nub* promoter, with or without *E75*^{Flag} expression, to test close-proximity interactions between NICD and Yki. (**C**) PLA was performed on eye discs bearing *ey-Flp*-MARCM-induced WT clones to test close-proximity interactions between Su(H) and Sd, as well as Su(H) and Yki. (**D**) Quantification of relative tumor size of GFP-positive tumor clones in Fig. 5I ($n = 5, 4, 4, 6$). *n* represents the number of biological replicates. Statistical analysis by ordinary one-way ANOVA test; mean ± SD. ****$p = 0.0000006$, ****$p = 0.0000011$, ****$p = 0.0000112$. Error bars from left to right, 0.075, 0.18, 0.26, and 2.54. (**E**) Feature distribution of genomic annotations of CUT&Tag in E75 peaks (E75_Flag), NICD peaks of *N*^{act} and *Yki* overexpression (NY_N), Yki peaks of *N*^{act} and *Yki* overexpression (NY_Y). (**F**) Distribution of binding loci relative to TSS of peaks mentioned in (**D**). (**G**) Binding profiles and heatmaps of NICD and Yki in *N*^{act}*,Yki* and *E75, N*^{act}*,Yki* tumors. CUT&Tag signals are displayed within a region spanning −3 kb around all canonical TSS and +3 kb around all canonical transcription end sites (TES). (**H**) Binding profiles and heatmaps of NICD and Yki in *N*^{act}*,Yki* and *E75, N*^{act}*,Yki* tumors. CUT&Tag signals are displayed within a region spanning −3 kb and +3 kb around all canonical TSS. Scale bars: 100 μm (**A–C**).

 © The Author(s)

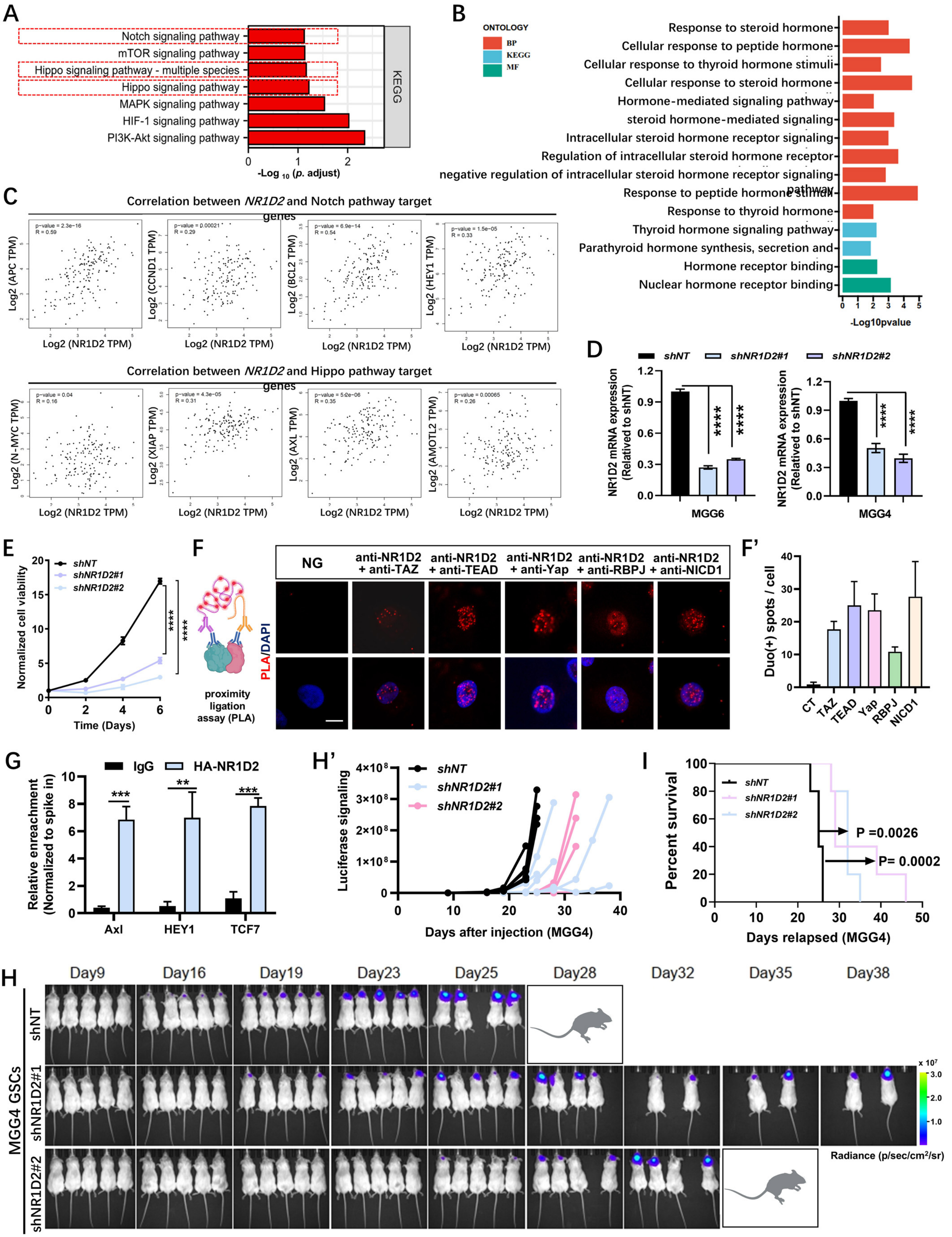
A
Notch signaling pathway
mTOR signaling pathway
Hippo signaling pathway - multiple species
Hippo signaling pathway
MAPK signaling pathway
HIF-1 signaling pathway
PI3K-Akt signaling pathway
KEGG
-Log 10 (p. adjust)
B
ONTOLOGY
BP
KEGG
MF
Response to steroid hormone
Cellular response to peptide hormone
Cellular response to thyroid hormone stimuli
Cellular response to steroid hormone
Hormone-mediated signaling pathway
steroid hormone-mediated signaling
Intracellular steroid hormone receptor signaling
Regulation of intracellular steroid hormone receptor
negative regulation of intracellular steroid hormone receptor signaling pathway
Response to peptide hormone stimuli
Response to thyroid hormone
Thyroid hormone signaling pathway
Parathyroid hormone synthesis, secretion and
Hormone receptor binding
Nuclear hormone receptor binding
-Log10pvalue
C
Correlation between NR1D2 and Notch pathway target genes
Log2 (APC TPM)
Log2 (CCND1 TPM)
Log2 (BCL2 TPM)
Log2 (HEY1 TPM)
Log2 (NR1D2 TPM)
Correlation between NR1D2 and Hippo pathway target genes
Log2 (N-MYC TPM)
Log2 (XIAP TPM)
Log2 (AXL TPM)
Log2 (AMOTL2 TPM)
D
shNT
shNR1D2#1
shNR1D2#2
NR1D2 mRNA expression (Relatived to shNT)
MGG6
MGG4
E
Normalized cell viability
Time (Days)
F
proximity ligation assay (PLA)
PLA/DAPI
NG
anti-NR1D2 + anti-TAZ
anti-NR1D2 + anti-TEAD
anti-NR1D2 + anti-Yap
anti-NR1D2 + anti-RBPJ
anti-NR1D2 + anti-NICD1
F'
Duo(+) spots / cell
CT
TAZ
TEAD
Yap
RBPJ
NICD1
G
IgG
HA-NR1D2
Relative enreachment (Normalized to spike in)
Axl
HEY1
TCF7
H'
Luciferase signaling
Days after injection (MGG4)
I
Percent survival
P =0.0026
P= 0.0002
Days relapsed (MGG4)
H
Day9
Day16
Day19
Day23
Day25
Day28
Day32
Day35
Day38
MGG4 GSCs
shNT
shNR1D2#1
shNR1D2#2
Radiance (p/sec/cm²/sr)

© The Author(s)

Figure EV5. Silencing *NR1D2* suppressed glioblastoma stem cell-driven tumor growth.

(**A**) KEGG analysis indicated enrichment of Hippo and Notch signaling in *NR1D2*-depleted U87 MG cells. Terms satisfying the hypergeometric distribution were considered significant ($p < 0.05$). (**B**) KEGG and GO analyses indicated the enrichment of multiple terms associated with hormone regulation in *NR1D2*-depleted U87 cells. Terms satisfying the hypergeometric distribution were considered significant ($p < 0.05$). (**C**) Pair-wise gene correlation analysis of *NR1D2* and target genes of Notch pathway (top panels) and Hippo pathway (bottom panels) in GBM by GEPIA2. (**D**) Relative mRNA expression of *NR1D2* in GSCs and *NR1D2*-depleted cells. $n = 3$ for each sample. *n* represents the number of technical replicates. Statistical analysis by two-tailed Student's *t*-tests; mean + SD. ****$p = 0.0000000022$, ****$p = 0.00000001$, ****$p = 0.000011$, ****$p = 0.0000033$. (**E**) Relative cell viability of MGG4 of shNT and *shNR1D2*. $n = 3$ for each sample. *n* represents the number of technical replicates. Statistical analysis by two-way repeated measures ANOVA with Dunnett's multiple hypothesis test correction; mean ± SD. ****$p = 0.0000039$, ****$p = 0.\ 0.000006$. (**F**) PLA working model (left). PLA analysis in U87 MG cells to test close-proximity interactions between NR1D2 and TAZ, TEAD, Yap, RBPJ, and NICD1 (right). (**F'**) Quantification of PLA signal intensity in (**E**) ($n = 7, 6, 9, 6, 6, 6$); mean + SD. *n* represents the number of biological replicates. (**G**) CUT&Tag qPCR analysis of *Axl*, *HEY1*, and *TCF7* in U87 MG. U87 cells transfected with *HA-NR1D2* were used for HA enrichment quantification on promoter region (−500 to 0). $n = 3$ for each sample. *n* represents the number of technical replicates. Statistical analysis by two-tailed Student's *t*- tests; mean + SD. ***$p = 0.0003$, **$p = 0.0042$, ***$p = 0.0001$. (**H**) In vivo bioluminescence imaging of NSG mice bearing tumors on the 9th, 16th, 19th, 23rd, 25th, 28th, 32nd, 35th, and 38th day after MGG4 GSCs were injected into the mice brains. The injected MGG4 GSCs were transfected with *shNT*, *shNR1D2#1*, or *shNR1D2#2*, respectively. (**H'**) Quantification of tumor size by in vivo luciferase assays. (**I**) Survival curve of NSG mice bearing intracranial tumors from MGG4 GSCs transfected with *shNT*, *shNR1D2#1*, or *shNR1D2#2*, respectively. Statistical analysis by log-rank (Mantel-Cox) test. **$p = 0.0026$, **$p = 0.0002$. Scale bar: 10 µm (**F**).

 © The Author(s)
